# Integrated Metabolo-Proteomic Approach to Decipher the Mechanisms by Which Wheat QTL (*Fhb1*) Contributes to Resistance against *Fusarium graminearum*

**DOI:** 10.1371/journal.pone.0040695

**Published:** 2012-07-12

**Authors:** Raghavendra Gunnaiah, Ajjamada C. Kushalappa, Raj Duggavathi, Stephen Fox, Daryl J. Somers

**Affiliations:** 1 Plant Science Department, McGill University, Ste. Anne de Bellevue, Quebec, Canada; 2 Animal Science Department, McGill University, Ste. Anne de Bellevue, Quebec, Canada; 3 Agriculture and Agri-Food Canada, Winnipeg, Manitoba, Canada; 4 Vineland Research and Innovation Center, Vineland, Ontario, Canada; Seoul National University, Republic of Korea

## Abstract

**Background:**

Resistance in plants to pathogen attack can be qualitative or quantitative. For the latter, hundreds of quantitative trait loci (QTLs) have been identified, but the mechanisms of resistance are largely unknown. Integrated non-target metabolomics and proteomics, using high resolution hybrid mass spectrometry, were applied to identify the mechanisms of resistance governed by the fusarium head blight resistance locus, *Fhb1,* in the near isogenic lines derived from wheat genotype Nyubai.

**Findings:**

The metabolomic and proteomic profiles were compared between the near isogenic lines (NIL) with resistant and susceptible alleles of *Fhb1* upon *F. graminearum* or mock-inoculation. The resistance-related metabolites and proteins identified were mapped to metabolic pathways. Metabolites of the shunt phenylpropanoid pathway such as hydroxycinnamic acid amides, phenolic glucosides and flavonoids were induced only in the resistant NIL, or induced at higher abundances in resistant than in susceptible NIL, following pathogen inoculation. The identities of these metabolites were confirmed, with fragmentation patterns, using the high resolution LC-LTQ-Orbitrap. Concurrently, the enzymes of phenylpropanoid biosynthesis such as cinnamyl alcohol dehydrogenase, caffeoyl-CoA *O*-methyltransferase, caffeic acid *O*-methyltransferase, flavonoid *O*-methyltransferase, agmatine coumaroyltransferase and peroxidase were also up-regulated. Increased cell wall thickening due to deposition of hydroxycinnamic acid amides and flavonoids was confirmed by histo-chemical localization of the metabolites using confocal microscopy.

**Conclusion:**

The present study demonstrates that the resistance in *Fhb1* derived from the wheat genotype Nyubai is mainly associated with cell wall thickening due to deposition of hydroxycinnamic acid amides, phenolic glucosides and flavonoids, but not with the conversion of deoxynivalenol to less toxic deoxynivalenol 3-*O*-glucoside.

## Introduction

Disease resistance in plants can be broadly classified as qualitative and quantitative. Qualitative resistance is generally governed by mono or oligo genes and imparts complete resistance. Significant advances have been made in the past few decades in understanding the defense mechanisms associated with qualitative resistance, and many genes governing resistance have been identified and used in plant improvement. On the other hand, the quantitative resistance is generally governed by polygenes and imparts partial but durable resistance. Due to its genetic complexity, the progress in the characterization of quantitative defense mechanisms has been slower. The use of DNA markers has led to the identification of quantitative trait loci (QTL) governing partial resistance [1]. Biotic stress resistance QTLs have been identified in several crop diseases such as late blight of potato (*Phytophthora infestans)* [2], rice blast (*Magnaporthe grisea*) [3], fusarium head blight (FHB) (*Fusarium graminearum*) [4] and cereal rusts (*Puccinia* spp.) [5]. These QTLs generally co-localize several genes and the cloning of QTL to identify all the co-localizing genes is a difficult task. Hence, the biochemical mechanisms by which QTLs drive disease resistance are largely unknown. Identification of specific defense mechanisms and genes associated with QTLs can lead to the pyramiding of suitable alleles to enhance resistance in elite cultivars.

Fusarium head blight (FHB) caused by *Fusarium graminearum* Schwabe (Teleomorph: *Gibberella zeae* (Schwein.) Petch) is a devastating disease of wheat and barley. FHB causes severe economic damage by reducing the grain yield and also deteriorates the grain quality by contaminating with trichothecene mycotoxins. Deoxynivalenol (DON), a type B trichothecene produced by *F. graminearum* is highly toxic to animals at very low concentrations [6] and is also a pathogen virulence factor [7]. The use of resistant genotypes is considered to be the best practical approach to manage FHB. Resistance to FHB in wheat is quantitative and is governed by polygenes [8]. The resistance has been classified into five types [9], however, only three types: type I (resistance to initial infection of spikelets), type II (resistance to spread of pathogen within spike) [10] and type III (resistance to DON) [11] have been extensively used. More than one hundred FHB resistance associated QTLs have been identified in wheat [4]. The major QTLs mapped on chromosomes 3BS, 4B, 5A, and 6B have been validated and used in marker-assisted selections. However, the resistance mechanisms governed by these QTLs are unknown, except partially for the QTL on 3BS.

The major FHB resistance QTL on 3BS, referred as *Fhb1*, explained up to 60% of the phenotypic variation for type II FHB resistance [12], [13]. It has been speculated that, *Fhb1* derived from Sumai-3, either encodes or regulates the expression of UDP glucosyltransferase that converts DON to DON-3-*O*-glucoside (D3G) [14], [15]. QTL specific transcriptome analysis of *Fhb1* locus, derived from genotype Sumai-3, showed greater accumulation of ten transcripts, including two cell wall biogenesis and two of general defense mechanisms [16]. Several other constitutive and induced chemical and structural host defense mechanisms have been documented against *Fusarium* infection [17].

Non-target metabolomics has been applied to study the mechanisms of resistance in wheat [18], [19] and barley [20]–[23] against *F. graminearum*. The quantitative resistance in barley and wheat was associated with the activation of phenylpropanoid, terpenoid and fatty acid metabolic pathways, in addition to the detoxification of DON to D3G. The metabolites of these pathways are involved in plant defense signaling, antimicrobial and cell wall strengthening properties. Non-target proteomics, based on 2D gel electrophoresis combined with LC-MS-MS, has also been applied to explain the resistance mechanisms against *F. graminearum* in barley [24], [25] and wheat [26], [27]. Proteomics revealed diverse mechanisms of resistance such as oxidative burst, oxidative stress response and induction of PR proteins. Non-target metabolomics combined with proteomics could enable the identification of key metabolites and proteins, which are the end products of gene expression, associated with *Fhb1*, explaining specific mechanisms of resistance.

Nyubai is a moderately resistant Japanese cultivar that explained up to 30% phenotypic variance for type II resistance, and is also a potential alternative source to widely used Sumai-3 for FHB resistance [28], [29]. *Fhb1,* from Nyubai was mapped to the same locus as that of *Fhb1* derived from Sumai-3 but with different allele sizes [13]. The objective of the present study was to investigate the resistance mechanisms in wheat to the spread of FHB within spike, governed by the *Fhb1*, based on non-target metabolomics and proteomics tools. The use of NILs minimizes the genetic background effects and better explains the resistance mechanisms governed by a specific locus for which the NILs are differing. Hence two NILs, with resistant (NIL-R) and susceptible (NIL-S) alleles of *Fhb1*, derived from Nyubai were used to investigate the mechanism of resistance governed by a QTL.

## Results

### FHB Disease Severity of NILs

**Figure 1:**
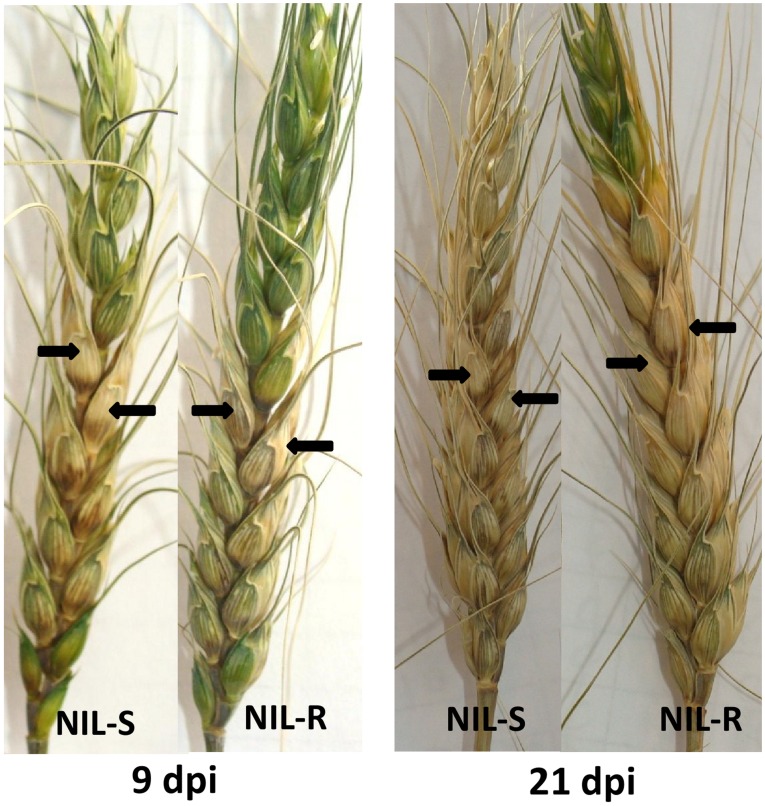
*Fusarium graminearum* infected spikes of wheat NILs with resistant and susceptible alleles of *Fhb1*, at 9 dpi and 21 dpi. Arrows indicate the site of point inoculation, NIL-R = NIL with resistant allele of *Fhb1*, NIL-S =  NIL with susceptible allele of *Fhb1.*

FHB disease severity on NILs, with resistant and susceptible alleles of *Fhb1*, was assessed following point inoculation of a pair of middle spikelets with spores of *F. graminearum*. Dark brown discoloration of inoculated spikelets due to necrotropic feeding by the pathogen was observed at 3 days post inoculation (dpi) in both resistant and susceptible NILs. By 9 dpi, the non-inoculated spikelets above and below the point of inoculation were bleached and started drying up (Fig.1). Both dark brown and bleached spikelets were considered diseased. By 21 days, all most all the spikelets in a spike were diseased in NIL-S while in NIL-R significantly less number of spikelets was diseased. Area under disease progress curve (AUDPC), calculated based on the proportion of spikelets diseased, was the highest in NIL-S (AUDPC = 10.45), significantly (*P*<0.001) differing from NIL-R (AUDPC  = 6.08).

### Differential Metabolic Profiles of Wheat NILs with Resistant and Susceptible *Fhb1* Alleles

Non-target metabolic profiling of rachis and spikelets of two NILs, with resistant and susceptible alleles of *Fhb1* inoculated with *F. graminearum* and water (mock), identified several resistance related (RR) metabolites. Initially, we examined constitutive metabolites, using only the mock inoculated NIL-R and NIL-S. In rachises, 271 metabolites were differentially accumulated between NILs, of which 235 had higher abundance in NIL-R. The latter were designated as the resistance related constitutive (RRC) metabolites (Table 1, 2 & S1). In spikelets, only 123 metabolites were differentially accumulated between NILs, of these 71 metabolites were classified as RRC metabolites (Table 1, 2 & S2).

**Table 1:** Fusarium head blight resistance related metabolites identified in rachis and spikelets of wheat NIL with resistant *Fhb1* allele upon *F. graminearum* or mock inoculation.

Observed mass (Da)	Putative name£	Fold change@	
		Rachis	Spikelets
***Phenylpropanoids: Phenolics and Lignans***			
148.0527	*trans*-Cinnamic acid	1.27*(RRI)	2.0* (RRI)
165.0797	L-Phenylalanine		1.56* (RRI)
208.0722	Sinapaldehyde	2.22*(RRI)	3.36* (RRI)
224.0700	Sinapic acid	2.6*(RRC)	
320.0888	4-Coumaroylshikimate	85.1*(PRr;***RRI***)	
338.0993	4-Coumaroylquinate	43.1*(PRr;***RRI***)	
342.1002	β-D-glucopyranosyl-caffeic acid	2.6*(RRI), 4.0*(PRr)	
342.1308	Coniferin	1.58*(RRI)	1.41* (RRI)
344.1458	Dihydroconiferyl alcohol glucoside	10**(PRr;***RRI***)	
356.1101	Ferulic acid 7-O-glucoside	28.7* (PRr;***RRI***)	
370.1265	Sinapaldehyde glucoside	93.1*(PRr;***RRI***)	1.14* (RRI)
372.1425	Syringin	1.92 *(RRI)	2.09* (RRI)
386.1210	β -D-glucopyranosyl-sinapic acid	2.8 * (RRI)	
398.1356	Deoxypodophyllotoxin	1.4*(RRI), 1.4*(RRC)	
550.2036	Medioresinol 4'-O-beta-D-glucopyranoside	1.6*(PRr;***RRI***)	
674.1447	Phyllanthusmin B	1.5*(RRC)	
686.2743	Secoisolariciresinol di-O-glucoside	1.46*(RRI)	
***Phenylpropanoids: Hydroxycinnamic acid amides***			
234.1367	*p*-Coumaroylputrescine	24.6** (PRr;***RRI***)	1.93* (RRI)
264.1473	Feruloylputrescine	407.8*(PRr;***RRI***)	
267.1268	Cinnamoyltyramine	262.8*(PRr;***RRI***)	
276.1584	*cis-p*-Coumaroylagmatine	44.3*(PRr;***RRI***)	41** (PRr;***RRI***)
306.1688	Feruloylagmatine	104.2 *(PRr;***RRI***)	
322.1321	p-Coumaroylserotonin	99.1*(PRr;***RRI***)	
338.1258	Caffeoylserotonin	2.45*(RRI)	16.1 *(PRr;***RRI***)
352.1421	Feruloylserotonin	1194.8*(PRr;***RRI***)	30.7* (PRr;***RRI***)
***Phenylpropanoids: Flavonoids***			
282.0887	5,6-Dimethoxyflavone	126.6*(PRr;***RRI***)	
288.0622	2-hydroxyisoflavanone naringenin	16.3*(PRr;***RRI***)	
434.1219	Naringenin 7-O-β-D-glucoside	23.7*(PRr;***RRI***)	1.83* (RRI)
446.1567	5-Hydroxy-7,8-dimethoxyflavanone 5-rhamnoside	74.4*(PRr;***RRI***)	
710.2085	Kaempferol 3-rhamnoside-7-xylosyl-(1−>2)-rhamnoside	34.9*(PRr;***RRI***)	

£ Detailed compound identification is presented in Table S1 and S2.

@ Fold change calculation: were based on relative intensity of metabolites, RRC =  RM/SM, PRr =  RP/RM, RRI =  (RP/RM)/(SP/SM); PRr;RRI  =  RP/RM, PRr fold change is reported for the metabolites detected only in NIL-R (qualitative) as the RRI fold change would be infinity.

*t test significance at *P*<0.05, ** *t* test significance at *P*<0.01, *** *t* test significance at *P*<0.001.

NIL is Near isogenic line, Da: Daltons, RRC is Resistance related constitutive, RRI is Resistance related induced, PRr is Pathogenesis related metabolite detected in resistant NIL; RP is resistant NIL with pathogen inoculation, RM is resistant NIL with mock inoculation, SP is susceptible NIL with pathogen inoculation, SM is susceptible NIL with mock inoculation.

**Table 2:** Putatively identified FHB resistance related metabolites, other than phenylpropanoids, in rachis and spikelets of resistant wheat NIL with *Fhb1* upon *F. graminearum* or mock inoculation.

Observed mass (Da)	Putative name£	Fold change@	
		Rachis	Spikelets
***Plant signaling molecules (Jasmonic acid biosynthesis)***			
210.1255	Jasmonic acid	1.14**(RRI)	1.52* (RRI)
278.2257	α-Linolenate		2.79** (RRI)
280.2403	Linoleic acid	1.5*(RRC)	2.61* (RRI)
296.2351	9S-hydroxy-10E,12Z-octadecadienoic acid (9(S)-HODE)	1.6*(RRC)	
309.1935	Jasmonoyl-valine	79.1*(PRr;***RRI***)	
310.2138	13(S)-Hydroperoxylinolenic acid	1.5*(RRC)	
323.2093	(+)-7-iso-Jasmonoyl-L-isoleucine	1.76*(RRI)	1.82*(RRI)
***Plant signaling molecules (Salicylic acid biosynthesis)***			
138.0320	Salicylic acid	3.2*(PRr;***RRI***)	
300.0841	Salicylic acid 2-O-β-D-glucoside	1.03*(RRI)	
***Terpenoids***			
248.1419	Abscisic aldehyde	163.1*(PRr;***RRI***)	1.22* (RRI)
250.1568	Xanthoxin	2.68*(RRI)	
250.1568	Abscisic alcohol	2.67*(RRI)	
280.1309	8'-Hydroxyabscisate	529.4*(PRr;***RRI***)	
344.1471	Iridotrial glucoside		12.3* (PRr;***RRI***)
346.1260	Aucubin	2.6*(PRr;***RRI***)	
346.1261	Deutzioside	1.56*(RRI)	
360.1416	7-Deoxyloganate	1.4*(RRC)	1.30** (RRI)
390.1508	Loganin	1.70*(RRI)	
406.1467	10-Hydroxyloganin	1.01*(RRI)	
426.1881	Abscisic acid glucose ester	189.1*(PRr;***RRI***)	
***Indole alkaloids***			
292.1560	16-epivellosimine	35.3*(PRr;***RRI***)	
350.1636	Vomilenine	1054.4**(PRr;***RRI***)	8.21** (RRI)
***Methionine biosynthesis***			
384.1209	2-S-adenosyl-L-homocysteine	132.6*(PRr;***RRI***)	
399.1437	S-adenosyl-L-methionine	Inf*(***RRI***)	

£ Detailed compound identification is presented in Table S1 and S2.

@ Fold change calculation: were based on relative intensity of metabolites, RRC =  RM/SM, PRr =  RP/RM, RRI =  (RP/RM)/(SP/SM); PRr;RRI  =  RP/RM, PRr fold change is reported for the metabolites detected only in NIL-R.

*t test significance at *P*<0.05, ** *t* test significance at *P*<0.01, *** *t* test significance at *P*<0.001.

NIL =  Near isogenic line, Da: Daltons, RRC  =  Resistance related constitutive, RRI  =  Resistance related induced, PRr = Pathogenesis related metabolite detected in resistant NIL; RP =  resistant NIL with pathogen inoculation, RM =  resistant NIL with mock inoculation, SP =  susceptible NIL with pathogen inoculation, SM =  susceptible NIL with mock inoculation.

**Figure 2:**
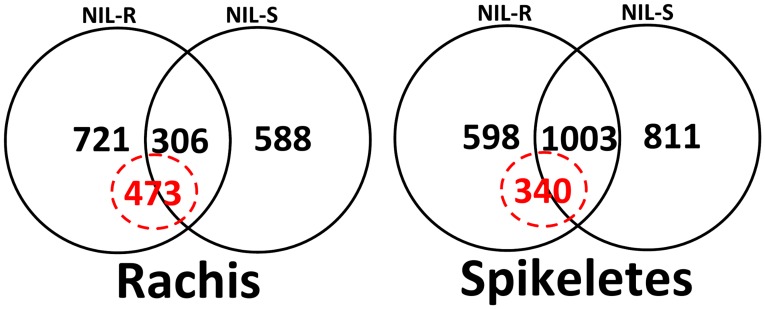
Venn diagram differentially accumulated metabolites (*P*<0.05) detected in wheat NILs with resistant and susceptible alleles of *Fhb1* upon *F. graminearum* or mock inoculation. Numbers in dotted circle =  number of resistance related induced metabolites (RRI).

Following *F. graminearum* inoculation 1309 metabolites were differentially accumulated in rachises of either NIL-R or NIL-S (Fig.2). The metabolites that were either induced only in NIL-R (qualitative) or induced at greater abundance in NIL-R were designated as resistance related induced (RRI) metabolites. In rachises, 473 metabolites were classified as RRI, including 314 induced only in NIL-R but not in NIL-S. In spikelets, 2412 metabolites were differentially induced in either of the NILs, of which 340 were classified as RRI metabolites, including 109 induced only in NIL-R. Comparatively, more number of RR metabolites was detected in rachises than in spikelets.

**Figure 3:**
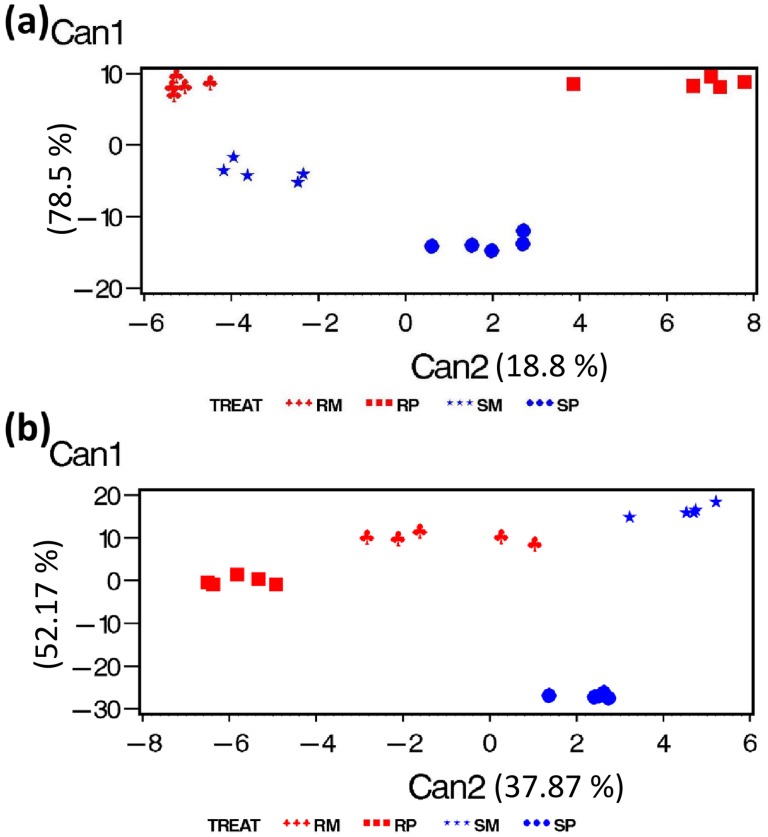
Canonical discriminant analysis of significant (*P*<0.05) metabolites in: (a) rachises and (b) spikelets of wheat NILs with resistant and susceptible alleles of *Fhb1* upon *F. graminearum* or mock inoculation. Where, RP is *F. graminearum* inoculated NIL-R, RM is mock-inoculated NIL-R, SP is *F. graminearum* inoculated NIL-S, SM is mock-inoculated NIL-S.

A data dimension reduction technique, canonical discriminant analysis, was applied to classify the treatment effects based on metabolites [19]. In rachises, a total of 672 metabolites, present in all the four treatments (excluding qualitative metabolites) and with significant treatment effects, were subjected to canonical discriminant analysis. The CAN1 vector explained 78.5% variance, and it identified the constitutive resistance function, discriminating the NIL-R from NIL-S. The CAN2 vector explained 18.8% variance, and it identified the pathogenesis function, discriminating the pathogen inoculation from mock inoculation (Fig. 3a). However, the induced resistance mechanism was not explained by any of the CAN vectors, may be because the metabolites induced only in NIL-R (qualitative) were not included in the analysis. In spikelets, a total of 693 treatment significant metabolites were subjected to canonical discriminant analysis. The CAN1 vector failed to explain any resistance function, whereas the CAN2 explained 37.87% variance, and it partially identified the constitutive resistance function by discriminating the resistant NIL from the susceptible NIL (Fig. 3b).

### Resistance Related Constitutive (RRC) Metabolites Associated with *Fhb1*

Out of 235 RRC metabolites in rachises, 19 metabolites were putatively identified based on accurate mass match (Table 1, 2 & S1): sinapic acid, 11 flavonoids, and two lignans of phenylpropanoid pathway, three fatty acids and one terpenoid. In spikelets, 10 of the 109 RRC metabolites were putatively identified, of these; seven belonged to phenylpropanoid pathway (Table 1, 2 & S1).

### Resistance Related Induced (RRI) Metabolites Associated with *Fhb1*

Among the 473 metabolites classified as RRI in rachises, 68 were putatively identified; the identity of the most significant metabolites was confirmed based on fragmentation patterns using LC-LTQ-Orbitrap (Table 1, 2 & S1). Thirty three of the identified metabolites belonged to phenylpropanoid pathway, including 9 hydroxycinnamic acid amides (HCAAs), seven flavonoids and four phenolic glycosides that are known to be involved in cell wall strengthening. Strikingly, the HCAAs, especially the *p*-coumaroylputrescine, feruloylputrescine, cis-*p*-coumaroylagmatine, cinnamoylserotonin, feruloylagmatine, *p*-coumaroylserotonin and feruloylserotonin were induced only in NIL-R (qualitative) but not in NIL-S. Similarly, among the seven identified flavonoids five (5,6-dimethoxyflavone, 2-hydroxyisoflavanone naringenin, naringenin 7-O-β-D-glucoside, 5-hydroxy-7,8-dimethoxyflavanone 5-rhamnoside and kaempferol 3-rhamnoside-7-xylosyl-(1−>2)-rhamnoside) were induced only in NIL-R. In addition, glycosides of caffeic acid, ferulic acid, sinapic acid and coniferyl alcohol were induced in NIL-R at greater abundances. Other significant RRI metabolites detected in rachises were S-adenosylmethionine and homocysteine of cysteine and methionine metabolism, fatty acids, terpenoids and alkaloids. The qualitative induction or several fold increase in abundances of HCAAs, flavonoids and glycosides of phenolic compounds, in NIL-R following pathogen inoculation, imply that *Fhb1* might mainly regulate phenylpropanoid pathway.

In spikelets, a total of 346 metabolites were classified as RRI metabolites, of which 47 were putatively identified (Table 1, 2 & S2). Twenty five of these RRI metabolites belonged to the phenylpropanoid pathway, including 11 flavonoids, 4 HCAAs, and 3 lignans. *Hydroxycinnamic acid amide: cis*-*p*-coumaroylagmatine, caffeoylserotonin and feruloylserotonin were detected only in NIL-R. Six glycerophospholipids that are involved in wax biosynthesis and get deposited at the cuticle were detected only in spikelets; these might play a significant role in type I resistance.

Necrotropic plant signaling molecule, jasmonic acid along with its amino acid conjugate: (+)-7-iso-jasmonoyl-L-isoleucine, was detected as a RRI metabolite in both rachises and spikelets. However, another amino acid conjugate jasmonoyl valine was detected only in rachises. Biotrophic plant defense signaling molecule, salicylic acid and its glucoside were detected only in rachises.

### Resistance Indicator (RI) Metabolites Induced Following Pathogen Stress

**Figure 4:**
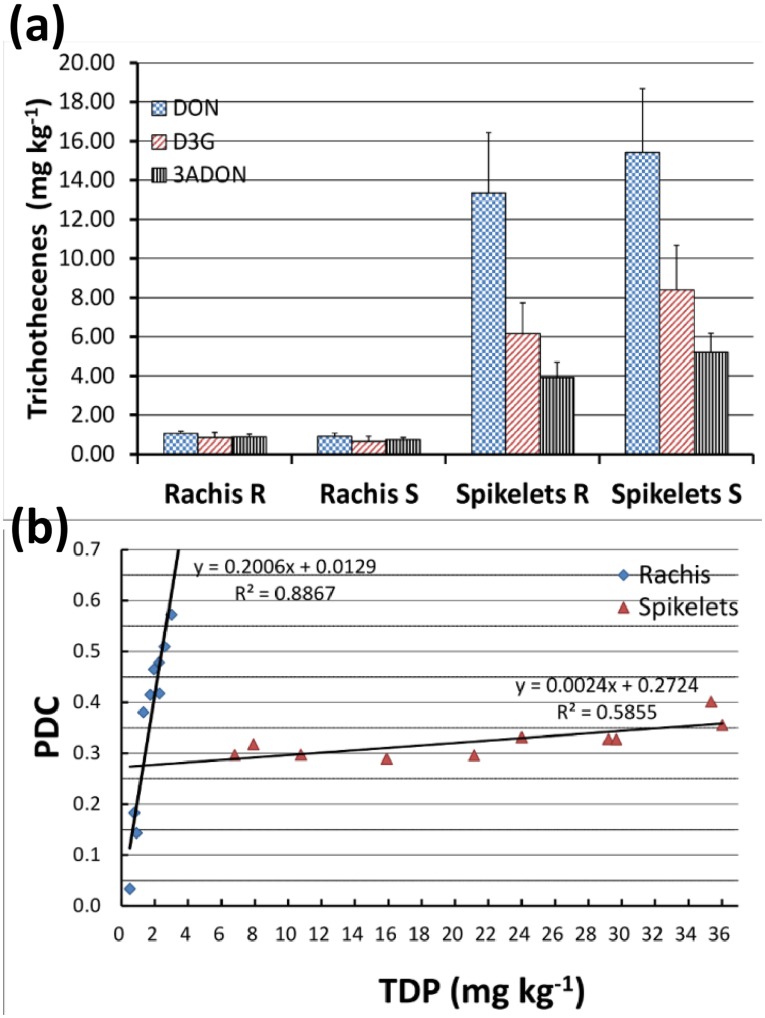
Accumulation of resistance indicator (RI) metabolites in wheat NILs with resistant and susceptible alleles of *Fhb1* inoculated with *F. graminearum* (a) Accumulation of DON, 3ADON and D3G; (b) Regression models to predict proportion of total DON converted to D3G (PDC) as a function of total DON produced (TDP). Where, DON  =  deoxynivalenol, 3ADON  = 3-acetyl-deoxynivalenol, D3G  =  DON-3-*O*-glucoside, TDP  =  total DON produced, PDC  =  proportion of DON converted to D3G.

*F. graminearum* produces DON to spread within spike, and in response, host detoxifies DON by glycosylating DON to D3G, and the latter two metabolites have been defined as resistance indicator (RI) metabolites [22]. Both DON and D3G were quantified using standard curves. The total amount of DON produced (TDP = DON + D3G) was lower (1.57 mg kg^−1^) in rachises than in spikelets (23.84 mg kg^−1^) (Table 3, Fig. 4a). Conversely, the proportion of total DON converted to D3G was higher in rachises (PDC  = 0.39) than in spikelets (Table 3), meaning the proportion of DON conversion was higher at lower concentrations of TDP (Fig. 4b). However, the amounts DON, TDP, D3G or proportion of DON converted to D3G (PDC) were not significantly different between the NIL-R and NIL-S (Table 3).

**Table 3:** DON & 3ADON accumulation and DON detoxification in wheat NILs^a^ with contrasting alleles of type II FHB resistance *Fhb1* inoculated with *F. graminearum*.

Metabolite	NIL-R			NIL-S		
	Rachis	Spikelets	*P* value^b^	Rachis	Spikelets	*P* value^b^
DON (mg kg^−1^)	1.05	13.35	0.001	0.92	15.42	0.002
D3G (mg kg^−1^)	0.84	6.15	0.01	0.66	8.42	0.009
TDP (mg kg^−1^)	1.89	19.51	0.01	1.57	23.84	0.004
PDC (D3G/TDP)	0.39	0.32	0.57	0.32	0.34	0.871

a There was no significant difference between NIL-R and NIL-S for any of the metabolites, but the spikelet metabolites were significantly different from rachis.

b *P* values were derived based on two way ANOVA NIL-R = near isogenic line- resistant, NIL-S = near isogenic line- susceptible, DON  =  Deoxynivalenol, D3G  =  Deoxynivalenol-3-*O*-glucoside, TDP  =  Total DON produced (DON + D3G), PDC  =  Proportion of DON converted, 3ADON  = 3 Acetyl.

### Histochemical Localization of HCAAs and Flavonoids

**Figure 5:**
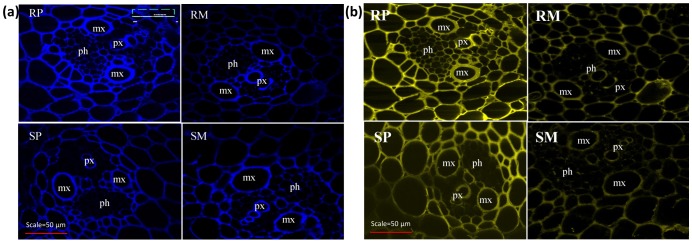
Laser scanning confocal micrographs of rachis sections, exhibiting secondary cell wall thickening, due to: a) HCAAs (blue fluorescence) and b) flavonoids (yellow fluorescence). RP is resistant NIL with *F. graminearum* (pathogen) inoculation, RM is resistant NIL with mock inoculation, SP is susceptible NIL with *F. graminearum* inoculation, SM is susceptible NIL with mock inoculation, mx is meta xylem, px is protoxylem, ph is phloem, c is cortical cells.

Following pathogen inoculation, several HCAAs and flavonoids were either induced only in the rachis of NIL-R (qualitative) or the fold changes in induction was much greater than in rachis of NIL-S. To confirm the deposition of HCAA and flavonoids at cell walls, histochemical staining technique was used to visualize the location of deposition of these metabolites. The thickening of xylem and surrounding sclerified cell walls, especially of the meta-xylem cells, was observed following *F. graminearum* inoculation. Deposition of HCAAs (blue fluorescence) and flavonoids (yellow fluorescence) were greater in pathogen treated NIL-R cells than in mock treated NIL-R and pathogen or mock treated NIL-S (Fig. 5). Blue and yellow fluorescence due to accumulation of HCAAs and flavonoids was also detected in phloem cells, but the fluorescence intensity was not visually distinct between NILs.

### *Fhb1* Specific Differential Expression of Proteins

Metabolomics of rachises revealed qualitative and quantitative accumulation of several metabolites, following pathogen inoculation. Hence, a shotgun proteomic profiling of the proteins isolated from rachises was done to further characterize the resistance mechanisms governed by *Fhb1*. A total of 512 non-redundant proteins were identified, with 0.1% protein false discovery rate (FDR) and 1.1% peptide FDR, from 30193 spectra in *F. graminearum* or mock inoculated wheat NILs with alternative alleles of *Fhb1.* Very low FDR suggests that, sufficient stringency was allowed for protein identification with at least 2 peptides/protein, >95% peptide accuracy and >99% protein accuracy. A total of 172 proteins induced upon *F. graminearum* inoculation were identified in either of the NILs. Among these, 104 proteins were identified as RRI proteins, including 13 proteins induced only in NIL-R (Table S3).

#### Resistance related induced (RRI) proteins

**Figure 6:**
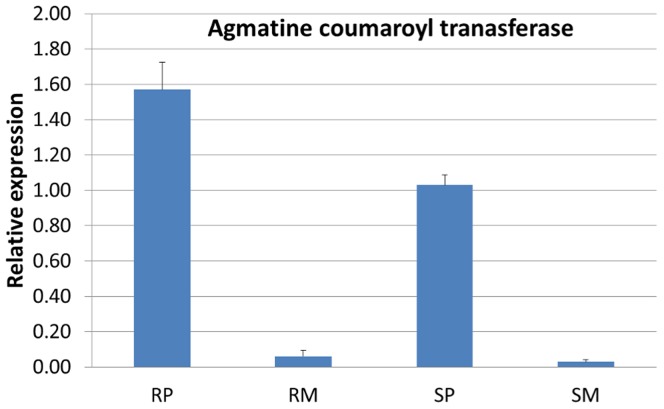
Relative transcript expression of *Triticum aestivum* agmatine coumaroyl transferase (*TaACT*) at 72 hpi in wheat NILs with resistant and susceptible alleles of *Fhb1* upon *F. graminearum* and mock inoculation. RP is resistant NIL with *F. graminearum* inoculation, RM is resistant NIL with mock inoculation, SP is susceptible NIL with *F. graminearum* inoculation, SM is susceptible NIL with mock inoculation.

The 104 proteins classified here as RRI proteins in rachises were characterized according to their role in biological process in plant system (Fig. S3). More than 50% of the RRI proteins identified here were known to be induced in response to biotic or abiotic stresses, endogenous stimulus and signal transduction, including four pathogenesis related (PR) proteins: PR-1, β-1,3- glucanases (PR-2), chitinases (PR-3) and PR-10. Thirteen proteins were related to cell death that might have been induced as an early response to necrotrophs. A total of 61 RRI proteins were mapped on to different metabolic pathways, based on KEGG (Table S3). In consistent with our metabolomics data, enzymes of cysteine and methionine metabolism: methionine synthase, S-adenosylmethionine synthase, 5,10-methylene-tetrahydrofolate reductase, and S-adenosylhomocysteine hydrolase that increase the metabolic flux towards ethylene and phenylpropanoid biosynthesis were up regulated in NIL-R. In parallel, a few phenylpropanoid pathway enzymes, such as caffeic acid-*O*-methyltransferase, caffeoyl-CoA-*O*-methyltransferase, cinnamyl alcohol dehydrogenase, peroxidases and flavonoid-*O*-methyltransferase that are involved in lignin and flavonoid biosynthesis pathway were also upregulated (Table 4). Hydroxycinnamoyl transferases, involved in HCAA biosynthesis, were detected at low stringency of 80% protein identification probability and one peptide per protein. To further confirm the expression of hydroxycinnamoyl transferases, differential transcript expression of one of the hydroxycinnamoyl trnasferases: *Triticum aestivum* agmatine counaroyl transferase (*TaACT*) was conducted. The transcript expression of *TaACT* was significantly higher in the rachis of pathogen treated NIL-R than in NIL-S (Fig. 6).

**Table 4:** Resistance related induced (RRI) proteins identified in wheat NIL with resistant allele derived from Nyubai inoculated with *F. graminearum.*

gi number	Identified Proteins (512)	RRI fold change (*P*<0.05)
***Cysteine and methionine metabolism***		
585032	Cysteine synthase	1.90
162458737	Cysteine synthase precursor	14.67
50897038	Methionine synthase	1.13
68655500	Methionine synthase 2 enzyme	1.14
115470493	Os07g0134800 (homologous to L-serine ammonia-lyase)	6.01
115589742	5,10-methylene-tetrahydrofolate reductase	1.20
115589748	S-adenosylhomocysteine hydrolase	1.18
223635282	S-adenosylmethionine synthase 1	2.60
122220777	S-adenosylmethionine synthase 3	1.99
***Phenylpropanoid biosynthesis***		
194268461	Chorismate synthase	1.67
298162735	Cinnamyl alcohol dehydrogenase	1.26
126723796	Caffeoyl-CoA O-methyltransferase	7.00
30385246	Caffeic acid O-methyltransferase	1.3
129806	Peroxidase 1	3.00
2759999	Peroxidase	1.86
57635161	Peroxidase 8	1.92
77818928	Flavonoid O-methyltransferase	1.34

@ RRI = (RP/RM)/(SP/SM). RP: resistant NIL with pathogen inoculation, RM: resistant NIL with mock inoculation, SP: susceptible NIL with pathogen inoculation, SM: susceptible NIL with mock inoculation.

## Discussion

**Figure 7:**
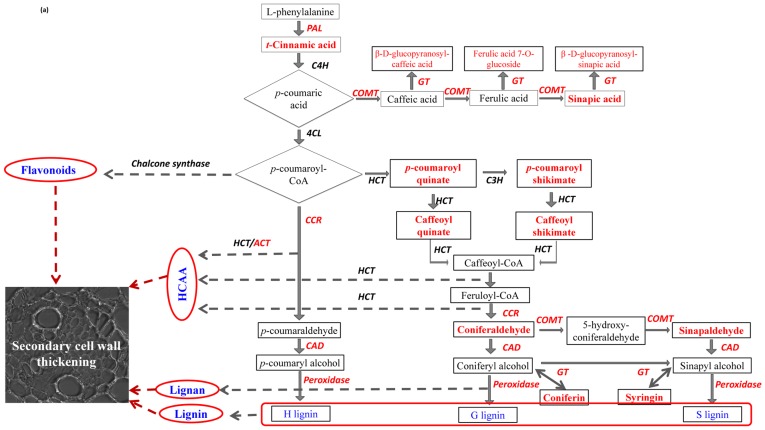
*F. graminearum* induced phenylpropanoid pathway leading to secondary cell wall thickening in rachises of wheat NIL with resistant *Fhb1* allele (Compounds and enzymes in bold/red letters are detected in the study). Where, HCAA is hydroxycinnamic acid amides; PAL is phenylalanine ammonia lyase, 4CL is 4 coumaryl ligase, C4H is trans-cinnamate 4-monooxygenase, COMT is caffeic acid 3-*O*-methyltransferase, HCT is hydroxycinnamoyltransferase, C3H is coumaroylquinate(coumaroylshikimate) 3'-monooxygenase, CCR is cinnamoyl-CoA reductase, GT is glycosymethyl transferase. Pathway adapted from http://www.genome.jp/kegg-bin/show_pathway?ko00940C00482.

**Figure 8:**
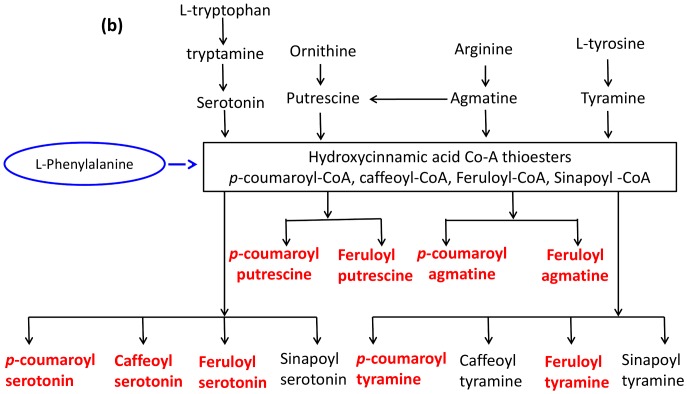
*F. graminearum* induced shunt phenylpropanoid pathway showing the synthesis of hydroxycinnamic acid amides following the conjugation of amides synthesized from amino acids with hydroxycinnamic acid CoA thioesters (Compounds in bold/red letters are detected in the study). Pathway adapted from http://pmn.plantcyc.org/ARA/NEW-IMAGE?type=PATHWAY&object=PWY-5473, 5474, 40.

Molecular marker based technology has been used to identify and introgress the disease resistance QTLs to improve resistance against biotic stresses in elite cultivars. More than one hundred FHB resistance QTLs have been identified in wheat but the host defense mechanism associated with them is largely unknown, except partially for the *Fhb1*. An integrated metabolomics and proteomics approach was used to explain the mechanisms of resistance associated with *Fhb1*, using NILs with minimum genetic background effects, derived from wheat genotype Nyubai. This study reports several RR metabolites in wheat, with confirmative identification, including *in-planta* metabolite MS/MS fragmentation based on a high resolution LC-hybrid MS, LTQ-Orbitrap (Table S1 and S2). The RR metabolites and proteins were mapped to metabolic pathways (Fig. 7, 8 and S4). The qualitative induction or several fold increases in abundances of HCAAs, flavonoids and phenolic glycosides in NIL-R, biosynthesized in phenylpropanoid pathway, and a parallel up-regulation of enzymes of methionine and phenylpropanoid pathway, clearly imply that *Fhb1* mainly regulates phenylpropanoid pathway to resist pathogen attack. Based on the identified metabolites and proteins, the plausible biochemical mechanisms of resistance against FHB, specific to the *Fhb1* derived from Nyubai, are discussed below.

### *Fhb1* is Associated with Secondary Cell Wall Thickening

After initial colonization of a spikelet, *F. graminearum* spreads to other spikelets through the cortical cells and vasculature of the rachis [30]. Wheat resists the spread of *F. graminearum* by induced chemical defenses such as cell wall thickening and biotransformation of DON to less toxic D3G [17]. Here, we provide compelling evidence that, reduced spread of the pathogen through rachis of NIL-R is mainly due to strengthening of rachis cell walls through deposition of HCAAs, flavonoids and phenolic glycosides that are synthesized via a shunt phenylpropanoid metabolism. Activation of phenylpropanoid pathway following *F. graminearum* inoculation was evident with up regulation of enzymes of methionine metabolism (Table 4) that increase metabolic flux towards phenylpropanoid biosynthesis and also enzymes of phenylpropanoid pathway. Methionine the precursor of S-adenosylmethionine is biosynthesized by S-adenosylmethionine synthase. S-adenosylmethionine, as a methylgroup donor, is needed for the biosynthesis of phenylpropanoids, leading to an increased cell wall appositions in wheat leaves following infection by powdery mildew pathogen, *Blumeria graminis* [31]. In our study, key phenylpropanoid enzymes such as caffeic acid-*O*-methyltransferase, caffeoyl-CoA-*O*-methyltransferase, cinnamyl alcohol dehydrogenase, peroxidases and flavonoid-*O*-methyltransferase were up-regulated in NIL-R. The biochemical pathways of cell wall thickening are complex and are discussed below.

#### Cell wall thickening due to deposition of HCAAs and flavonoids

Most strikingly, HCAAs of putrescine, tyramine, agmatine and serotonin were highly induced following pathogen inoculation in resistant but not in the susceptible NIL. These HCAAs, which act as phytoalexins and also deposited to strengthen cell walls, are synthesized in a shunt phenylpropanoid pathway (Fig. 8) by the condensation of hydroxycinnamoyl-CoA thioesters of the phenylpropanoid pathway with aromatic amines such as serotonin, agmatine, putrescine, spermine, spermidine and tyramine, by amine specific hydroxycinnamoyltransferases [32]–[34]. Hydroxycinnamoyl moieties of HCAAs cross link with polysaccharides, lignin and suberin of the cell wall by etheric linkage and are deposited as cell wall appositions at the inner side of plant cell walls [35]. Thickening of the xylem and surrounding cells at the rachis of NIL-R was confirmed in our study based on HCAA specific fluorescence using confocal microscopy (Fig.5a). Furthermore, the up regulation of transcript expression of agmatine coumaroyl transferase, involved in the biosynthesis of *p*-coumaroyl agmatine, was also proved based on quantitative real time PCR (Fig. 6). The *Fhb1* is physically mapped on the contig *ctg0954* which carries 41 genes. [36]. One of the gene fragment on *ctg0954,* predicted to code for protein (GenBank: *CBH32656.1*), has a functional domain of N-hydroxycinnamoyl/benzoyltransferase (searched using PSI-BLAST). It is possible that *Fhb1* from Nyubai, mainly codes for the hydroxycinnamoyl transferases involved in HCAA biosynthesis. Cross linkage of different polymers at the cell wall increase its rigidity, and confers resistance to physical, chemical and enzymatic breakdown by pathogens [37], [38]. In previous reports, HCAAs: feruloyl-3'-methoxytyramine, feruloyltyramine, and *p*-coumaroyltyramine were detected in cell walls of epidermal onion cells at the sites of *Botrytis allii* penetration [39]. Similarly, serotonin and its hydroxycinnamic acid amides, *p*-coumarylserotonin and feruloylserotonin were accumulated in *Bipolaris oryzae* infected leaves of rice [40]. It is possible that the cell wall thickening also occurs in the cell walls of spikelets, as three hydroxycinnamic acid amides such as *cis*-*p*-coumaroylagmatine, caffeoylserotonin and feruloylserotonin were detected in spikelets of only NIL-R but not in NIL-S.

Deposition of glycosylated and methoxylated flavonoids in rachises was also higher in the resistant than in the susceptible NIL. Flavonoids were also localized to the cell walls of xylem cells and their surrounding cells in NIL-R (Fig.5b). Seven genes encoding different classes of glycosyltransferases and one gene coding for methyltransferase containing protein were identified on the *ctg0954* [36]. These transferases might catalyze the biosynthesis of flavonoid glucosides and methoxyflavonoids. Preformed flavonoids confer durability, longevity, and resistance to the heartwoods of many tree species against wood-rotting fungi [41].

#### Accumulation of phenolic glucosides and altered lignin biosynthesis pathway in wheat

Following pathogen inoculation, phenolic acid glucosides such as β-D-glucopyranosyl-caffeic acid, β-D-glucopyranosyl-sinapic acid and ferulic acid 7-*O*-glucoside were induced in the resistant NIL but not in the susceptible NIL. In transgenic *Populus tremuloides,* down regulation of 4CL increased accumulation of phenolic acid glucosides of *p-*coumaric, ferulic, and sinapic acids and decreased total lignin content, but the syringyl/guaiacyl ratio remained unchanged in the xylem tissue [42]. Likewise, we detected high fold change in sinapoyl alcohol precursor, sinapaldehyde (RRI, FC = 2.2) and sinapoyl alcohol glucoside, syringin (RRI, FC = 1.9) in rachises of resistant NIL, with no significant change in coniferyl alcohol related metabolites that may lead to an increased syringyl/guaiacyl lignin ratio. Monolignols, syringin and coniferin, are stored and transported as phenolic glucosides in plant tissues [43]. A class of UDP-glucosyl transferases [44], along with β-glucosidase, regulate the storage and mobilization of monolignols for lignin biosynthesis [45]. High syringyl/guaiacyl lignin ratio enhanced the resistance to wheat powdery mildew [46]. However, specific role of glycosyltransferases present on *ctg0954* and their substrate specificity in catalyzing formation of phenolic and flavonoid glucosides need to be studied.

### DON Resistance is not a Major Mechanism of FHB Resistance associated with Nyubai Alleles of *Fhb1*

In the present study, neither the total DON produced (TDP) nor did the proportion of DON conversion to D3G (PDC) significantly varied between the resistant and susceptible NILs at 72 hpi, in both spikelets and rachises. Thus, the resistance governed by *Fhb1*, derived from Nyubai, is not due to the DON detoxification by DON-3-*O*-glucosyltransferase. Interestingly, though the NIL-R had significantly less amount of disease than the NIL-S (Fig. 1), the difference was not as drastic as in NIL-R with *Fhb1* derived from Sumai-3, where the symptom was limited mainly to the inoculated spikelets and no bleaching symptom was observed as in Nyubai derived NIL-R [16]. In the NILs derived from Sumai-3, no significant difference in DON level was observed between the inoculated spikelets of susceptible and resistant NILs, however, no DON was detected in un-inoculated spikeletes of NIL-R though was detected in un-inoculated spikelets of NIL-S [16]. DON produced in infected spikelets can move to un-inoculated spikelets [47]. The lack of DON in un-inoculated spikelets of NIL-R may have been due to a higher rate of DON conversion to D3G (PDC) (23). Furthermore, the conversion of DON to D3G was associated with recombinant inbred populations, containing *Fhb1*, of the wheat double haploid lines originating from the cross between Sumai-3 and Thornbird [14]. Other mechanisms reported *in vitro* for DON detoxification is DON-glutathione conjugation [47]. Although, two glutathione S-transferases were significantly induced in NIL-R (Table S3), we did not detect any DON-glutathione conjugates. A comprehensive study, including quantification of DON and D3G as reported here, may prove the mechanisms of resistance in NIL-R derived from Sumai-3.

### Plant Defense Signaling and Oxidative Stress Response

For the first time we report here, the possible role of jasmonic acid signaling in type II FHB resistance in rachises. Along with jasmonic acid, a biologically active form of jasmonate, (+)-7-iso-jasmonoyl-L-isoleucine, was accumulated in greater abundance in NIL-R. (+)-7-iso-jasmonoyl-L-isoleucine is considered to be the bioactive jasmonate encoded by amino acid synthetase (JAR1) [48]. Jasmonic acid elicits several disease resistance related genes involved in biosynthesis of systemin [49], defensin [50], lignin [51] and terpenoid indole alkaloids [52]. We have detected *monoterpenoids*: iridotrial glucoside and loganin, and *indole alkaloids*: 16-epivellosimine and vomilenine which have antimicrobial properties. In wheat, plant defense signaling occurs in a sequential cascade, with Ca^+2^ and salicylic acid signaling are active during early phases of infection, followed by ethylene signaling and jasmonic acid signaling [53]. Jasmonic acid signaling was significant in barley plants inoculated with trichothecene/DON producing but not with non-producing mutant *F. graminearum* isolate [54]. It is possible that in our study in the *F. graminearum* inoculated NIL-R, the DON induces ethylene and jasmonic acid signaling, which in turn activates the biosynthesis of HCAAs. Ethylene non-producing mutants in Arabidopsis were unable to produce hydroxycinnamic acid amides, following inoculation with *Botrytis cinerea* [55]. S-adenosylmethionine synthase, biosynthesized by S-adenosylmethionine synthase, a precursor of ethylene, is a methylgroup donor for the phenylpropanoid pathway, the expression of S-adenosylmethionine synthase was greater in NIL-R than in NIL-S. It is possible that the cell wall thickening observed here may be mainly coded by the ethylene and jasmonic acid signaling leading to the production of HCAAs.

Necrotrophs induce hydrogen peroxide production and kill the host tissue. In response, plants neutralize the reactive oxygen species by counteracting them. Manganese superoxide dismutase, ascorbate peroxidase and glutathione transferase were significantly induced in NIL-R as a general response to pathogen invasion (Table S3). Superoxide dismutase neutralizes the free radicals and further the hydrogen peroxide generated by neutralization will be removed by the catalase or ascorbate peroxidase [56].

In this study, based on non-target metabolomics using LC-hybrid MS, we have provided evidence that the resistance in *Fhb1* derived from Nyubai is not due to the detoxification of the virulence factor DON by the glucosyltransferase to D3G. Instead, we provide strong evidence on the involvement of hydroxycinnamic acid amides, flavonoids and lignin monomers in the formation of cell wall appositions, which play a significant role in restricting the movement of *F. graminearum* in the rachises of NIL with FHB resistance at *Fhb1*.

Integrated non-target metabolomics and proteomics technologies, using LC-hybrid MS, as standardized here, can be applied to elucidate the mechanisms of resistance in more than 100 FHB resistance QTLs identified in wheat and barley. This technology can be adapted to prove the mechanisms of resistance in plants to other biotic stresses. High throughput protocols such as high performance LC (HPLC) [57], Fourier transform infrared spectroscopy (FTIR) [58] and near infrared spectroscopy (NIRS) [59] can be developed to screen for several of the resistance related metabolites as biomarkers for resistance to FHB. Alternatively, specific RRI enzymes identified here can be further explored to enhance plant resistance to FHB.

## Materials and Methods

### Development of Wheat NILs with Contrasting Alleles of *Fhb1*

Resistant and susceptible NILs of wheat, were derived from the mapping population HC374 (resistant)/98B69*L47 (susceptible) by backcross breeding [29]. The FHB resistant parent HC374 was derived from the cross Wuhan/Nyubai which carried FHB resistance QTLs on 3BS, 2DL, 3BSc, 4B and 5AS [60]. The FHB susceptible parent 98B69*L47 was an elite hard red spring wheat accession. BC2F1 plants with 89% recurrent genome and heterozygous between the markers gwm533 and wmc808 which flank *Fhb1* were used in the study. Since, Wuhan is a genetic source of other QTLs, plants were selected for homozygous susceptible alleles at other known FHB resistance loci on other chromosomes 2DL, 3BSc, 4B and 5AS. Microsatellites alleles across the genome of 98B69*L47 were used for recurrent parent genome selection to derive the resistant NIL (NIL-R) and the susceptible NIL (NIL-S). The NILs carried either resistant or susceptible alleles at the *Fhb 1* locus on chromosome 3BS.

### Plant Production, *F. graminearum* Inoculum Production and Inoculation

Wheat NIL-R and NIL-S were grown in greenhouse at 25±3°C with 70±10% relative humidity and 16 h of light & 8 h of darkness. *F. graminearum* (Schwabe) isolate 15–35 (obtained from Dr. S. Rioux, CEROM, Quebec) was maintained on PDA media. For spore production, cultures were grown on rye B agar media, under UV light and darkness, for 16 h and 8 h, respectively, at 25°C. Macroconidia were harvested and the spore count was adjusted to 1×10^5^ macroconidia ml^−1^. Wheat spikelets were point inoculated with 10 µl of spore suspension (Approx. 1000 macroconidia per spikelet) at 50% anthesis, using a syringe with an auto dispenser (GASTIGHT 1750DAD W/S, Hamilton, Reno, NV, USA). For disease severity assessment, a pair of alternative spikelets, approximately at the middle of spike was inoculated. For metabolic/protein profiling, three alternate pairs of spikelets (six spikelets per spike), around the middle of spike, were inoculated. Ten spikes from 6 plants were inoculated for each treatment (pathogen or mock) per replication. The inoculated plants were covered with moistened plastic bags to maintain a saturated atmosphere to facilitate infection, and the bags were removed 48 h post inoculation (hpi).

### Disease Severity Assessment

The number of spikelets diseased was recorded at 3 day intervals until 21 days. From this data, the proportion of spikelets diseased (PSD  =  number of spikelets diseased/total number of spikelets in a spike and area under the disease progress curve (AUDPC) were calculated [18]. A student’s *t*-test was used to compare the AUDPC variation between NILs [61].

### Sample Collection, Metabolites Extraction and LC-hybrid MS Analysis

The inoculated spikes were harvested at 72 hpi. The spike was trimmed on both the ends, six inoculated spikelets and rachis harvested separately were immediately frozen in liquid nitrogen and stored at −80°C until use. The rachis and spikelet samples were ground in liquid nitrogen. Metabolites were extracted in 60% ice-cold aqueous methanol as standardized in our lab and analysed using liquid chromatography coupled with hybrid mass spectrometers (LC-ESI-LTQ-Orbitrap, Thermo Fisher, Waltham, MA), fitted with a relatively polar reverse phase C18 Kinetex column (Phenomenex, CA, USA) [20]. Mass resolution was set to 60 000 (FWHM) at 400 *m/z*. MS1 data were recorded in centroid mode. For compound identification, one sample each from four treatment combinations (RP-rachis, RM-rachis, RP-spikelets and RM-spikelets; where RP = resistant NIL with pathogen inoculation, RM =  resistant NIL with mock inoculation, SP = Susceptible NIL with pathogen inoculation, SM =  susceptible NIL with mock inoculation) were rerun to obtain MS/MS fragmentation at normalized collision energy of 35 eV.

### Experimental Design

The experiment was conducted in a greenhouse as a randomized complete block design with two NILs (NIL-R & NIL-S) and two inoculations (pathogen and mock) making four treatment combinations; RP, RM, SP and SM. Treatments were replicated five times over time, at three day intervals. Each sample or the experimental unit consisted of about 60 spikelets or ten rachises that were collected from ten spikes per replication.

### LC-hybrid MS Data Processing using XCMS

The output from the LC-hybrid MS was imported to XCMS bioinformatics tool on R platform for peak detection, matching, grouping and retention time alignment of peaks across the samples. For feature (*m/z* and retention time) detection, *centWave* alogorithm with mass deviation (µ)  = 3ppm, peak width (range wmin - wmax) of 10–30, and signal to noise (*S/N*) ratio of 5 was employed [62]. XCMS processing was carried out independently for rachis and spikelets, in pair wise treatment combinations (RP vs RM, RM vs SM, SP vs SM and RP vs SP). The adducts, isotopes and neutral losses were identified using CAMERA algorithm based on peak annotation [63]. Following this, the data from all four treatment combinations were combined using MetaXCMS [64], separately for rachis and spikelets. The accurate mass and their abundance (relative intensity) were imported to MS Excel; peaks that were not consistent among replicates and those annotated as isotopes, adducts, dimers and neutral losses were excluded from further analyses.

### Statistical Analysis and Identification of Resistance Related (RR) Metabolites

The data on intensity of peaks of monoisotopic masses (*m/z*  =  mass/charge ratio, subtracted with a proton mass because of negative ionization) were subjected to pair-wise student’s *t*-test analysis, using SAS [61]. The treatment combinations tested were RM vs SM, RP vs RM and SP vs SM and the peaks significant at *P*<0.05 were retained. A data dimension reduction technique, canonical discriminant analysis, was applied to classify the treatment effects based on metabolites [19]. The abundances of 672 and 693 metabolites common to all treatments from rachises and spikelets, respectively, were subjected to canonical discriminant analysis to classify the observations. The Can vectors were used to identify the resistance functions, by correlating the observed clusters to resistance phenotypes. The treatment significant metabolites were also used to identify resistance related (RR) metabolites. The metabolites with significantly higher abundances in NIL-R than in NIL-S were considered as RR metabolites. These were further grouped into RR constitutive (RRC  =  RM > SM) and RR induced metabolites (RRI  =  (RP> RM) > (SP>SM)). For these RR metabolites, the fold change (FC) in abundance relative to susceptible (NIL-R/NIL-S) was calculated. When a metabolite was induced only in the NIL-R (PRr  =  pathogenesis related) and not in the NIL-S (PRs), then the fold change was considered infinity. For such metabolites, only the fold change in PRr metabolite (RP/RM) was reported. The RR metabolites were putatively identified based on three criteria: i) accurate mass match (accurate mass error (AME) of <5ppm) with metabolites reported in different databases: METLIN, KNApSAcK, Plant Metabolic Network (PMN), LIPIDMAPS, KEGG and McGill-MD [65]; ii) fragmentation pattern match with those in databases and also those from in-house spiked standards (Fig. S1) [66]; iii) *in silico* fragmentation verification using Massspec scissors in Chemsketch (ACD labs, Toronto) [67](Fig. S2). The metabolites were mapped on metabolic pathways using pathway tool omics viewer [68] searched against *Arabidopsis thaliana* and *Populus trichocarpa* metabolites. The concentration of DON, 3ADON and D3G were calculated based on standard curves previously developed [22]. Total DON produced (TDP) was calculated by summing up the quantity of DON and D3G, and the proportion of TDP converted to D3G based on the ratio, PDC  =  D3G/TDP.

### Histochemical Staining of Hydroxycinnamic Acid Amides (HCAAs) and Flavonoids

Rachises of wheat NILs, ten each of pathogen and mock inoculated spikes, were harvested at 72 hpi. Rachis nodes of inoculated pairs of spikelets along with one internode above were cut using a scalpel and immediately frozen at −20°C. For cryo-sectioning, tissues were embedded in Shandon CRYOMATRIX (Richard-Allan Scientific, Kalamazoo, MI) just prior to sectioning at −25°C. Thin, 10 µm cross sections were cut using cryotome (Leica, CM1850, Concord, Ontario ) and collected on glass slides. Sections were washed with distilled water for 2 min, stained with Neu’s reagent (1% 2-amino ethyl diphenyl borinate (Sigma Aldrich) in absolute methanol) for 5 min and mounted in 15% glycerol [69]. The cross sections of ten rachis for each treatment, with at least five sections from each rachis, were observed under confocal microscope (Nikon, Eclipse E800, USA) for the chemifluorescence. Fluorescence of HCAAs was observed with blue laser diode excitation at 405 nm fitted with emission filter HQ442/45. Fluorescence of flavonoids was observed with Argon excitation filter (488 nm) and emission filter HQ 515/30.

**Table 5:** Primer sequences used for studying transcript expression in wheat near isogenic lines with resistant and susceptible alleles of *Fhb1* using quantitative real-time PCR.

GENBANK ID	Gene name	Forward primer	Reverse primer
AY234333.1	*TaACT*	CATCCTGCTACCGTCCTTC	GGAGCTAGTCGAGGGTGTAG
AB181991.1	Actin	CCGGCATTGTCCACATGAA	CCAAAAGGAAAAGCTGAACCG

### Protein Extraction and Shotgun Proteomic Analysis

The residual tissue after metabolite extraction from pathogen and mock inoculated rachises was used for protein extraction. Total protein was extracted using plant total protein extraction kit (PE0330, Sigma Aldrich, USA) supplied with protease inhibitor. Two milligrams of protein was digested in trypsin solution (6 ng/µl) (Promega, QC, Canada) at 58°C for 1 h and peptides were extracted using the extraction buffer (1% formic acid/50% ACN) and dried using vacuum centrifuge.

### LC-hybrid MS Analysis of Tryptic Peptides

The peptide extracts were re-solubilized 0.2% formic acid and analysed using LC-ESI-LTQ-Orbitrap (Thermo Fisher, Waltham, MA), fitted with C18 Jupiter column (Phenomenex, CA, USA), installed on the nanoLC-2D system (Eksigent, Florida, USA) [70]. LC-hybrid MS data acquisition was accomplished using a four scan event cycles comprised of a full scan MS for scan event 1 acquired in the Orbitrap. The mass resolution for MS was set to 30,000 (at *m/z* 400) and used to trigger the three additional MS/MS events acquired in parallel in the linear ion trap for the top three most intense ions. The full MS scan range was divided into 2 smaller scan ranges (300–700 and 700–2000 Da) to improve dynamic range. The data dependent scan events used a maximum ion fill time of 100 ms and 1 microscan to increase the duty cycle for ion detection. Target ions already selected for MS/MS were dynamically excluded for 15 s.

### Protein Identification and Quantification

The data output from LC-hybrid MS on MS/MS were analyzed using MASCOT (Matrix Science, London, UK; version 2.2.04). MASCOT was set up to search the nr_20101214 database (selected for Viridiplantae, 848476 entries as on 12 March 2012). Trypsin was used as the enzyme allowing for up to 2 missed cleavages. The mass tolerances for precursor ion and fragment ions were set to 15 ppm and 0.6 Da, respectively. Carbamidomethyl and oxidation of methionine were allowed as variable modifications. Scaffold (version Scaffold 3.3.1, Proteome Software Inc., Portland, OR) was used to validate MS/MS based peptide and protein identifications. Peptide identifications were accepted if they exceeded MASCOT threshold level of 20. Proteins that contained similar peptides and could not be differentiated based on MS/MS analysis alone were grouped to satisfy the principles of parsimony. Peptide and proteins were identified with >95.0% probability and proteins identified with at least 2 identified peptides were retained.

Normalized spectral abundance factor (NSAF) [71] was used for the relative quantification and identification of resistance related induced (RRI =  (RP>RM)> (SP>SM)) proteins, using student’s *t* test. Three biological replicates were used for the analysis and each biological replicate consisted of 10 spikes collected from 5 different plants. Blast2GO was used to assign gene ontology (GO) terms and mapping on KEGG pathways. Proteins were also searched in AgBase (Mississippi State University) and Plant protein database (PPDB, http://ppdb.tc.cornell.edu/) for GO association.

### Quantitative Real-time PCR

Total RNA was extracted from five biological replicates (10 rachis collected from 5 plants for each replicate) using RNeasy Plant mini kit (Quiagen) and treated with DNase I (Quiagen). Purified RNA (500 ng from each sample) was reverse transcribed using iScript cDNA synthesis kit (BioRad, ON, Canada). Two microliters of 40x-diluted cDNA was used in a quantitative real-time PCR (qPCR) reaction using iQ SYBR Green Supermix (BioRad) in an CFX384TM Real-Time System (BioRad, ON, Canada). Dilution series were used to determine the linear amplification range and relative quantification and mRNA abundance was normalized to Actin. Primer sequences used for actin and agmatine coumaroyl transferase are given in Table 5.

## Supporting Information

Figure S1 MS/MS spectra of spiked standards
(PDF)

Figure S2 MS/MS spectra of metabolites detected in wheat NILs and in silico fragments verification of metabolites.
(PDF)

Figure S3 Resistant related induced (RRI) proteins in wheat NIL with resistant Fhb1allele following F. graminearum inoculation.
(PDF)

Figure S4 Satellite metabolic pathways of wheat-Fusarium interaction.
(TIF)

Table S1 Fusarium head blight resistance related metabolites identified in rachises of wheat NIL with resistant Fhb1 allele following F. graminearum or mock inoculation.
(DOC)

Table S2 Fusarium head blight resistance related metabolites identified in spikelets of wheat NIL with resistant Fhb1 allele following F. graminearum or mock inoculation.
(DOC)

Table S3 Resistant related induced (RRI) proteins in wheat NIL with resistant Fhb1 allele following F. graminearum inoculation.
(DOC)
