# Supplementary material for: Integrated Metabolo-Proteomic Approach to Decipher the Mechanisms by Which Wheat QTL (Fhb1) Contributes to Resistance against Fusarium graminearum
Source: PLoS One. 2012 Jul 12;7(7):e40695. doi: 10.1371/journal.pone.0040695 (PMC3398977; doi:10.1371/journal.pone.0040695)
Supplement: Figure S1 — MS/MS spectra of spiked standards (PDF) [file pone.0040695.s001.pdf]

**Fig. S1.** MS/MS spectra of spiked standards:
(a) Deoxynivalenol,
(b) Deoxynivalenol-3-O-glucoside,
(c) 3 Acetyl dexynivalenol,
(d) t-cinnamic acid,
(e) L-Phenylalanine,
(f) Coniferyl aldehyde,
(g) Sinapaldehyde,
(h) Sinapic acid,
(i) Jasmonate,
(j) Linolenate
(k) Linoleate

## (a) Deoxynivalenol

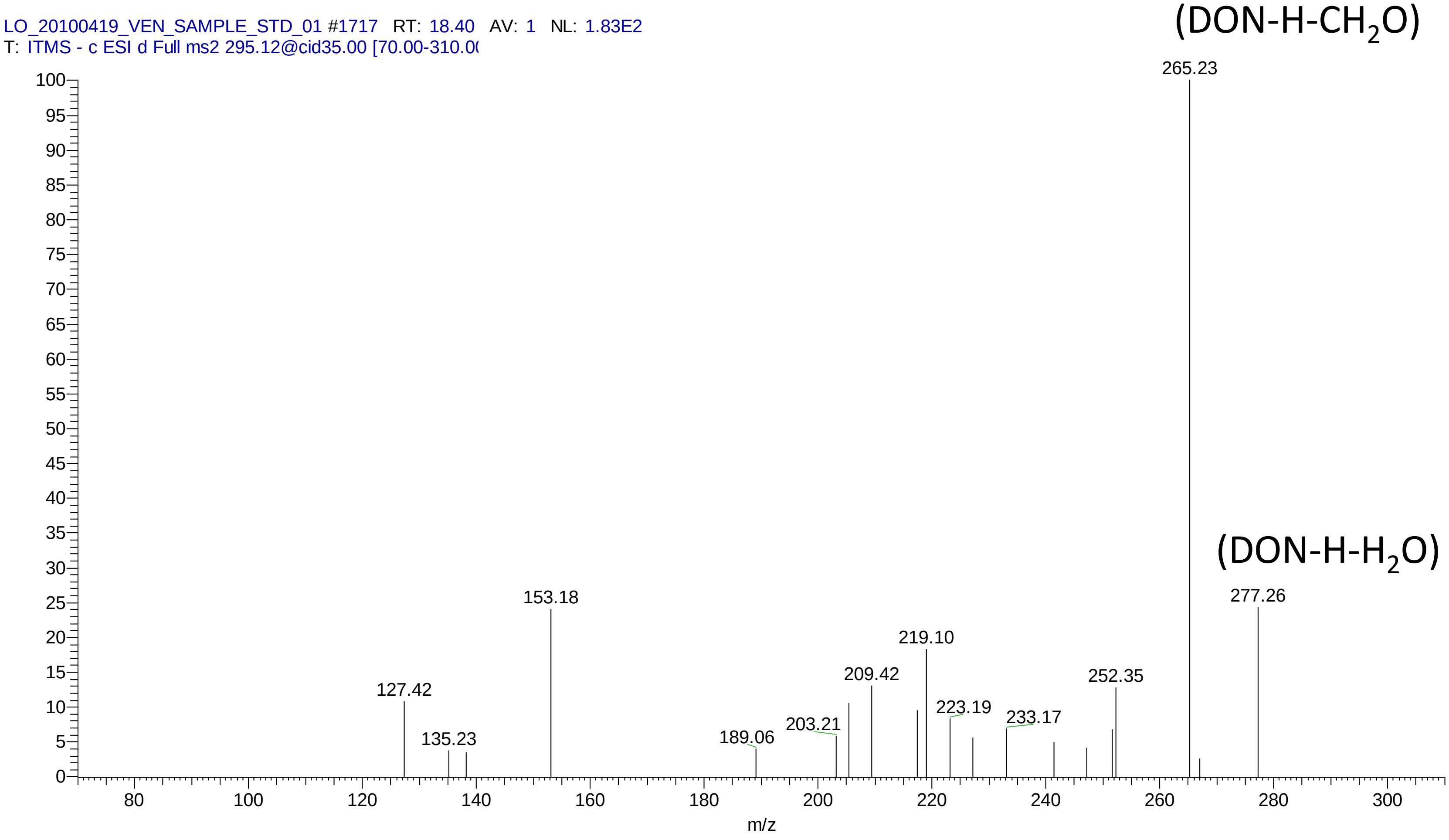

## (b) Deoxynivalenol 3-*o*-glucoside (D3G)

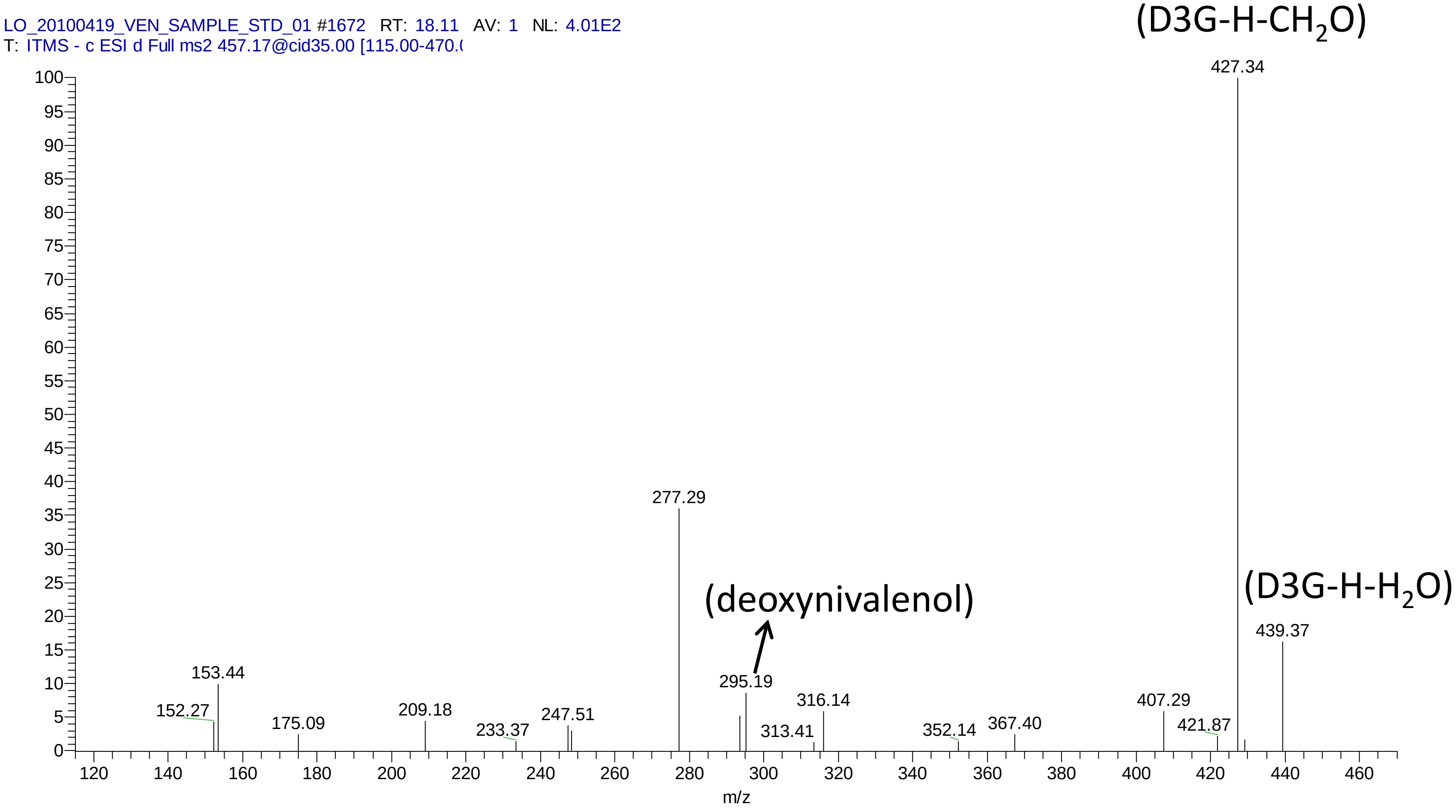

# (c) 3-Acetyl deoxynivalenol (3ADON)

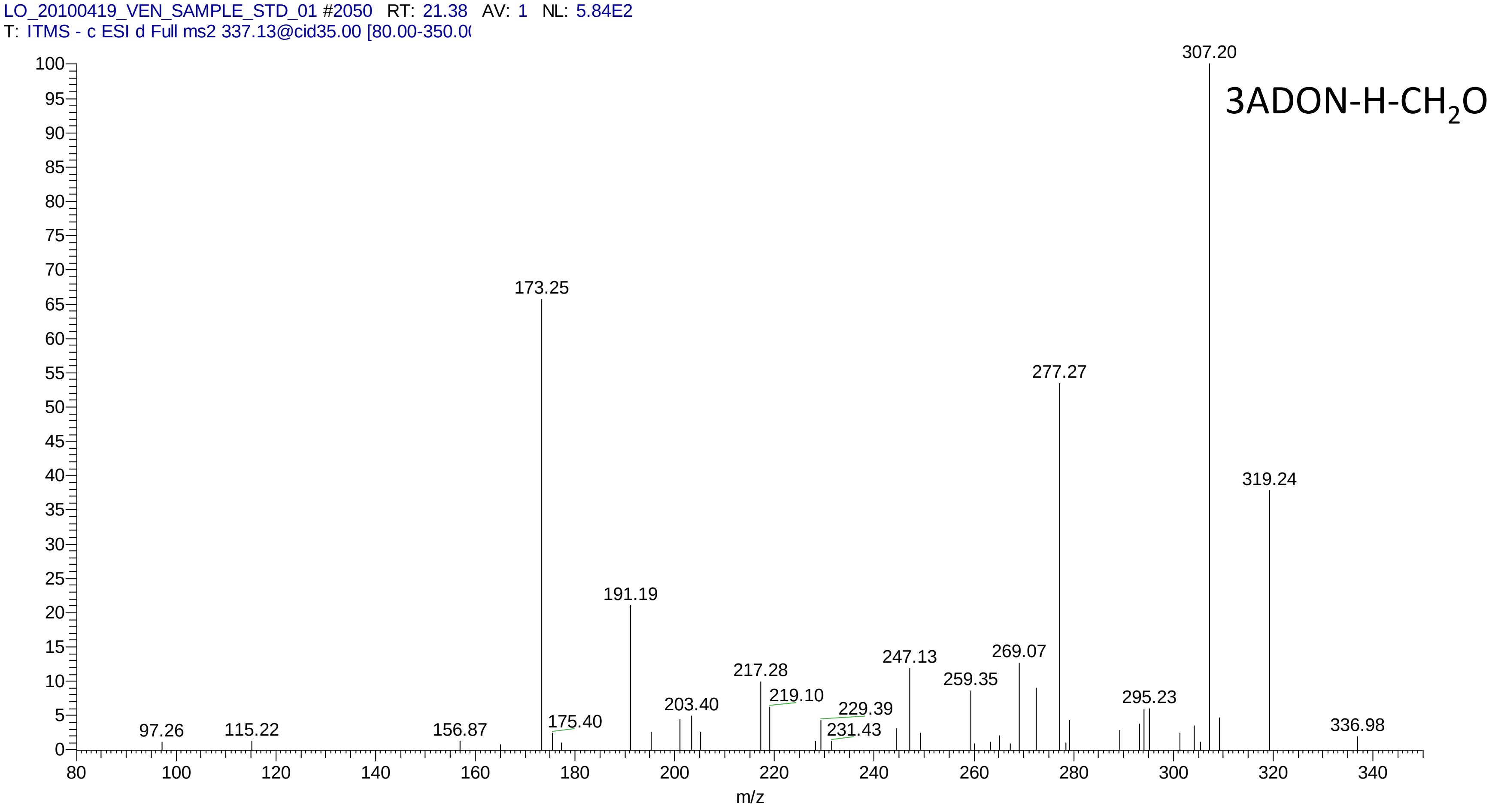

## (d) *t*-Cinnamic acid

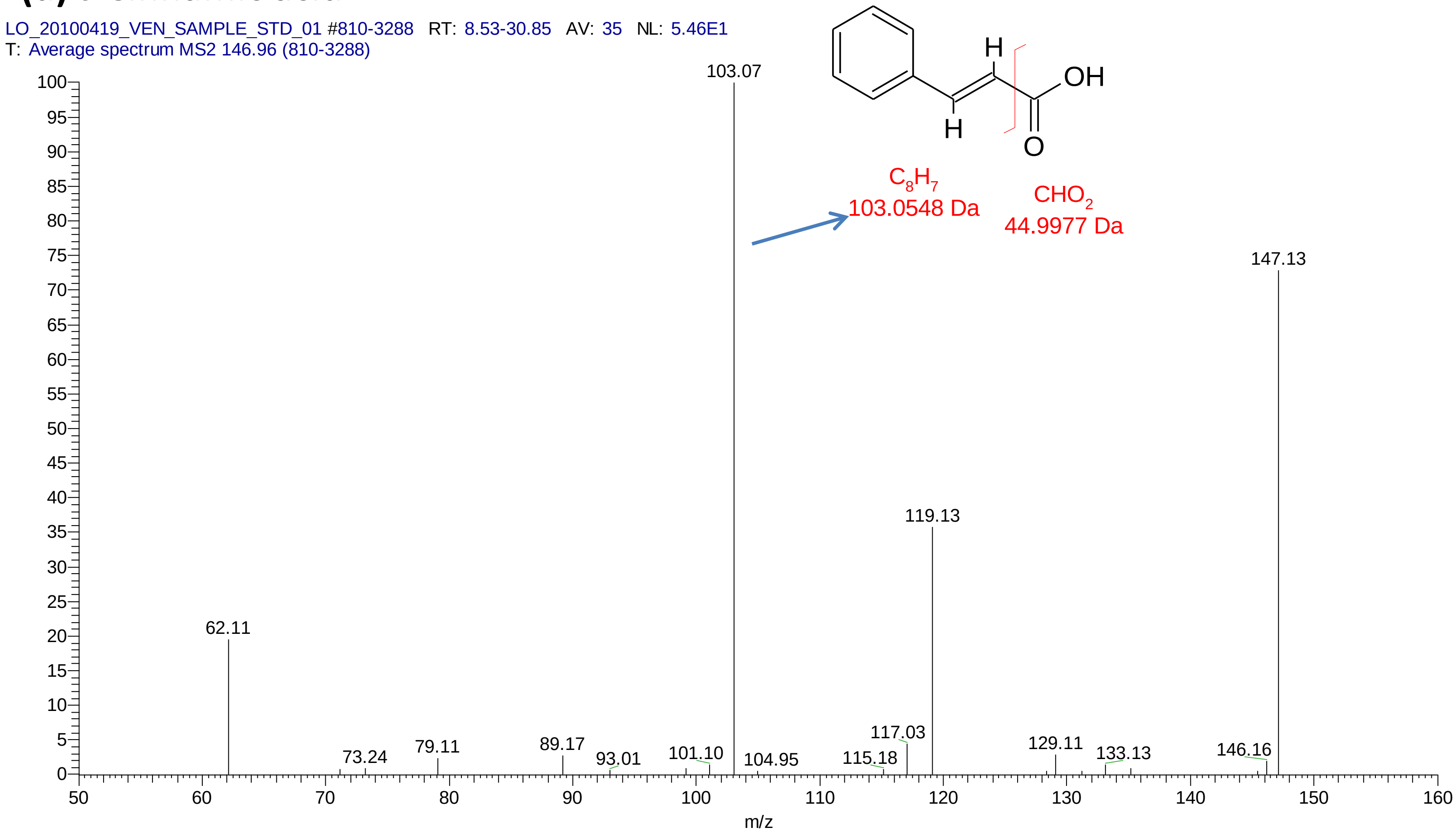

## (e) L-Phenylalanine

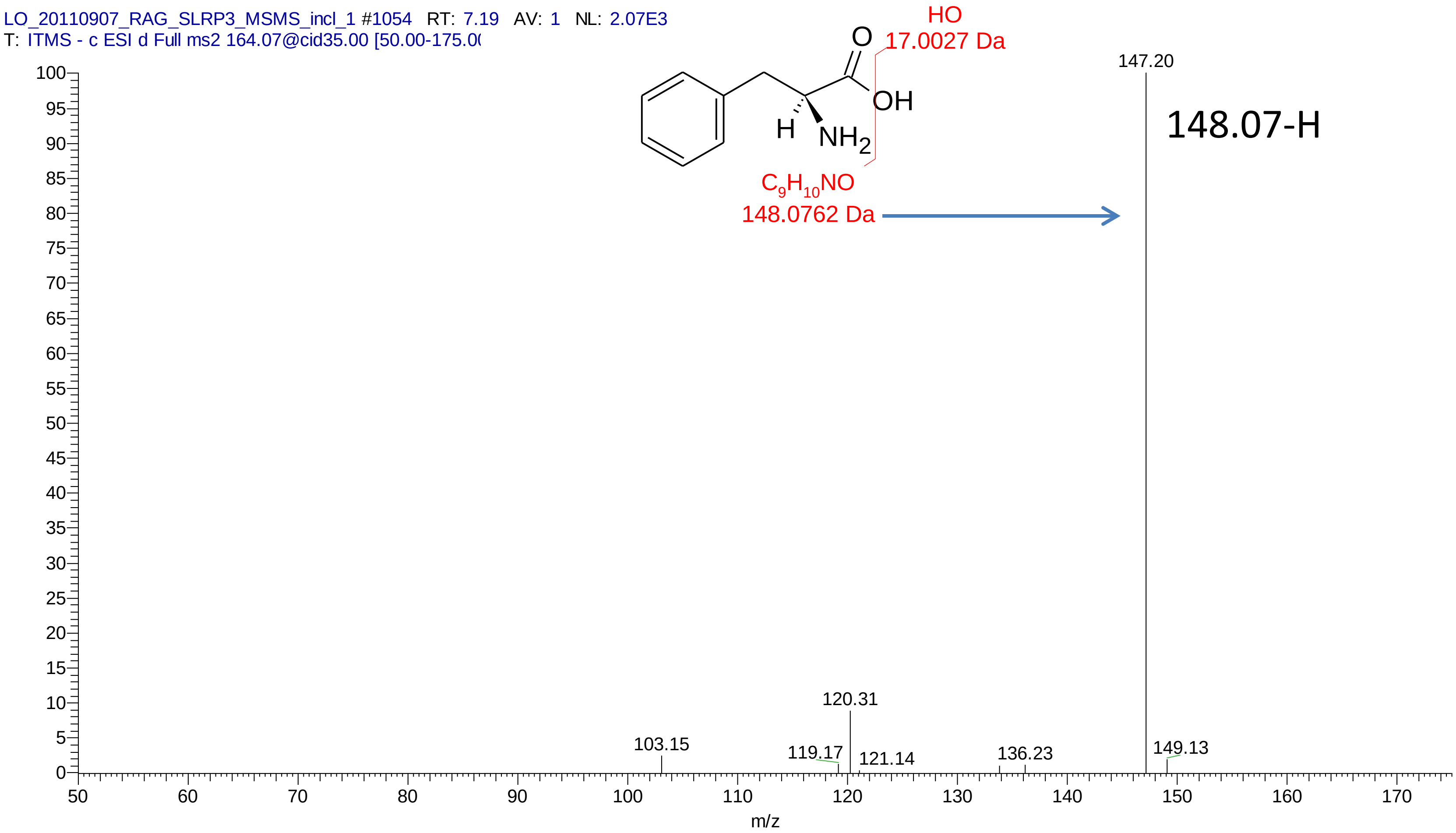

# (f) Coniferyl aldehyde

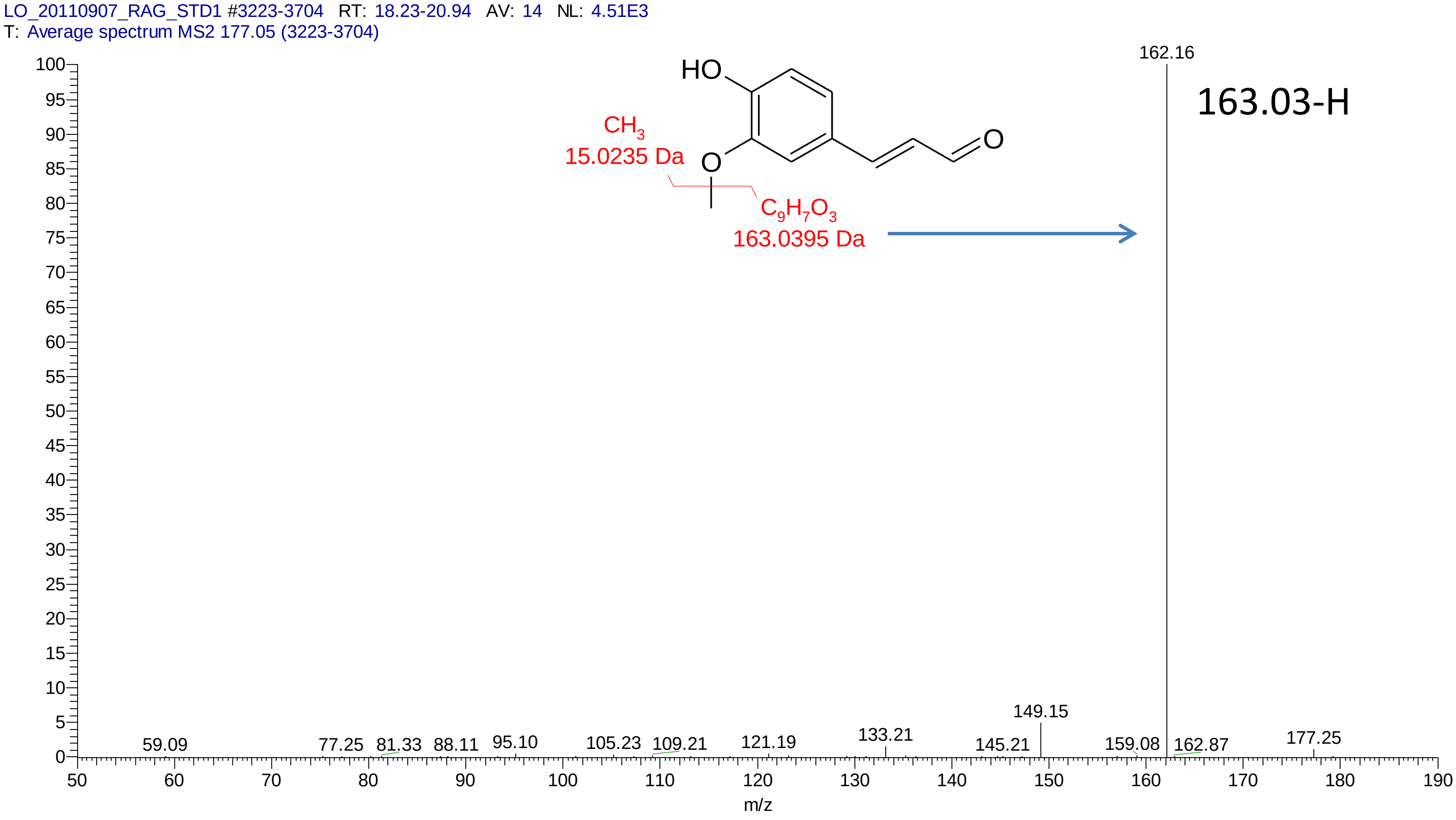

# (g) Sinapaldehyde

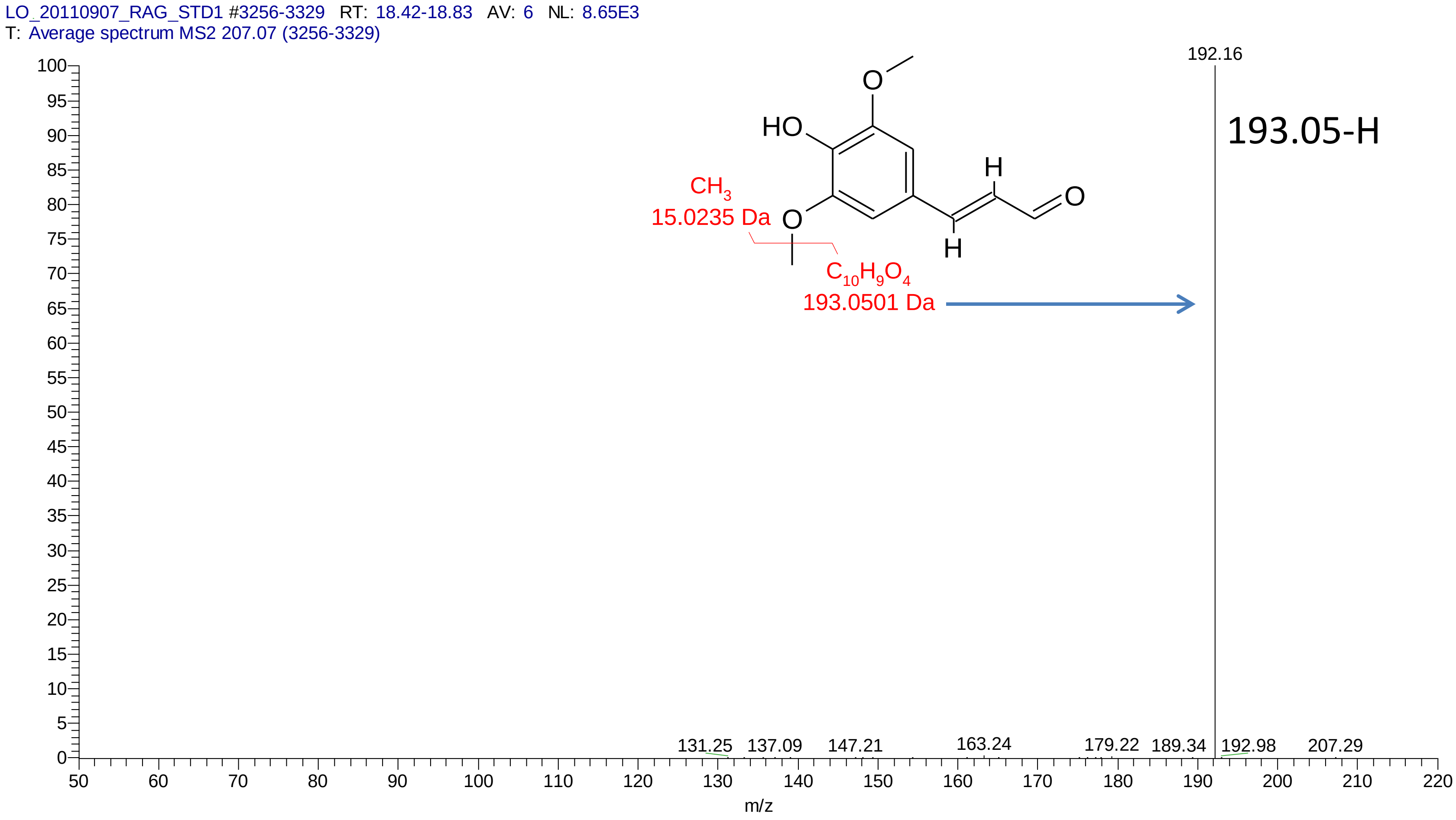

## (h) Sinapic acid

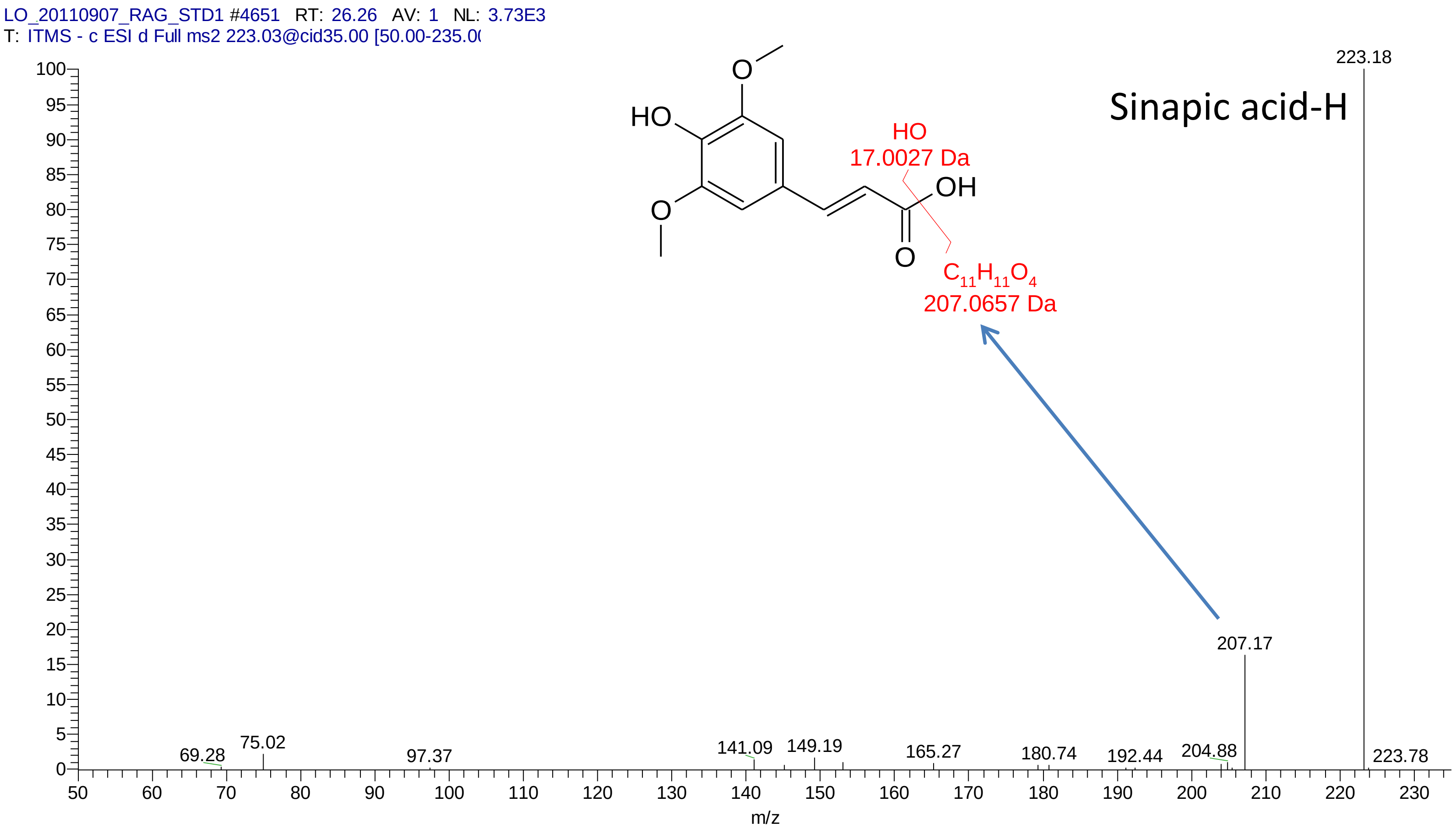

## (i) Jasmonate

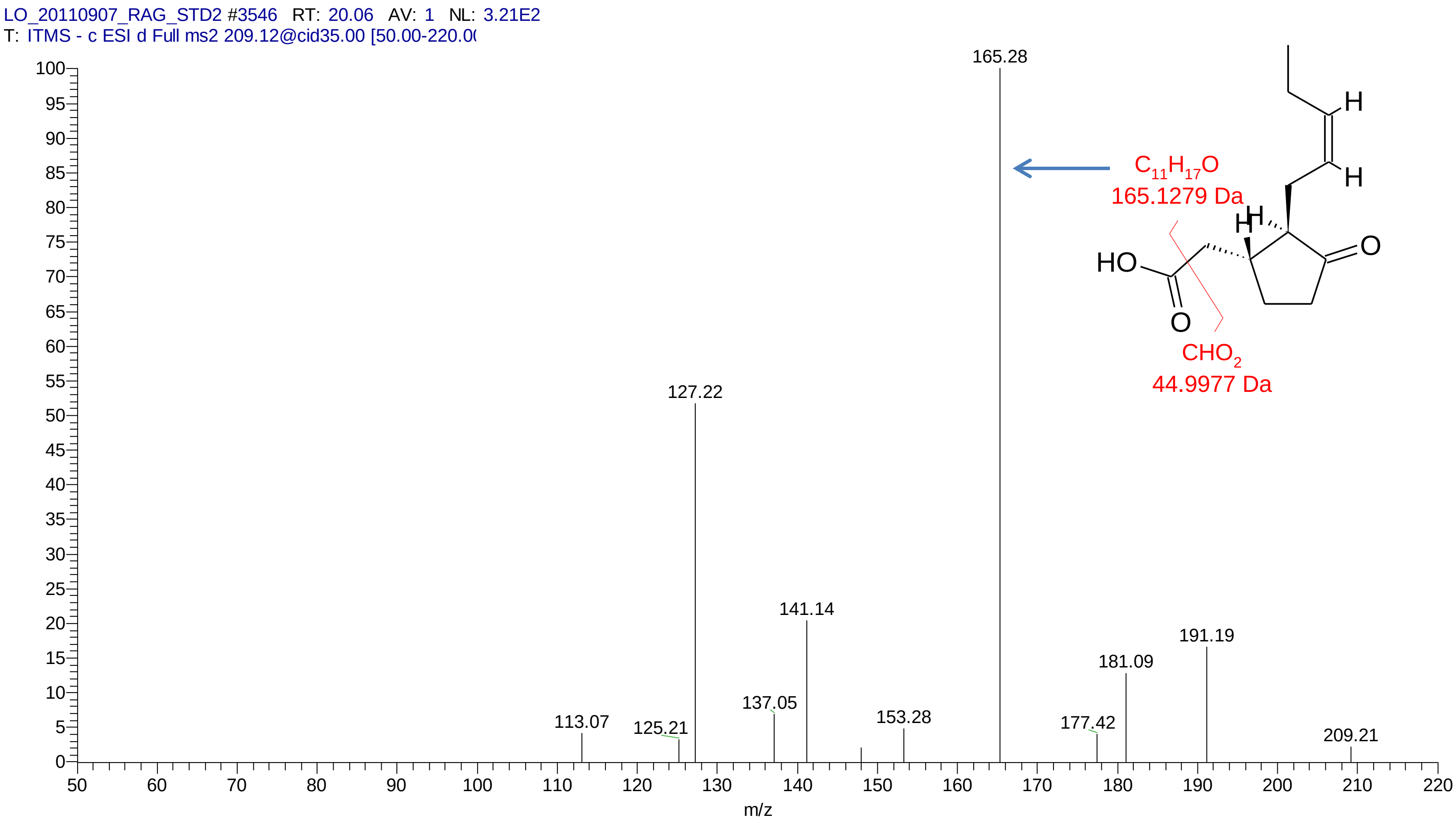
LO_20110907_RAG_STD2 #3546 RT: 20.06 AV: 1 NL: 3.21E2
T: ITMS - c ESI d Full ms2 209.12@cid35.00 [50.00-220.0(
165.28
127.22
141.14
191.19
181.09
113.07
125.21
137.05
153.28
177.42
209.21
m/z
HO
O
O
H
H
H
H
C11H17O
165.1279 Da
CHO2
44.9977 Da

# (j) Linolenate

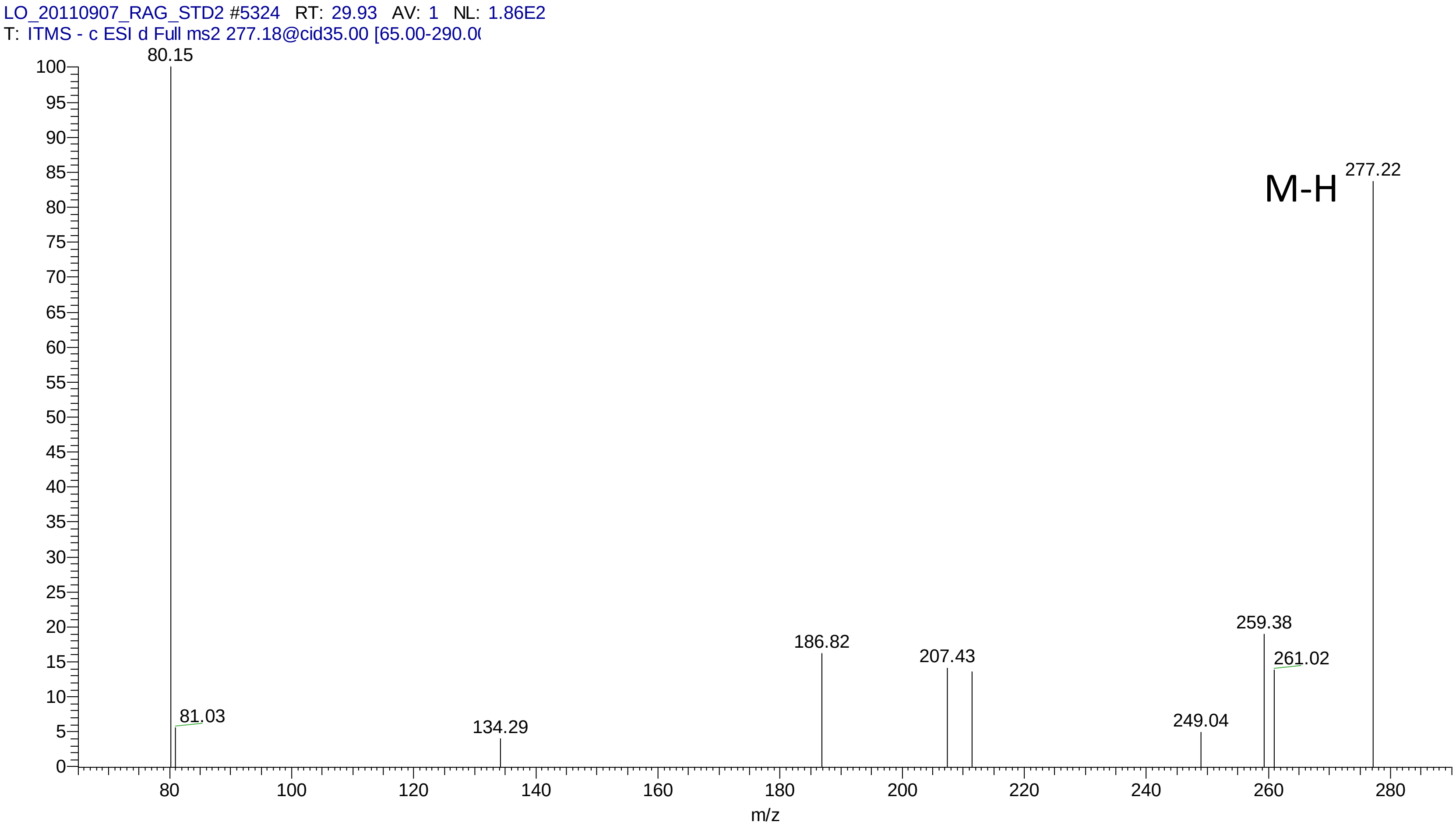

## (k) Linoleate

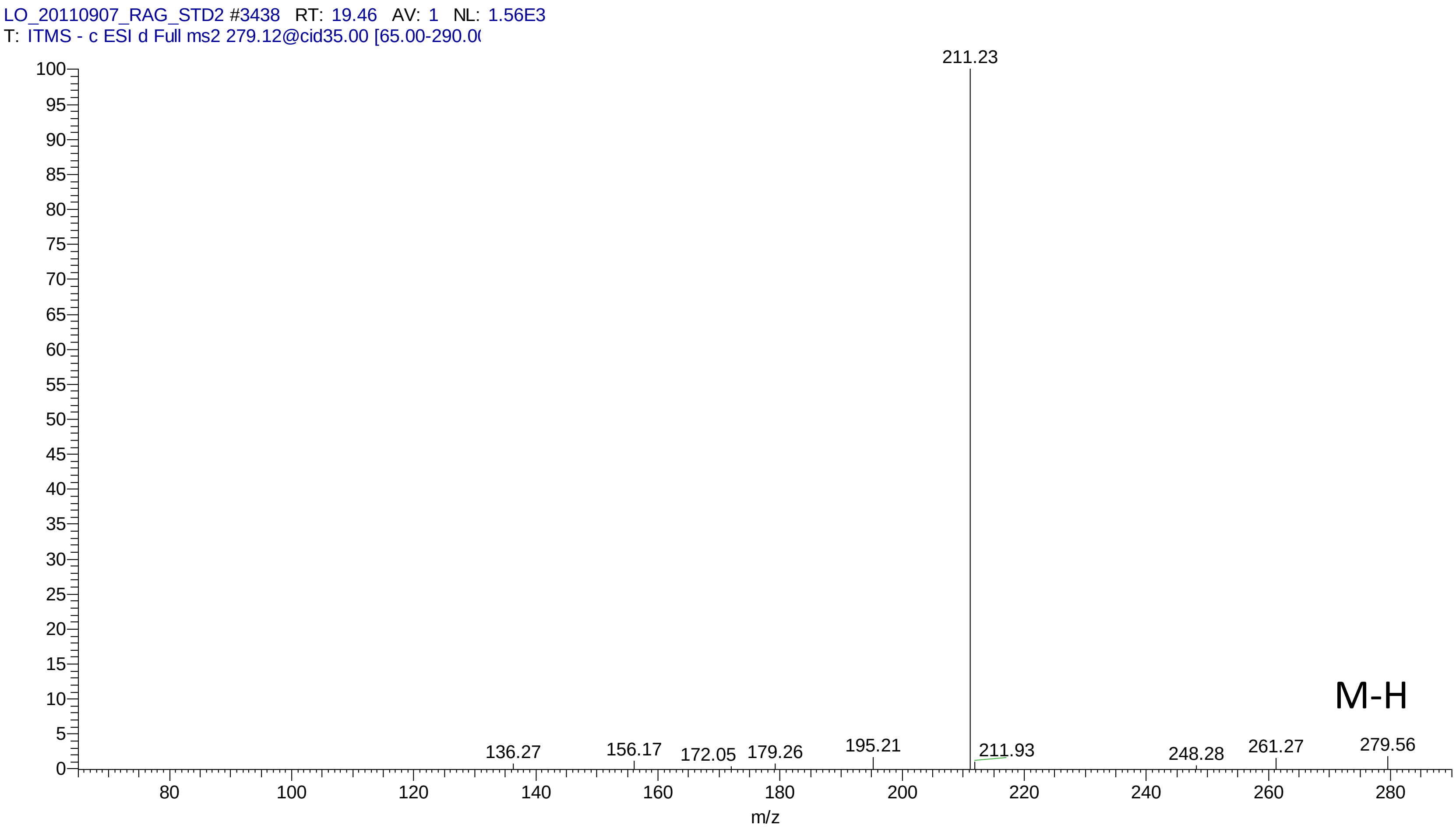

LO_20110907_RAG_STD2 #3438 RT: 19.46 AV: 1 NL: 1.56E3
T: ITMS - c ESI d Full ms2 279.12@cid35.00 [65.00-290.0(
211.23
136.27
156.17
172.05
179.26
195.21
211.93
248.28
261.27
279.56
M-H
m/z
