# Supplementary material for: Integrated Metabolo-Proteomic Approach to Decipher the Mechanisms by Which Wheat QTL (Fhb1) Contributes to Resistance against Fusarium graminearum
Source: PLoS One. 2012 Jul 12;7(7):e40695. doi: 10.1371/journal.pone.0040695 (PMC3398977; doi:10.1371/journal.pone.0040695)

Fig. S2. MS/MS spectra and *In silico* fragmentation of metabolites detected in wheat NILs with contrasting alleles of *Fhb1* following *F. graminearum* or mock inoculation. Databases used for metabolite identification are given in Table S1 and S2:

(a) p-Coumaroylputrescine,
(b) Feruloylputrescine,
(c) Cinnamoyltyramine,
(d) cis-p-Coumaroylagmatine,
(e) Feruloylagmatine,
(f) Cinnamoylserotonin,
(g) Caffeylserotonin,
(h) Feruloylserotonin,
(i) Caffeyl alcohol,
(j) 4-coumaroylshikimate,
(k) 4-coumaroylquinate,
(l) β-D-glucopyranosyl-caffeic acid,
(m) Ferulic acid 7-O-glucoside,
(n) β-D-glucopyranosyl-sinapic acid,
(o) Salicylic acid 2-O-β-D-glucoside,
(p) Jasmonoyl-L-isoleucine ,
(q) Abscisic aldehyde

(a) *p*-Coumaroylputrescine

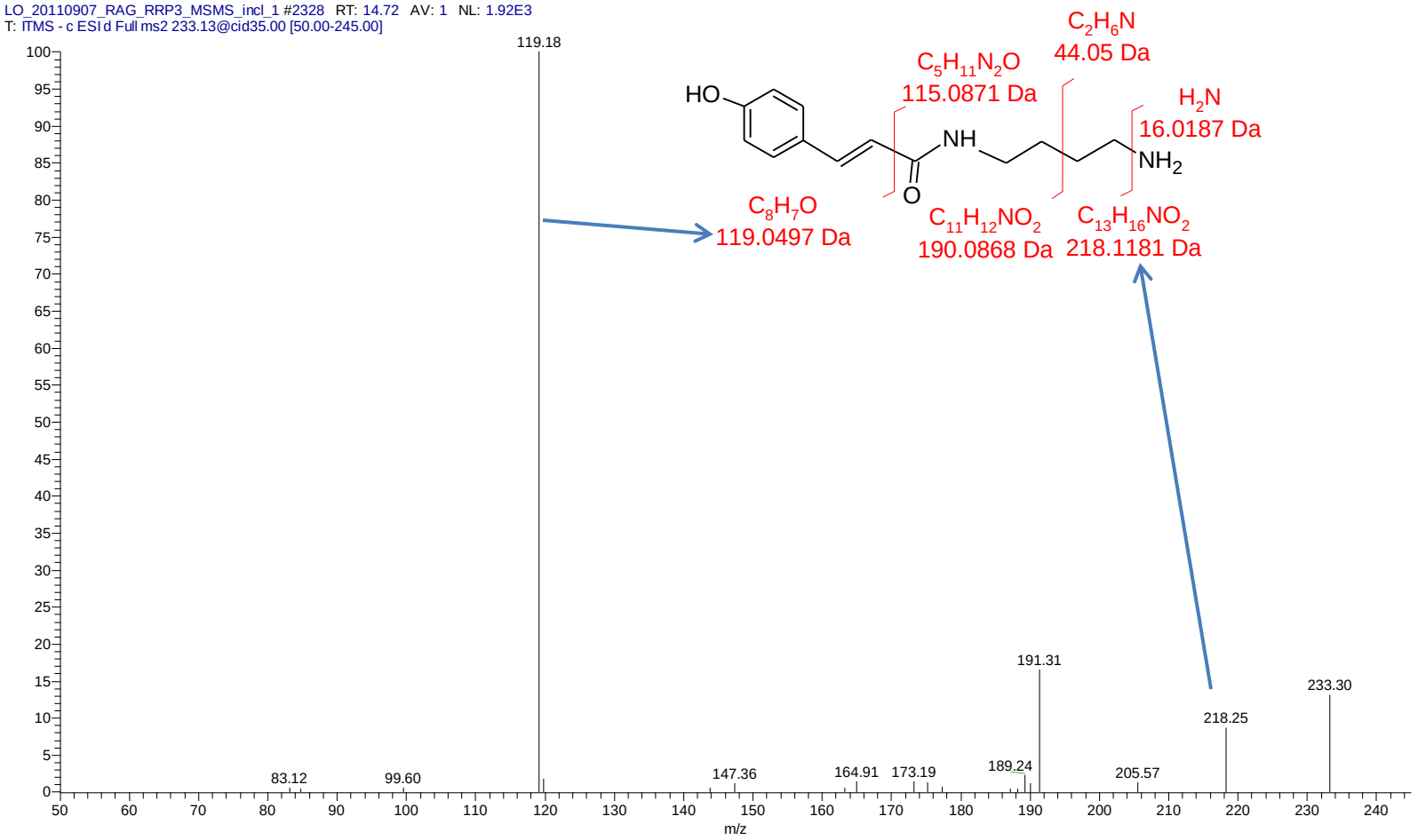

(b) Feruloylputrescine

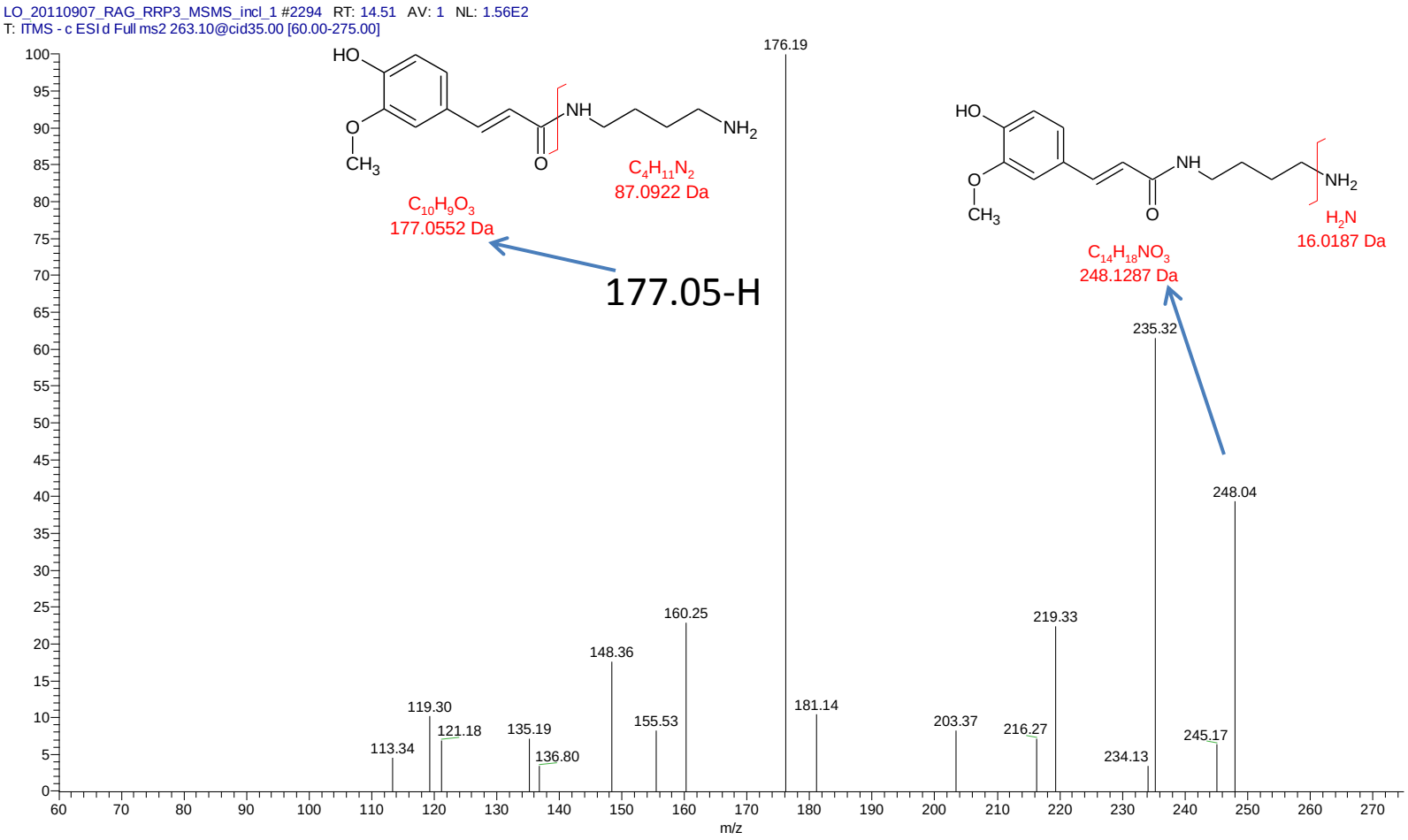

(c) Cinnamoyltyramine

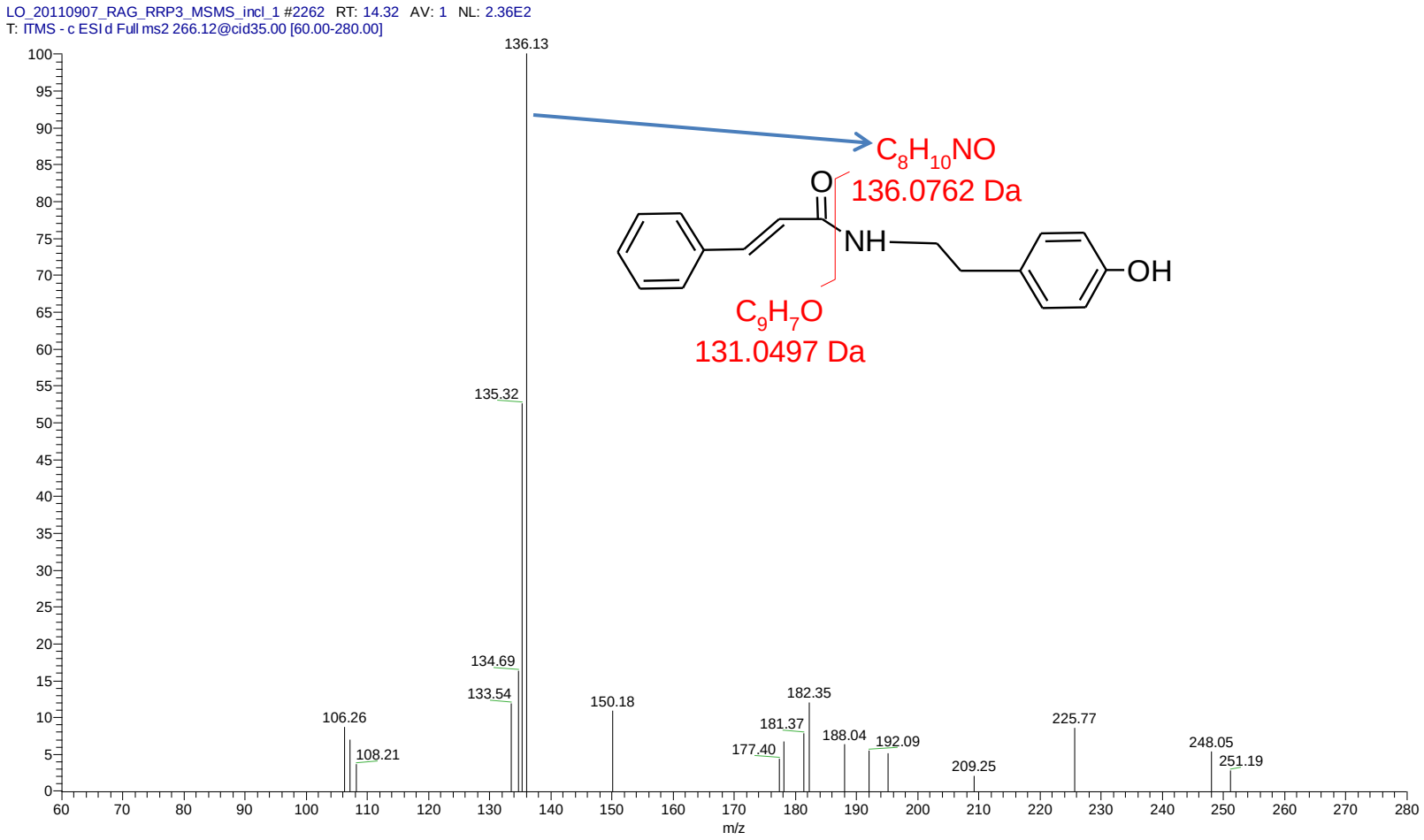

(d) *cis*-*p*-Coumaroylagmatine

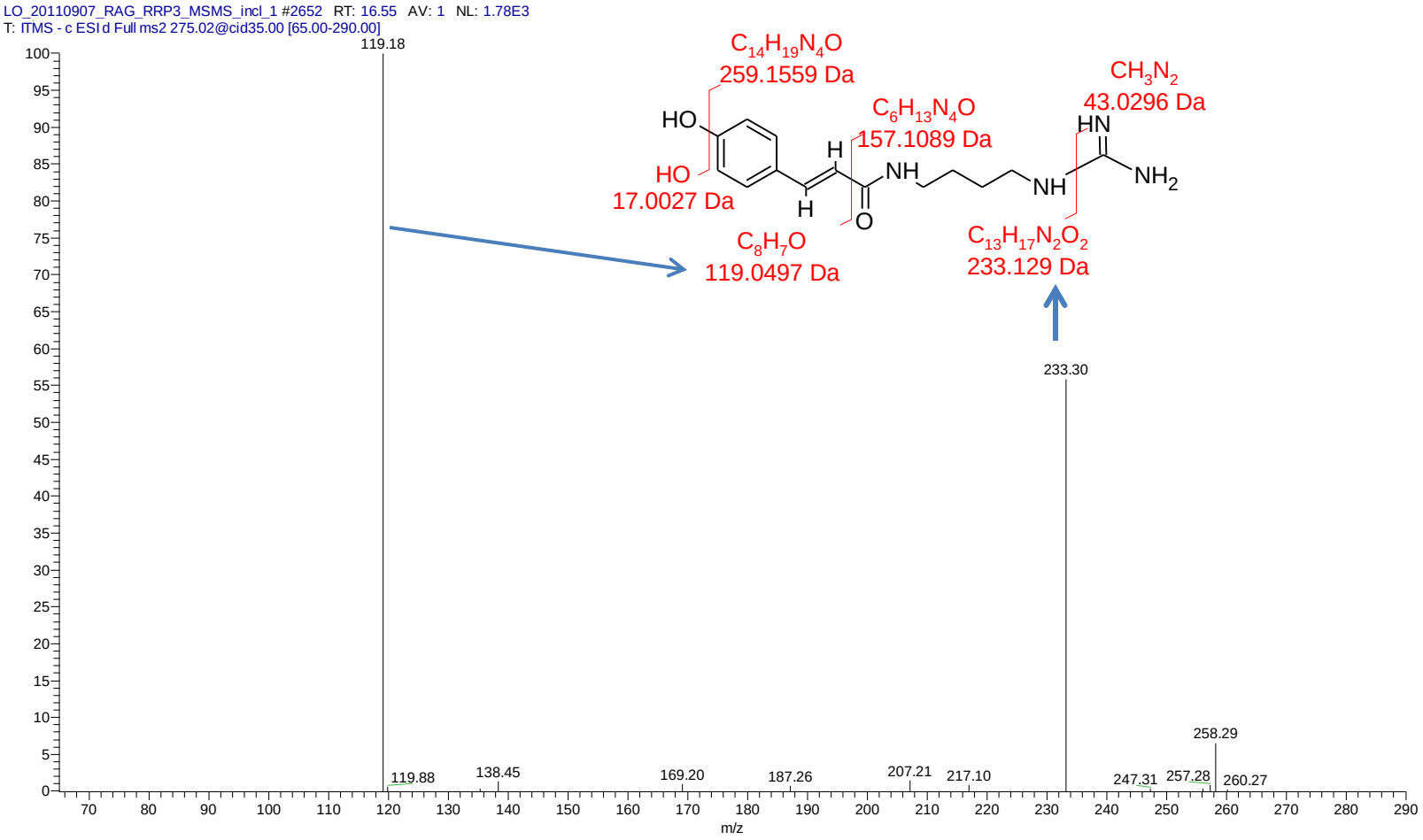

(e) Feruloylagmatine

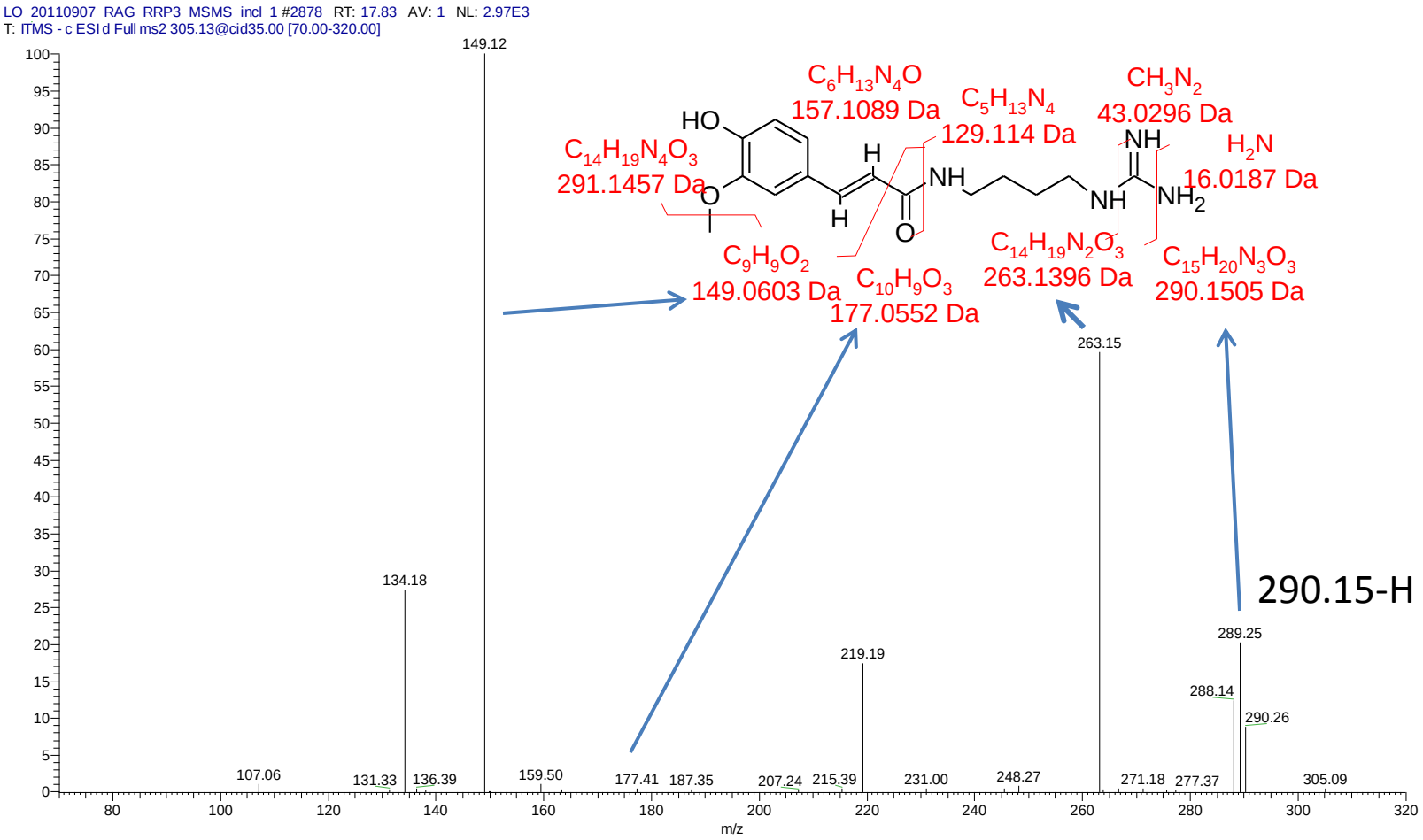

(f) Cinnamoylserotonin

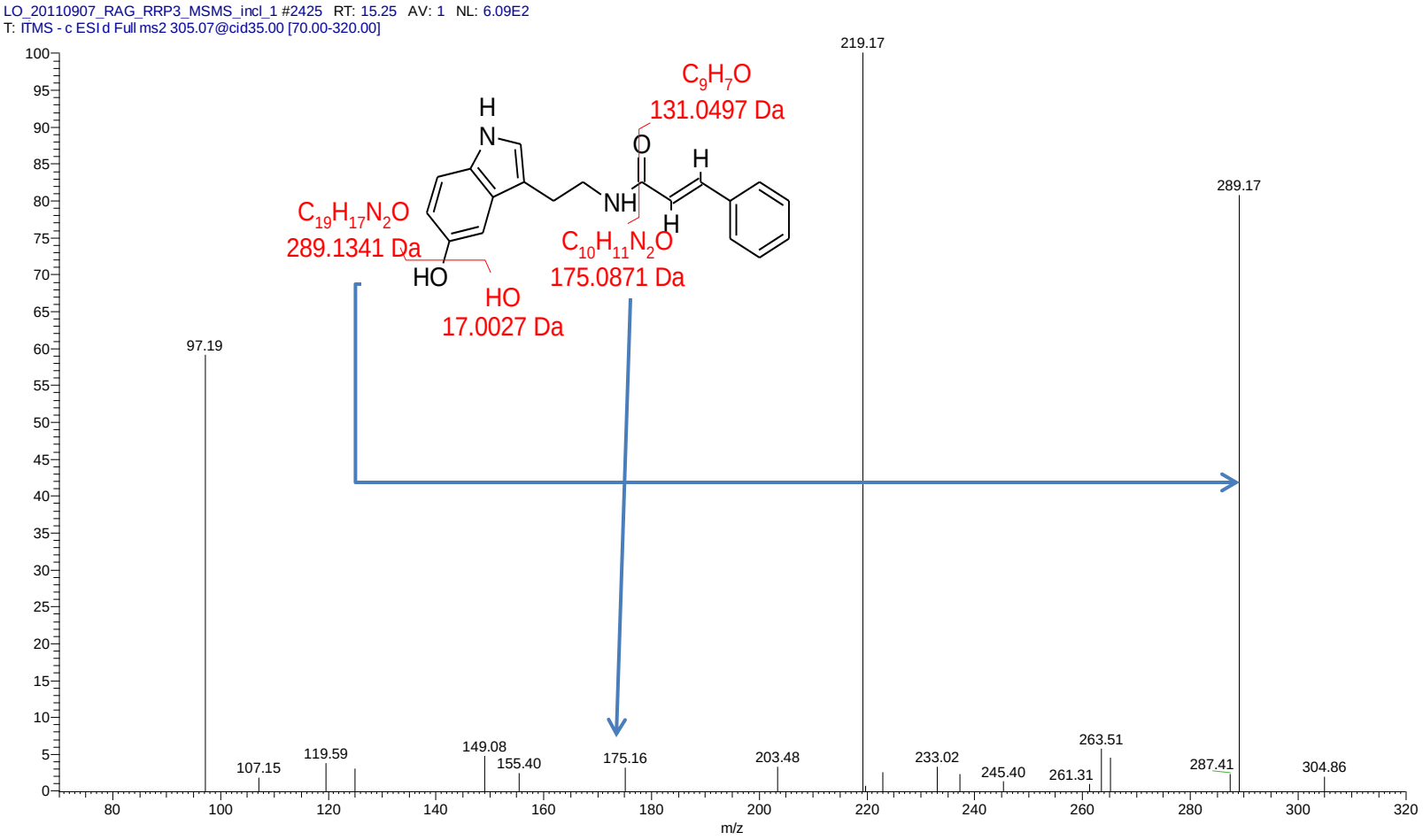

(g) Caffeylserotonin

LO_20110907_RAG_RRP3_MSMS_incl_2 #1633 RT: 16.46 AV: 1 NL: 2.71E1
T: ITMS - c ESI d Full ms2 337.12@cid35.00 [80.00-350.00]

$C_9H_7O_3$
163.0395 Da

$C_{10}H_{11}N_2O$
175.0871 Da

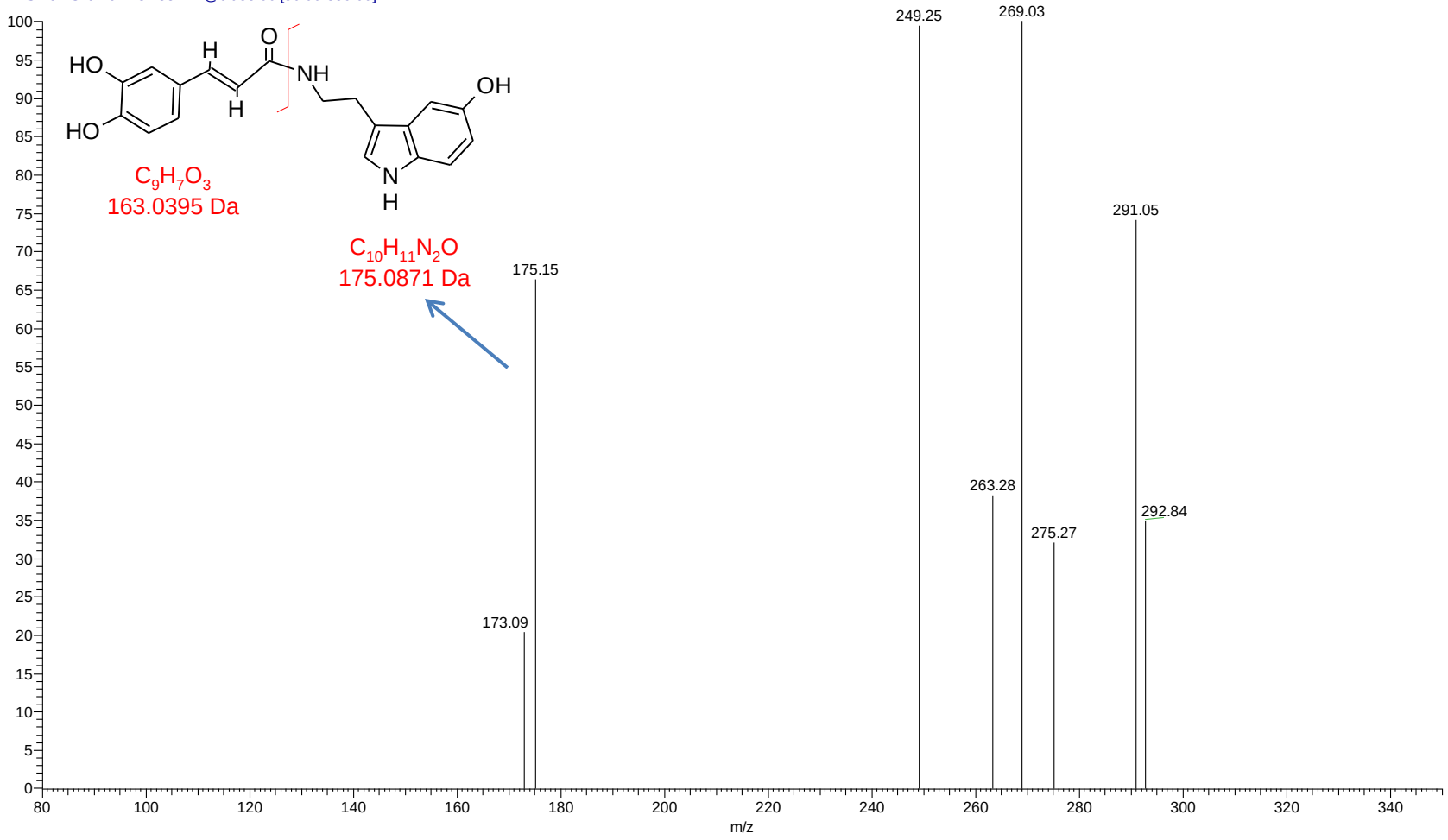

(h) Feruloylserotonin

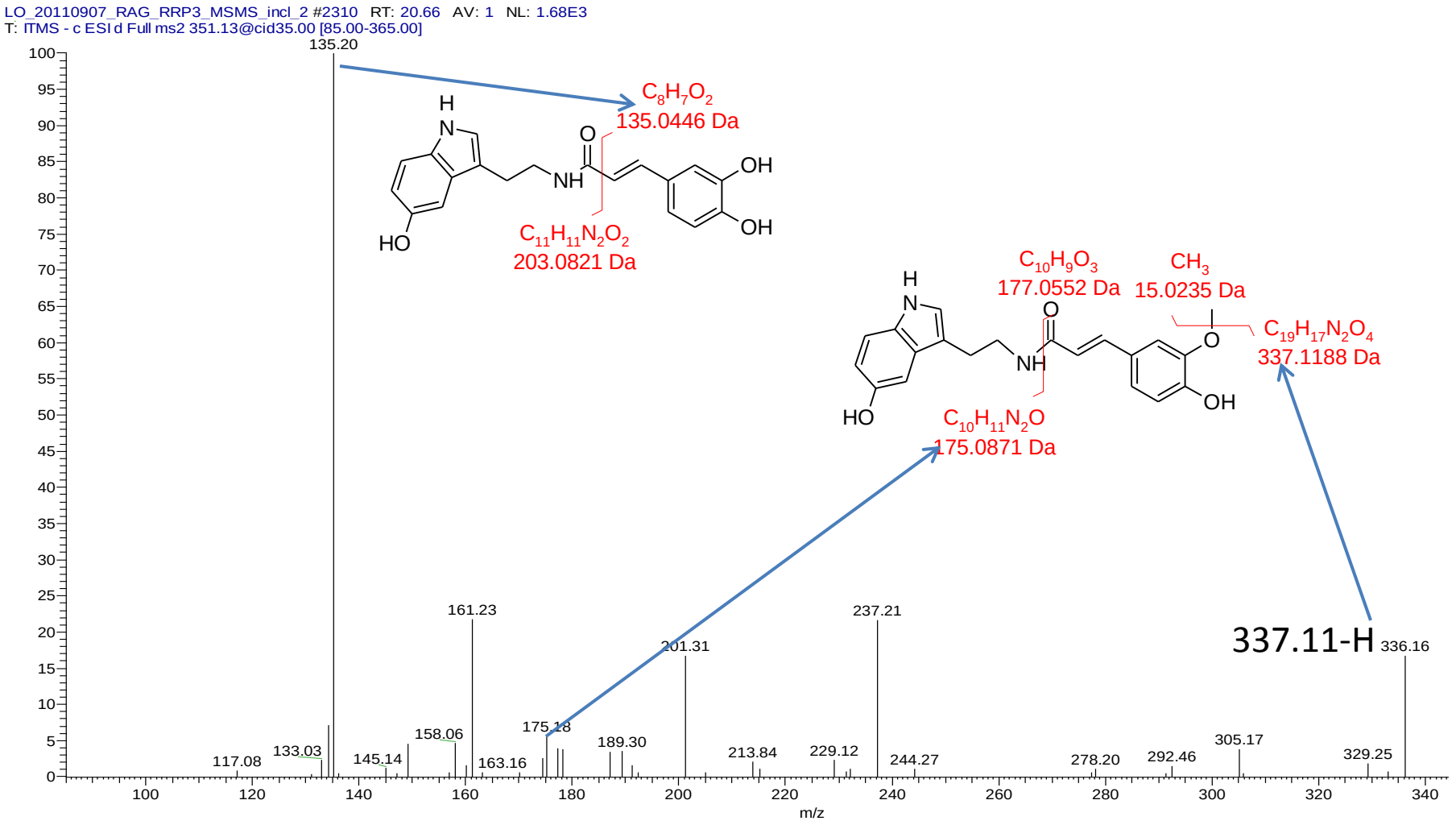

(i) Caffeyl alcohol

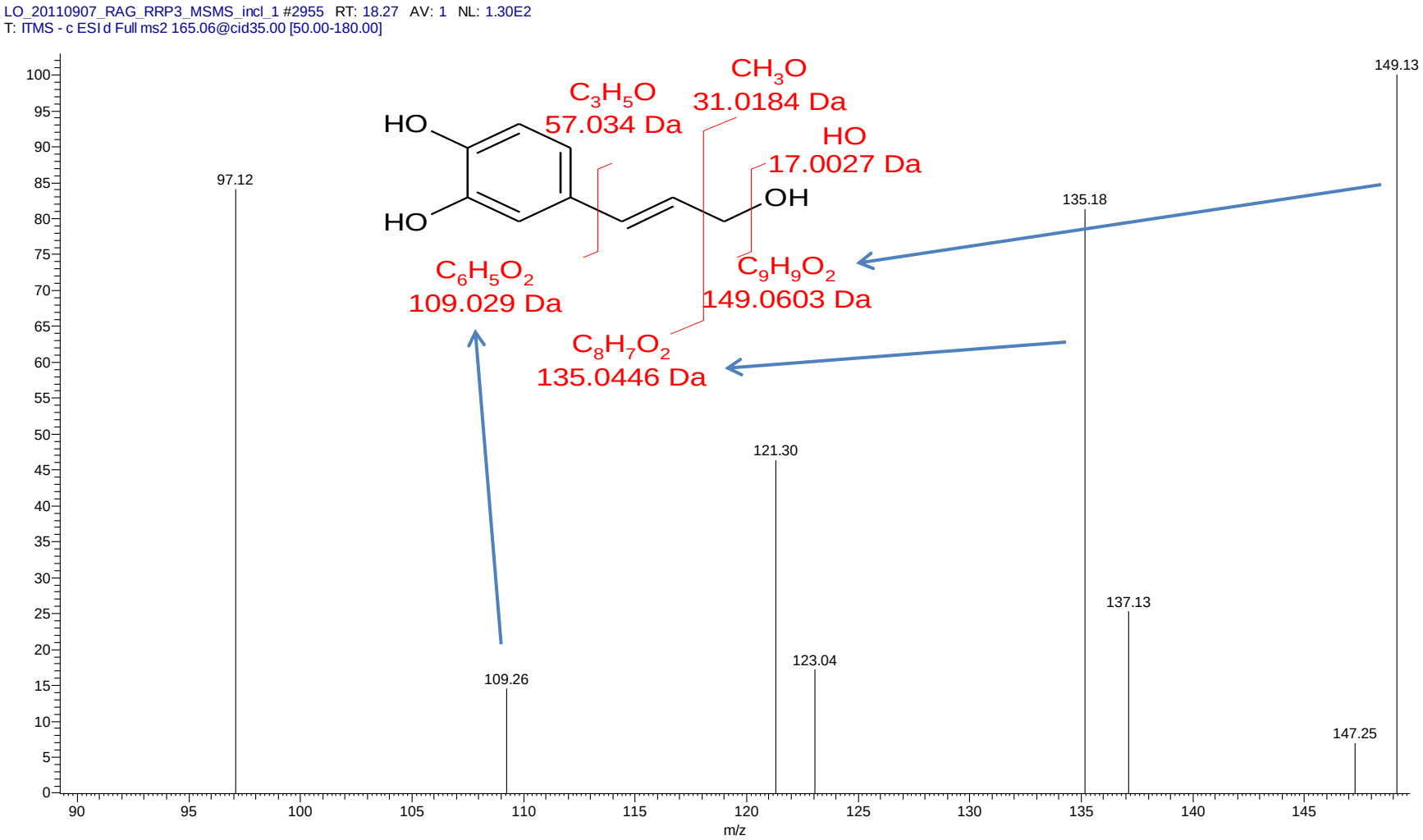

(j) 4-coumaroylshikimate

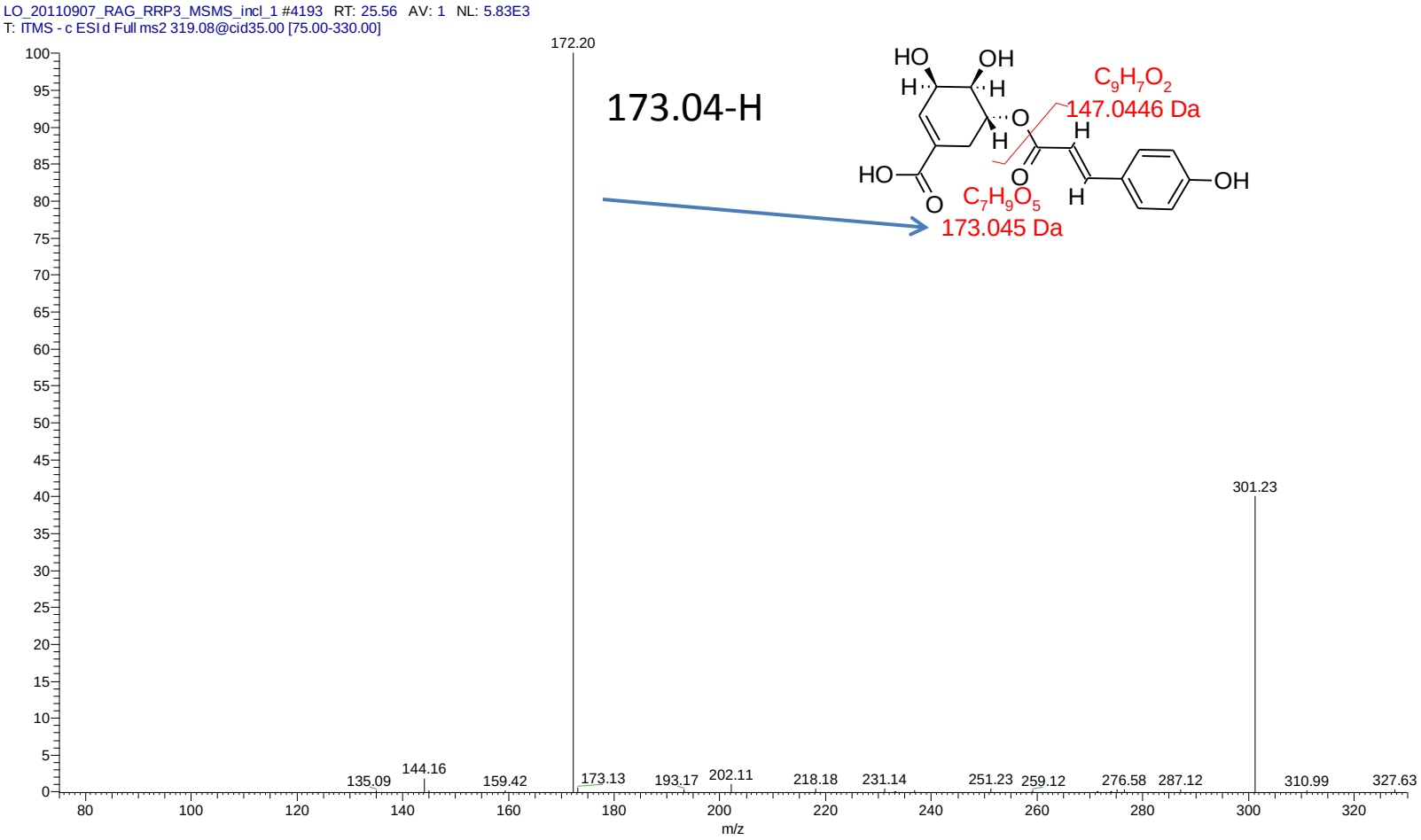

(k) 4-coumaroylquinate

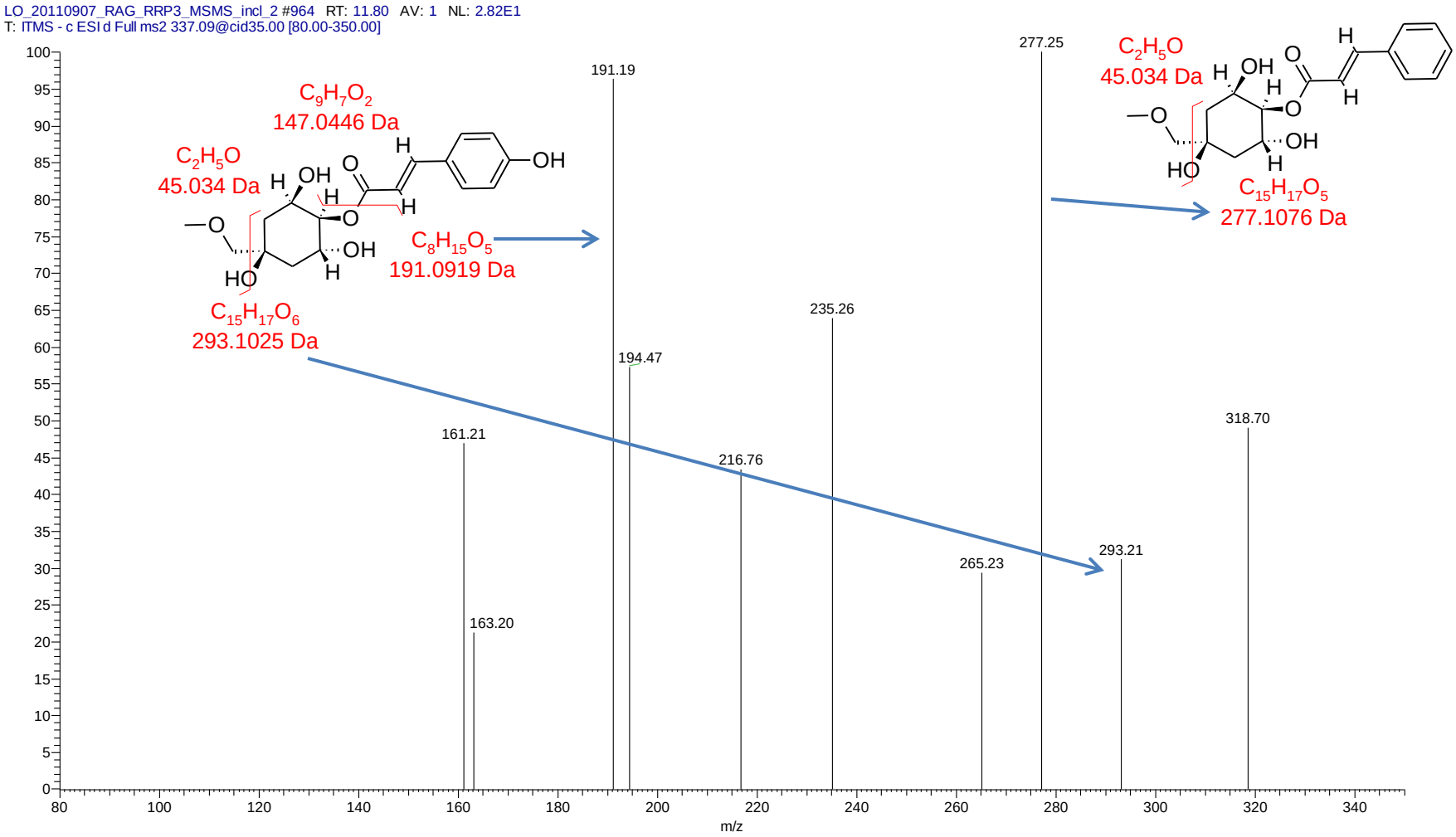

(I) β-D-glucopyranosyl-caffeic acid

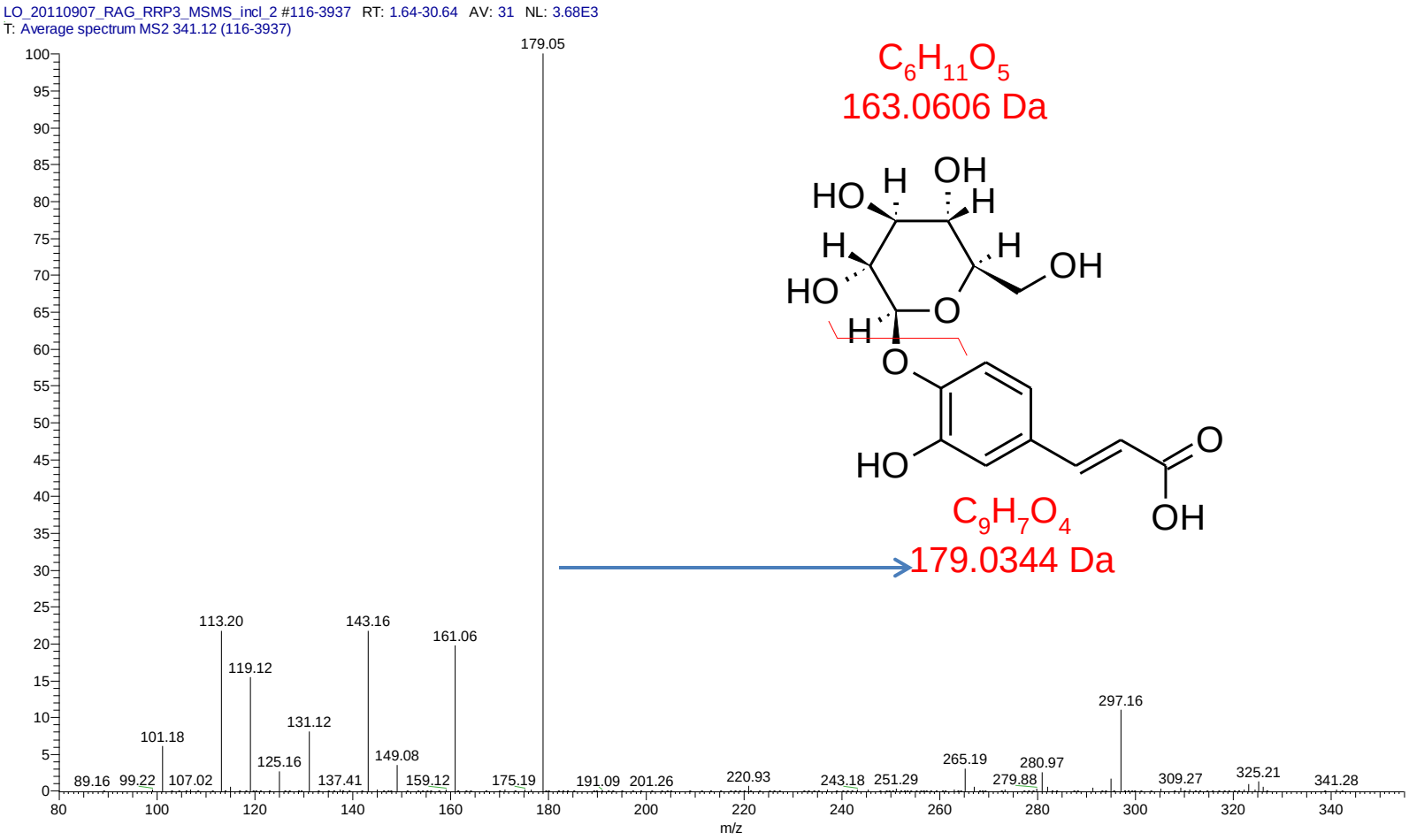

(m)Ferulic acid 7-O-glucoside

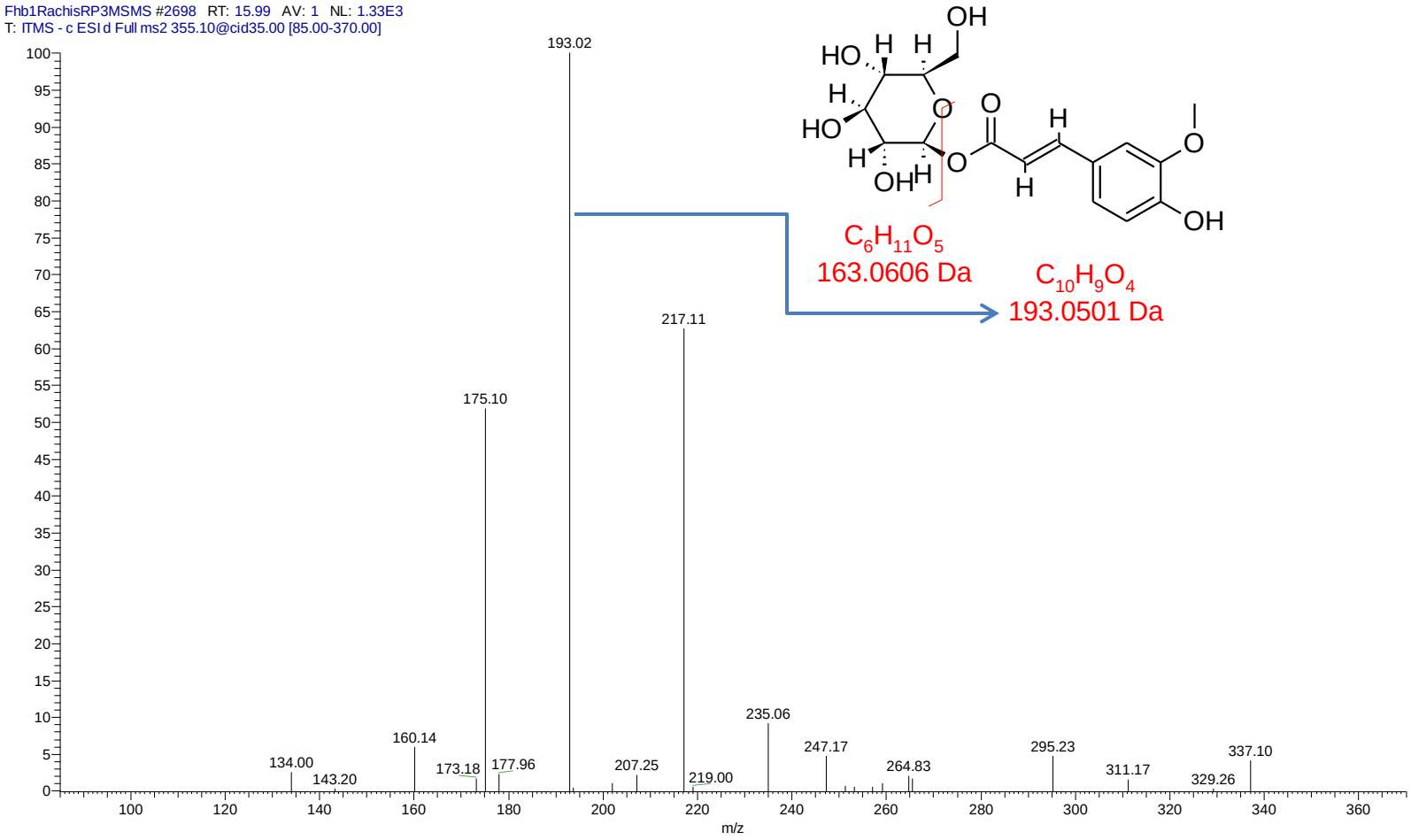

(n) β -D-glucopyranosyl-sinapic acid

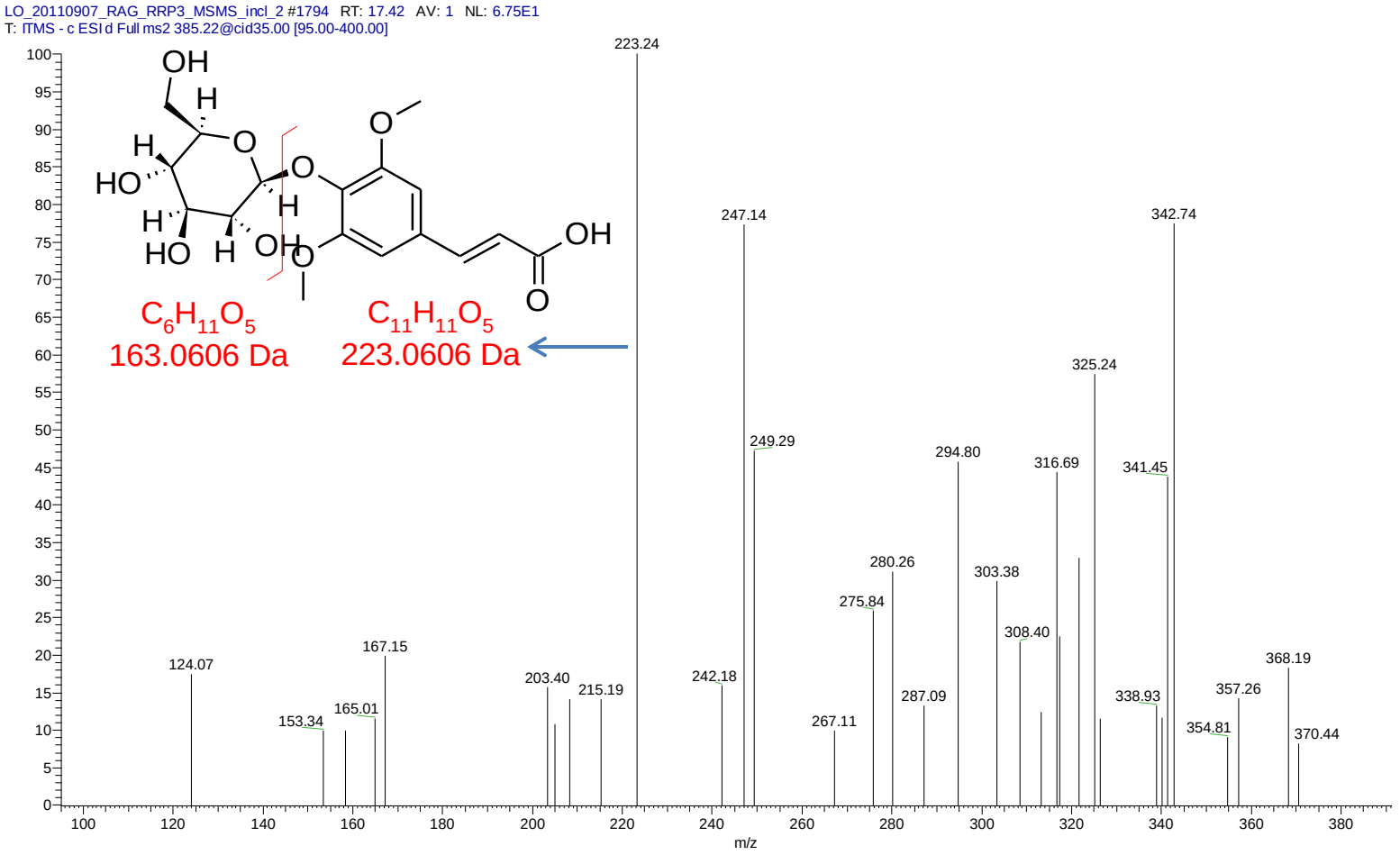

(o) Salicylic acid 2-O-β-D-glucoside

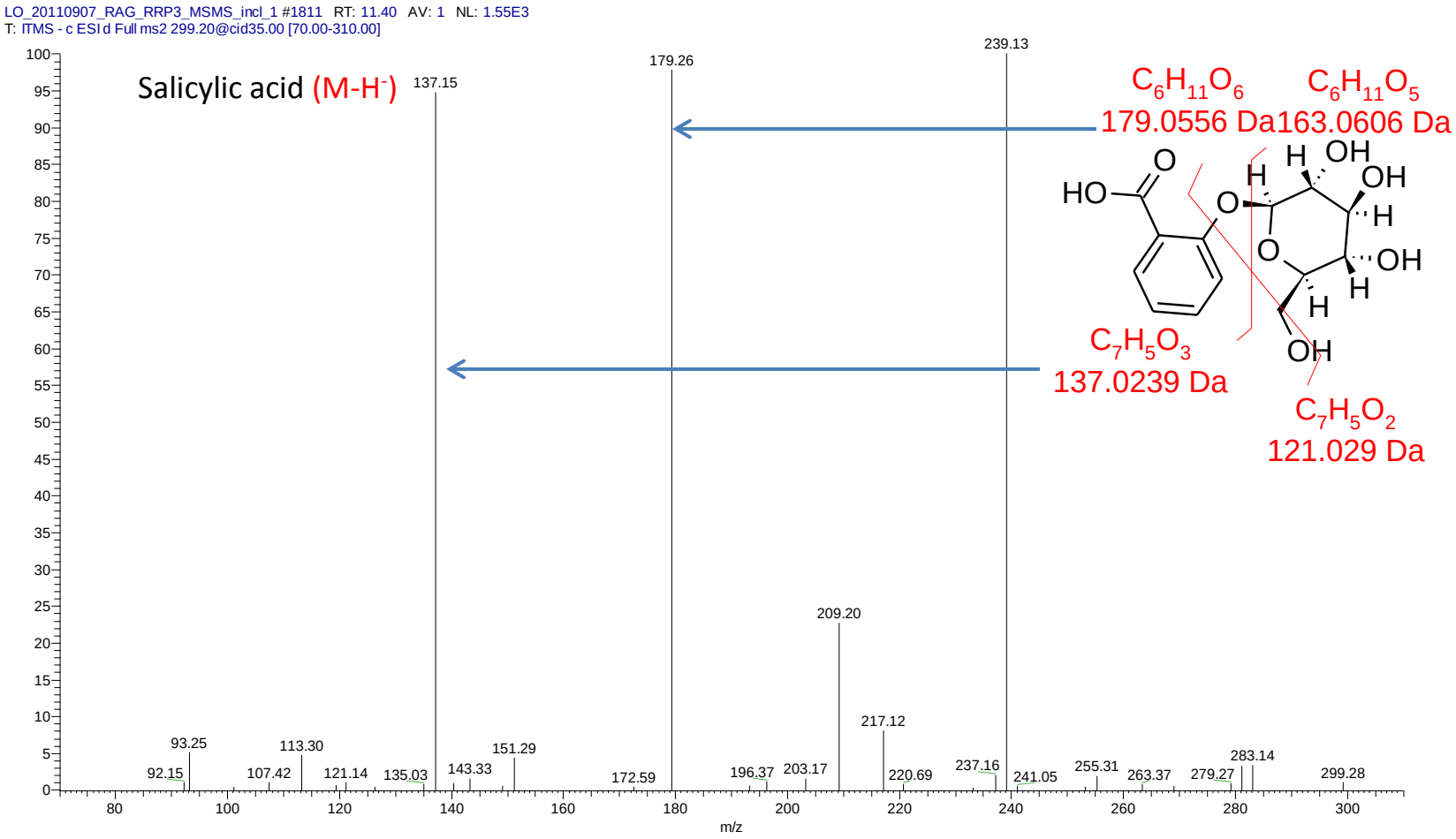

(p) Jasmonoyl-L-isoleucine

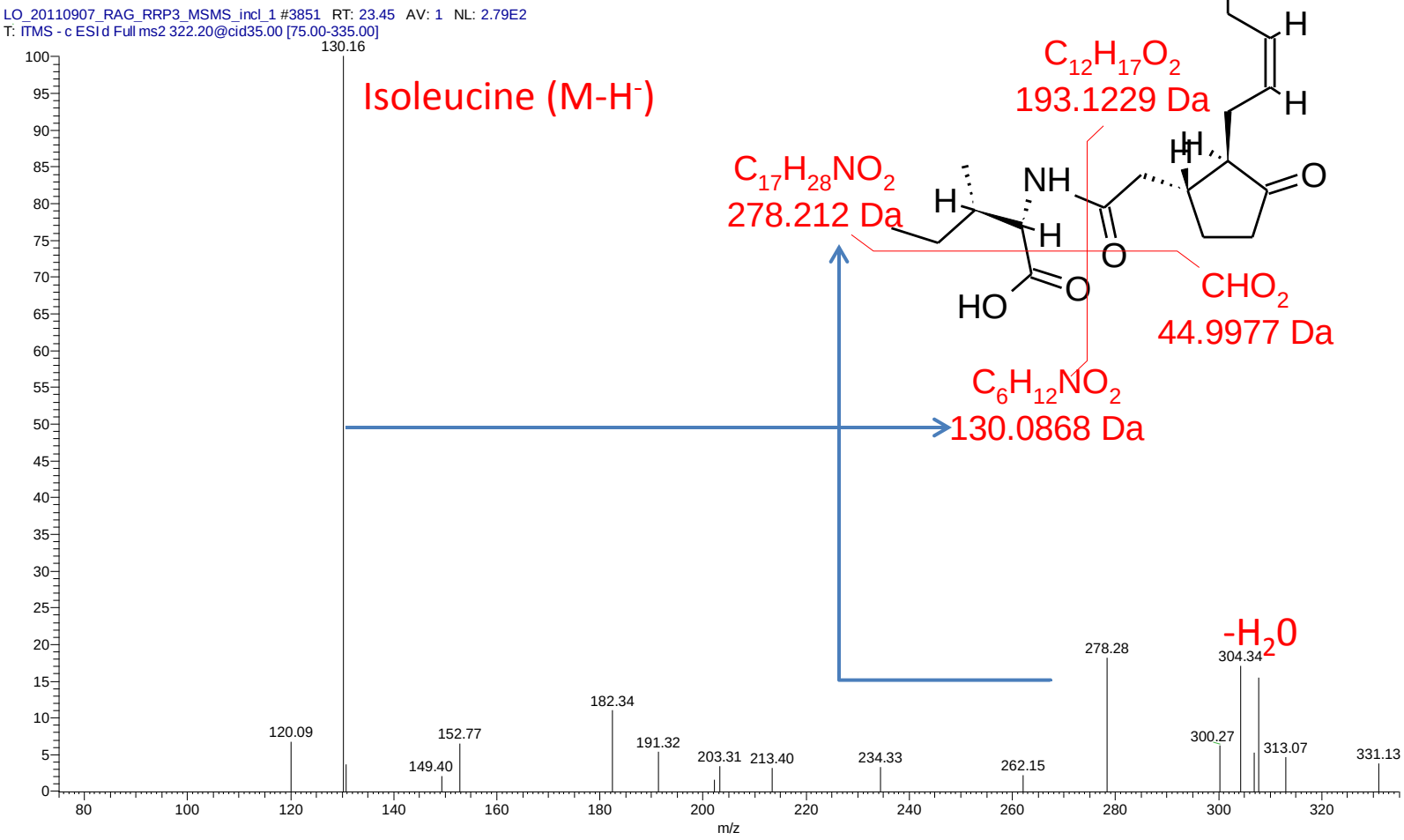

(q) Abscisic aldehyde

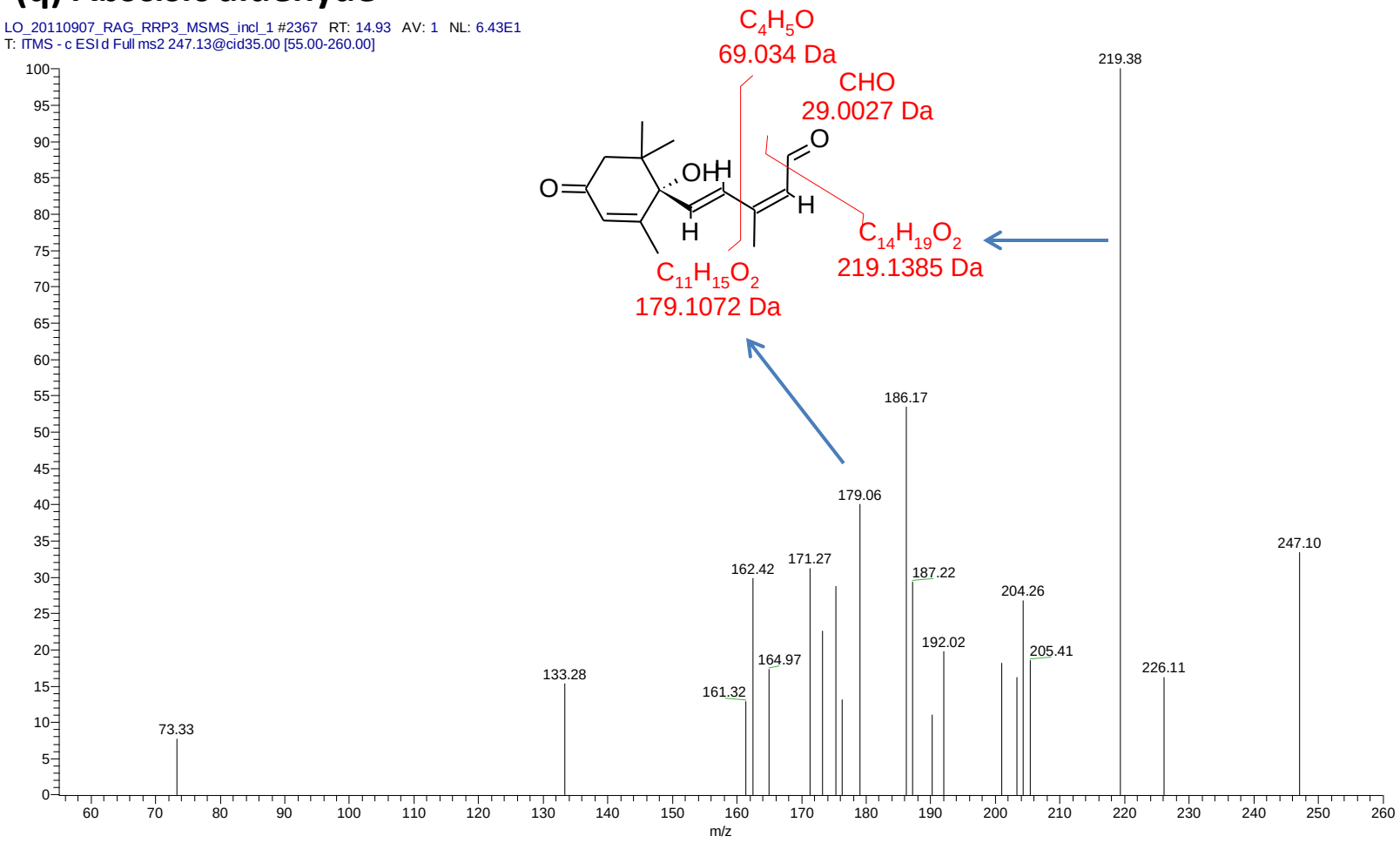

Supplement: Figure S2 — MS/MS spectra of metabolites detected in wheat NILs and in silico fragments verification of metabolites. (PDF) [file pone.0040695.s002.pdf]
