# Supplementary material for: Integrated Metabolo-Proteomic Approach to Decipher the Mechanisms by Which Wheat QTL (Fhb1) Contributes to Resistance against Fusarium graminearum
Source: PLoS One. 2012 Jul 12;7(7):e40695. doi: 10.1371/journal.pone.0040695 (PMC3398977; doi:10.1371/journal.pone.0040695)
Supplement: Figure S3 — Resistant related induced (RRI) proteins in wheat NIL with resistant Fhb1allele following F. graminearum inoculation. (PDF) [file pone.0040695.s003.pdf]

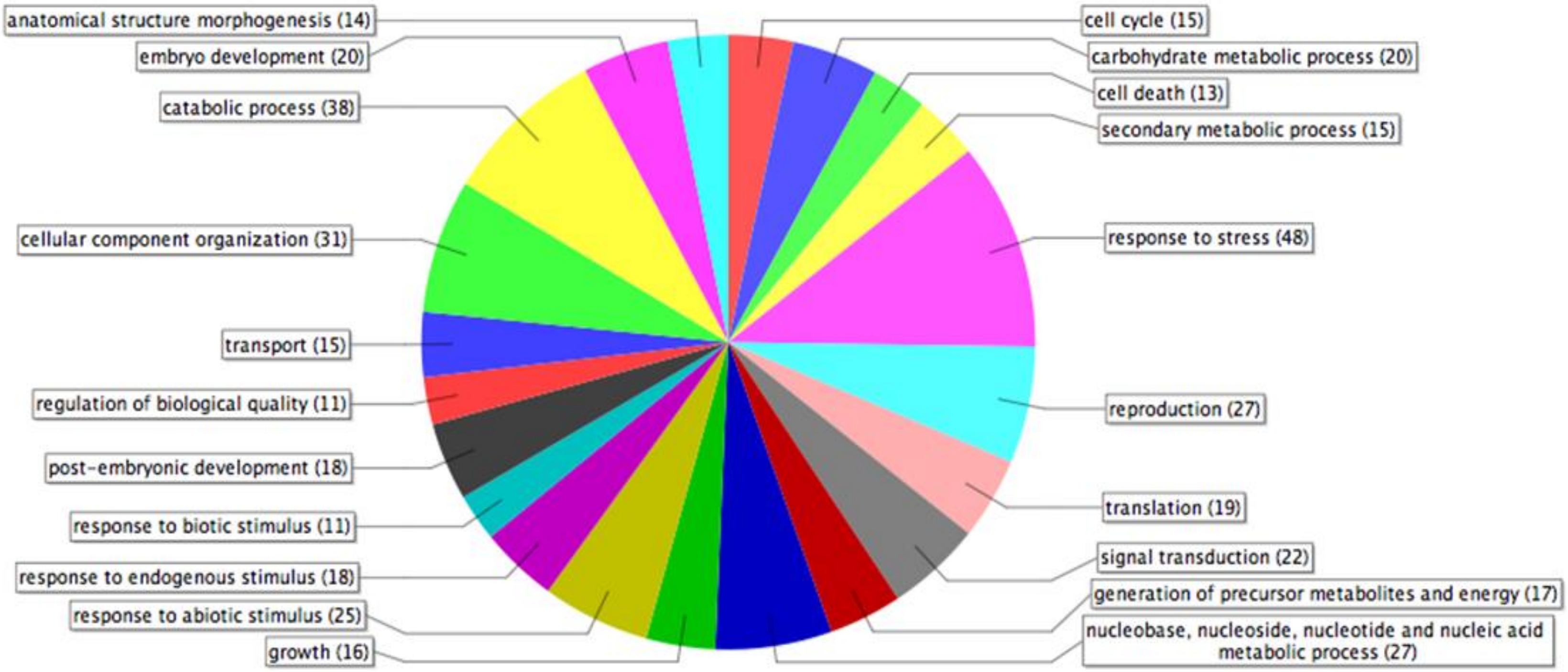

Fig. S3 Resistant related induced (RRI) proteins in wheat NIL with resistant *Fhb1*allele following *F. graminearum* inoculation.
