# Supplementary figures and images for: Integrated Metabolo-Proteomic Approach to Decipher the Mechanisms by Which Wheat QTL (Fhb1) Contributes to Resistance against Fusarium graminearum
Source: PLoS One. 2012 Jul 12;7(7):e40695. doi: 10.1371/journal.pone.0040695 (PMC3398977; doi:10.1371/journal.pone.0040695)

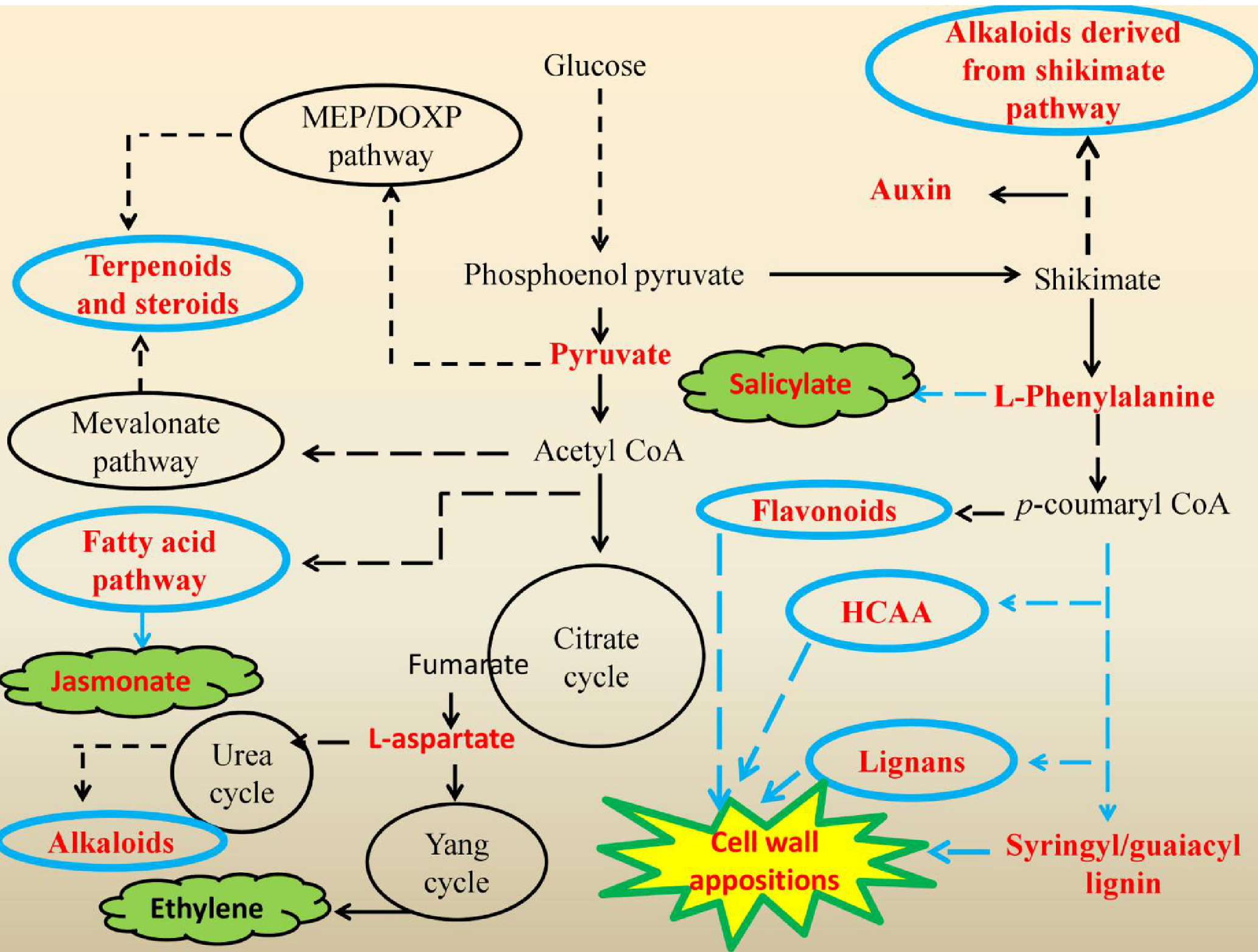

Supplement: Figure S4 — Satellite metabolic pathways of wheat-Fusarium interaction. (TIF) [file pone.0040695.s004.tif]
