# Supplementary material for: Integrated Metabolo-Proteomic Approach to Decipher the Mechanisms by Which Wheat QTL (Fhb1) Contributes to Resistance against Fusarium graminearum
Source: PLoS One. 2012 Jul 12;7(7):e40695. doi: 10.1371/journal.pone.0040695 (PMC3398977; doi:10.1371/journal.pone.0040695)
Supplement: Table S1 — Fusarium head blight resistance related metabolites identified in rachises of wheat NIL with resistant Fhb1 allele following F. graminearum or mock inoculation. (DOC) [file pone.0040695.s005.doc]

**Table S1:** Fusarium head blight resistance related metabolites in wheat QTL *Fhb1* identified in **rachis**, following *F. graminearum* or mock inoculation

| **Observed mass (Da)** | **Exact mass (Da)** | **AME** | **RT (min)** | **Putative** inoculation **name** | **Observed fragmentation** | **Database fragmentation** | **Fold change@** | **Chemical group** | **Database ID^$^** |
| --- | --- | --- | --- | --- | --- | --- | --- | --- | --- |
| 130.0270 | 130.0266 | 2.92 | 1.61 | Itaconic acid | **129.10**, **111.07**, 101.25, 99.11, **85.20** | 129.01, 111.04, 85.02, 41.04 | 1.30 (RRI), 1.7 (PRr), | Fatty acids and Conjugates | 44764, C00490, **KOX00366**, LMFA01170063 |
| 132.0426 | 132.0423 | 2.00 | 1.60 | 2-methylenesuccinic acid | **113.18, 87.30** | 131.03, 113.02, 87.04, 69.03 | 1.69*(RRI), 2.2*(PRr) | Fatty acids and Conjugates | 3254, C00489, **KOX00277**, LMFA01170046 |
| 134.0370 | 134.0368 | 1.00 | 13.58 | 6Z-Octene-2,4-diynoic acid | 115.28, 75.18, 59.10 |  | 4.9*(RRI), 5.8*(PRr) | Fatty Acids and Conjugates | 35332 |
| 136.0527 | 136.0524 | 1.00 | 14.70 | Hydroxyacetophenone | 135.18, 107.23, 91.31, 75.25 |  | 3.8*(***RRI***) | Phenylpropanoid | 64173, C06224 |
| 136.0527 | 136.0524 | 1.00 | 13.54 | Methylbenzoate | 135.27, 117.54, 107.25, **91.24, 75.30** | 135.04, 91.05, 89.01, 75.00, 59.01, | 3.9*(***RRI***) | Aldehydes | 63506, C03765, **KOX00415** |
| 138.0320 | 138.0317 | 2.52 | 14.16 | Salicylic acid | **136.84,** 118.99, 108.93, **92.84** | 137.02, 93.03, 65.03 | 3.2*(***RRI***) | Phenol | 3263, C00156, **KOX00321** |
| 148.0527 | 148.0524 | 2.09 | 1.66 | *trans*-Cinnamic acid | 129.13, 103.11, 87.17, 85.18 | 147.0, 103.05, 101.04, 77.04 | 1.27*(RRI), 1.9*(PRr) | Phenylpropanoid | 63104, C00423, **PR050604** |
| 162.0681 | 162.0680 | 1.00 | 1.60 | Methylcinnamate | 161.03, 143.14, 131.05, 117.24, |  | 1.13*(RRI), 1.7*(PRr) | Phenylpropanoid | 3793 |
| 166.0631 | 166.0630 | 0.70 | 18.01 | Caffeyl alcohol | **149.13, 135.18,** 121.30, **109.26,** 97.12 | 149.13, 135.18, 109.26 | 1.35*(RRI), 10*(PRr) | Phenylpropanoid | 64162, C12206, ***In silico*** |
| 175.0635 | 175.0633 | 1.00 | 9.75 | 3-Indoleacetic Acid | **174.01,** 146.04, **130.15,** 129.09, 115.09 | 174.05, 130.06, 128.04 | 123.9*(***RRI***) | Auxin | 70, **KOX00380** |
| 176.0941 | 176.0940 | 0.80 | 35.14 | Serotonin | 175.23, 147.13, 131.08 |  | 1.33*(RRI), 1.6*(PRr) | Amines | C00780, PMN |
| 180.0636 | 180.0634 | 1.21 | 1.59 | Myoinositol | 179.12, **161.06, 143.11**, **125.08,** 99.12, 97.06 | 180.03, 161.0, 149.0, 143.1, 131.0, 125.1, 119.1, 113.1, 89.1 | 1.8*(***RRI***) | Carbohydrates and Carbohydrate conjugates | C00137**, MT000112** |
| 192.0634 | 192.0634 | 0.19 | 1.51 | Quinic acid | **191.26, 173.07, 171.0, 110.96** | 191.3, 173.4, 170.8, 127.0, 111.4, 93.0, 96.1 | 1.13*(RRI), 2.5*(PRr) | Carboxylic acid | 3329, C00296, **KO001747** |
| 202.1206 | 202.1205 | 0.26 | 18.31 | Sebacic acid | **201.09,** 157.13, 118.97, 58.89 | 201.0, 183.0, 139.0 | 1.25*(RRI), 1.5*(PRr) | Fatty Acids and Conjugates | 4240, C08277, **PR051363,** LMFA01170006, C00001202 |
| 208.0722 | 208.0736 | 6.45 | 22.44 | Sinapaldehyde | **206.90**, **192.06, 177.33,** 163.20, **146.8,** 133.18, 120.09, 103.05 | 207.0, 192.0, 177.0,149.0, 93.0 | 2.22*(RRI), 14.2*(PRr) | Phenylpropanoid | 44806, C05610, **PR051195**, C00002775, **McGill MD** |
| 210.1255 | 210.1256 | 0.35 | 17.07 | Jasmonic acid | 194.12, **191.01, 165.03,** 149.94, **143.01,** 127.18, 111.04 | 209.29, 191.00, 165.40, 143.05, 133.67 | 1.14**(RRI), 4**(PRr) | Jasmonate | **62988**, C08491, C00001314, **McGill MD** |
| 224.0700 | 224.0685 | 7.00 | 19.94 | Sinapic acid | **223.18, 208.0,** 179.02, 145.04, **141.04** | 223.08, 208.03, 193.01, 164.04, 149.02, 141.01 | 2.6*(RRC) | Phenylpropanoid | 45738, C00482, **KOX00565, McGill MD** |
| 234.1367 | 234.1368 | -0.34 | 14.23 | *p*-Coumaroylputrescine | **233.11, 218.25,** 191.13,**119.01** | 119.04, 190.08, 218.11, 233.12 | 24.6** (***RRI***) | Hydroxycinnamic acid amide | C18326, PMN, J817.813E, ***In silico,*** |
| 248.1423 | 248.1412 | 4.34 | 15.36 | Abscisic aldehyde | 247.10, **219.38,** 186.17, **179.06** | 219.38, 179.06 | 163.1* (***RRI***) | Terpenoid | C13455, J489.427H, PMN, ***In silico*** |
| 250.1568 | 250.1569 | 0.54 | 24.50 | Xanthoxin | 249.53, 205.23, 181.00, 125.23, 119.13 |  | 2.68*(RRI), 13.9*(PRr) | Terpenoid | 64102, C13453 |
| 250.1568 | 250.1569 | 0.44 | 20.71 | Abscisic alcohol | 119.14 |  | 2.67*(RRI), 15.8*(PRr) | Terpenoid | 64130, C13456, |
| 264.1036 | 264.1030 | 2.42 | 13.58 | 2-(6'-methylthio)hexylmalate | 248.01, 245.07, 235.23, 219.22, 176.22 |  | 1.01*(RRI), 20.6*(PRr) | Glucosinolate | PMN |
| 264.1473 | 264.1474 | 0.32 | 15.11 | Feruloylputrescine | **248.04,** 235.32, 219.33, **176.19,** 160.25 | 176.04, 248.12 | 407.8* (***RRI***) | Hydroxycinnamic acid amide | C10497, J11.793E , ***In silico,*** |
| 267.1268 | 267.1259 | 3.21 | 13.55 | Cinnamoyltyramine | 135.32, **136.13,** 182.35 | 131.04, 136.07 | 262.8* (***RRI***) | Hydroxycinnamic acid amide | PMN, J466.382I, ***In silico*** |
| 272.2352 | 272.2351 | 0.00 | 31.64 | 4-Hydroxy palmitic acid | 271.38, 253.43, 243.23, 227.27 |  | 1.6*(RRI), 1.6*(PRr) | Fatty Acids and Conjugates | 35428, LMFA01050050 |
| 276.0244 | 276.0246 | 0.84 | 1.40 | 6-Phospho-D-gluconate | **257.16, 177.07, 159.16, 97.10** |  | 1.18*(RRI), 1.3*(PRr) | Carbohydrates and Carbohydrate conjugates | 367, C00345 |
| 276.1584 | 276.1586 | 0.96 | 16.16 | *cis*-*p*-Coumaroylagmatine | **258.29, 233.30, 119.18** | 119.04, 233.129, 258.14 | 44.3* (***RRI***) | Hydroxycinnamic acid amide | C00028060, ***In silico* ,** |
| 280.1085 | 280.1099 | 5.10 | 13.56 | Magnaldehyde B | 264.28, 239.27, 149.16, 134.12 |  | 1.33*(RRI), 189.8*(PRr) | Lignan | C00030707 |
| 280.1309 | 280.1311 | 0.79 | 22.44 | 8'-hydroxyabscisate | 279.21, 261.29, 195.07 |  | 529.4* (***RRI***) | Terpenoid | PMN |
| 280.2403 | 280.2402 | 0.08 | 31.90 | Linoleic acid | **279.26, 261.24, 235.38, 191.09,** | 279.23, 261.22, 235.16, 219.17, 191.12 | 1.5*(RRC) | Fatty Acids and Conjugates | 191, C01595, **KOX00402,** LMFA01030120, **McGill MD** |
| 282.0887 | 282.0892 | 1.00 | 12.69 | 5,6-Dimethoxyflavone | 281.10, 263.14, 237.11, 219.28, 179.21 |  | 126.6* (***RRI***) | Flavonoids | 48536, , , LMPK12110102 |
| 288.0622 | 288.0633 | 3.78 | 13.57 | 2-Hydroxyisoflavanone naringenin | 277.24, 257.10, 249.31, |  | 16.3* (***RRI***) | Flavonoids | PMN |
| 292.1560 | 292.1575 | 5.07 | 16.05 | 16-epivellosimine | 274.14, 249.26, 119.14 |  | 35.3* (***RRI***) | Alkaloid | PMN |
| 296.2351 | 296.2351 | 0.18 | 29.98 | 9S-hydroxy-10E,12Z-octadecadienoic acid (9(S)-HODE) | **295.37, 277.36, 171.27** | 295.22, 277.21, 171.10 | 1.6*(RRC) | Fatty Acids and Conjugates | **45662,** LMFA02000057 |
| 300.0841 | 300.0845 | 1.33 | 11.38 | Salicylic acid O-β-D-glucoside | 239.13, 209.20, **179.26, 137.15** | 179.26, 137.15 | 1.03*(RRI), 3.1*(PRr) | Aryl glucoside | PMN, J355.695F, ***In silico*,** |
| 306.1362 | 306.1368 | 1.96 | 14.71 | Cinnamoylserotonin | **289.24, 219.23,** 175.16, 97.13 | 175.08, 289.13 | 1.06*(RRI), 33.7*(PRr) | Hydroxycinnamic acid amide | PMN, J958.892B, ***In silico*** |
| 306.1688 | 306.1692 | 1.25 | 16.85 | Feruloylagmatine | **289.25, 263.15, 177.41, 149.12,** 134.18 | 289.14, 263.13, 177.05, 149.06 | 104.2 * (***RRI***) | Hydroxycinnamic acid amide | PMN, J817.816J, ***In silico* ,** |
| 309.1935 | 309.1940 | 1.52 | 14.71 | Jasmonoyl valine |  |  | 79.1* (***RRI***) | Jasmonate | PMN, J2.953.029G |
| 310.2138 | 310.2144 | 1.95 | 26.99 | 13(S)-Hydroperoxylinolenic acid | **291.23, 209.12,** 171.13, **165.22,** 155.07 | 309.20, 291.19, 247.19, 209.11, 165.22 | 1.5*(RRC) | Fatty Acids and Conjugates | 36025, C16321, **UT000087** |
| 320.0888 | 320.0896 | 2.46 | 25.45 | 4-coumaroylshikimate | 301.23, **172.20** | 172.03 | 85.1* (***RRI***) | Phenylpropanoid | PMN, J2.380.119A, ***In silico*** |
| 322.1321 | 322.1317 | 1.03 | 15.94 | *p*-coumaroylserotonin | 97.19, 149.08, **175.16, 219.17, 289.17** | 289.13, 175.08 | 99.1* (***RRI***) | Hydroxycinnamic acid amide | PMN, J2.159.664G, ***In silico*** |
| 323.2093 | 323.2096 | 0.79 | 23.57 | (+)-7-iso-jasmonoyl-L-isoleucine | **304.34, 278.28,** 182.34, **130.16** | 304.34, 278.28, 130.16 | 1.76*(RRI), 4.6*(PRr) | Jasmonate | PMN, J272.686F, ***In silico*** |
| 326.1523 | 326.1518 | 1.59 | 11.45 | Dehydrodiisoeugenol | 265.19, 324.19, 281.05, 227.02, 163.09, 296.85 |  | 1.7*(RRC) | Lignan | C10650, C00002611 |
| 328.1880 | 328.1889 | 2.72 | 17.80 | 1-pentyl-sn-glycero-3-phosphocholine | 312.23, 309.25, 291.14, 267.21, 175.07 |  | 97* (***RRI***) | Glycerophospholipid | 40394, LMGP01060020 |
| 338.0993 | 338.1001 | 2.35 | 13.52 | 4-coumaroylquinate | 161.21, **191.19,** 235.26, **277.25, 293.21,** 318.70 | 293.10, 191.09, 277.01 | 43.1* (***RRI***) | Phenylpropanoid | PMN, , ***In silico*, Torras-Claveria et al. (2011)** |
| 338**.**1258 | 338.1266 | 2.30 | 17.86 | Caffeoylserotonin | **175.15,** 249.25, 269.03, 291.05 | 175.08 | 2.45*(RRI), 167.2*(PRr) | Hydroxycinnamic acid amide | PMN, ***In silico*** |
| 340.0592 | 340.0583 | 2.77 | 1.57 | 7-Methoxy-5,6:3\',4\'-bis(methylenedioxy)flavone | 320.93, 278.13, 241.22, 159.17, 154.19 |  | 1.6*(RRC) | Flavonoids | 49668, LMPK12111244 |
| 342.1002 | 342.0950 | 5.20 | 20.95 | β-D-glucopyranosyl-caffeic acid | 113.20, 143.16, 161.06, **179.05,** 297.16 | 163.06, 179.03 | 2.6 (RRI), 4.0 *(PRr) | Phenylpropanoid | J460.644B, *In silico*, **Torras-Claveria et al. (2011)** |
| 342.1308 | 342.1315 | 2.00 | 12.73 | Coniferin | 294.96, 281.31, 265.25, **179.11, 163.24** | 179.11, 163.24 | 4.0* (PRr) | Phenylpropanoid | 64182, C00761 |
| 344.0891 | 344.0896 | 1.39 | 22.83 | 3',5-Dihydroxy-4',6,7-trimethoxyflavone | 328.15, 275.27 |  | 1.4**(RRC) | Flavonoids | 43851, LMPK12111239 |
| 344.0894 | 344.0896 | 0.47 | 21.65 | 5,6-Dihydroxy-7,8,4'-trimethoxyflavone | 328.16, 275.20 |  | 1.7*(RRC) | Flavonoids | 49872, LMPK12111449 |
| 344.1458 | 344.1471 | 4.00 | 17.06 | Dihydroconiferyl alcohol glucoside | 275.21 |  | 10** (***RRI***) | Terpenoid | 41169,  C11653, LMPR0102070024, |
| 346.1260 | 346.1263 | 1.99 | 11.87 | Aucubin | 330.84, 299.22, 281.16 |  | 2.6* (***RRI***) | Terpenoid | 41151,  C09771, LMPR0102070006 |
| 346.1261 | 346.1263 | 0.00 | 13.98 | Deutzioside | 331.00, 299.11, 281.09, 227.22 |  | 1.56*(RRI), 6.4*(PRr) | Terpenoid | 41181,  C11671, LMPR0102070036 |
| 350.1636 | 350.1630 | 1.52 | 14.60 | Vomilenine | 333.26, 319.28, 305.41, 291.39, 255.77 |  | 1054.4** (***RRI***) | Alkaloid | PMN |
| 352.1421 | 352.1423 | 0.65 | 20.67 | Feruloylserotonin | **336.16,** 237.21, 201.31, **175.18, 177.01, 161.23, 135.20** | 135.04, 175.08, 177.05, 336.11 | 1194.8* (***RRI***) | Hydroxycinnamic acid amide | PMN, J1.973.598B, ***In silico*** |
| 356.1101 | 356.1107 | 1.00 | 16.63 | Ferulic acid 7-O-glucoside | 217.06, 193.01, 175.01 | 163.06, 193.02 | 28.7 * (***RRI***) | Phenylpropanoid | J322.813D, ***In silico*,** |
| 360.1416 | 360.1420 | 1.06 | 13.05 | 7-Deoxyloganate | 340.17, 299.21, 239.18, 197.23 |  | 1.4* (RRC) | Terpenoid | 64040, C11636 |
| 360.1566 | 360.1573 | 1.95 | 20.26 | Lariciresinol | **344.15,** 327.12, 299.15, 239.00, 197.08 | 344, 313 | 1.95*(RRI), 13.5*(PRr) | Lignan | C10646,   C00000602, |
| 370.1265 | 370.1264 | 0.32 | 18.48 | Sinapaldehyde glucoside |  |  | 93.1* (***RRI***) | Phenylpropanoid | PMN, |
| 370.1790 | 370.1780 | 2.59 | 22.29 | Unanisoflavan | 354.07, 323.27, 201.12, 185.02, 166.93 |  | 2.5*(RRC) | Flavonoids | 48254, LMPK12080009, C00009734 |
| 372.1425 | 372.1420 | 1.16 | 13.03 | Syringin | **353.27,** 310.6, **249.26, 209.23,** 149.13 | 353, 311, 209 | 1.92 (RRI), 60* (PRr) | Phenylpropanoid | 64181, C01533, |
| 373.1294 | 373.1287 | 1.96 | 1.49 | 7-Oxomatairesinol | 356.10, 288.69, 249.04, 231.20, 175.24, 120.94 |  | 1.04*(RRI), 4.5*(PRr) | Lignan |  |
| 384.1209 | 384.1215 | 1.58 | 17.87 | 2-S-adenosyl-L-homocysteine | 365.24, 355.13, 337.06, 307.11, 296.02, 247.28 |  | 132.6* (***RRI***) | non-standard alpha amino acid | PMN |
| 386.1210 | 386.1212 | 0.8 | 16.98 | β -D-glucopyranosyl-sinapic acid | **223.24,** 247.14, 342.74 | 223.06, 163.06 | 2.8 (RRI), 4.8*(PRr) | Phenylpropanoid | J1.426.998C, ***In silico*** |
| 390.1508 | 390.1526 | 4.00 | 16.02 | Loganin | 371.42, 341.22, 291.16 |  | 1.70*(RRI), 18.8*(PRr), | Terpenoid | 41146, C01433, LMPR0102070001 |
| 398.1356 | 398.1366 | 2.41 | 20.79 | Deoxypodophyllotoxin | 379.22, 336.86, 329.26, 311.40 |  | 1.4*(RRC) | Lignan | 2040, C10556, |
| 399.1437 | 399.1450 | 3.18 | 24.29 | S-adenosyl-L-methionine | 329.38 |  | Inf* (***RRI***) | non-standard alpha amino acid | PMN |
| 402.1519 | 402.1526 | 1.62 | 13.79 | Benzyl alcohol beta-D-xylopyranosyl (1->6)-beta-D-glucopyranoside | 383.23, 357.37, 179.25 |  | 23.3* (***RRI***) | Carbohydrates and Carbohydrate conjugates | PMN |
| 406.1467 | 406.1475 | 1.00 | 13.99 | 10-Hydroxyloganin | 387.34, 344.93, 183.28, 161.02 |  | 1.01*(RRI), 7.3*(PRr) | Terpenoid | 41173, C11659, LMPR0102070028 |
| 426.1881 | 426.1890 | 2.14 | 22.36 | Abscisic acid glucose ester | 305.18, 275.25, 261.24 |  | 189.1* (***RRI***) | Terpenoid | PMN |
| 428.1462 | 428.1471 | 0.00 | 20.88 | 5,4\'-Dihydroxy-3,6,3\'-trimethoxy-7-prenyloxyflavone | 409.19, 395.10, 351.17, 281.16, 263.13, 249.06 |  | 1.20*(RRI), 7*(PRr) | Flavonoids | 51635, LMPK12113223 |
| 434.1219 | 434.1213 | 1.32 | 20.69 | Naringenin 7-O-β-D-glucoside | 403.38, 373.29, 364.86, 351.25, 312.14, 206.86 |  | 23.7* (***RRI***) | Flavonoids | 52730, PMN |
| 446.1567 | 446.1577 | 2.00 | 20.90 | 5-Hydroxy-7,8-dimethoxyflavanone 5-rhamnoside | 429.60, 417.11, 353.29, 327.23 |  | 74.4* (***RRI***) | Flavonoids | 53130, PMN |
| 550.1678 | 550.1686 | 1.00 | 16.32 | Chalconaringenin 2\'-rhamnosyl-(1->4)-xyloside | 530.64, 387.22, 369.17, 265.11 |  | 6.7***(RRC) | Flavonoids | 52056, LMPK12120252 |
| 550.2036 | 550.2050 | 2.62 | 20.37 | Medioresinol 4'-O-beta-D-glucopyranoside | 531.26, 504.42, 327.09 |  | 1.6* ***(RRI)*** | Lignan | PMN |
| 552.1468 | 552.1479 | 0.00 | 23.18 | 5,2',5'-Trihydroxy-3,6,7,4'-tetramethoxyflavone 5'-glucoside | 367.16, 343.33 |  | 1.7*(RRC) | Flavonoids | 51455, LMPK12113043 |
| 566.1624 | 566.1636 | 1.00 | 23.18 | 5,7,4\'-Trihydroxyflavonone 4\'-O-xylosylglucoside | 367.16, 343.33 |  | 2.9***(RRC) | Flavonoids | 52740, LMPK12140252 |
| 566.1626 | 566.1636 | 1.00 | 19.62 | 5,7,4\'-Trihydroxyflavanone 7-O-arabinosylglucoside | 546.06, 504.14, 474.09, 444.14, 384.16, 354.21 |  | 1.7*(RRC) | Flavonoids | 52737, LMPK12140249 |
| 578.1616 | 578.1636 | 3.42 | 19.05 | Kaempferitrin | 559.3, 503.19, 473.30, 457.27, 383.32, 354.81, 337.21, 325.17 | 431.09, 430.08, 286.04, 285.03, 283.02 | 4.8*(RRC) | Flavonoids | 50281, LMPK12111865 |
| 578.1629 | 578.1636 | 1.10 | 14.34 | Kaempferol 3-rhamnoside-(1->2)-rhamnoside | 497.32 |  | 2.4*(RRC) | Flavonoids | 50323, LMPK12111907 |
| 592.1417 | 592.1428 | 1.00 | 21.64 | Kaempferol 3-[6\'\'-(3-hydroxy-3-methylglutaryl)glucoside] | 573.25, 561.32, 353.21 |  | 1.6*(RRC) | Flavonoids | 50201, LMPK12111785 |
| 674.1447 | 674.1424 | 0.00 | 21.66 | Phyllanthusmin B | 655.10, 477.26, 459.27 |  | 1.5*(RRC) | Lignan | C00031017 |
| 686.2743 | 686.2786 | 6.23 | 21.35 | Secoisolariciresinol di-O-glucoside | 670.58, 643.09, 625.27, 601.08, 583.10 |  | 1.46*(RRI), 4.1*(PRr) | Lignan | C00000652 |
| 710.2085 | 710.2058 | 3.73 | 25.01 | Kaempferol 3-rhamnoside-7-xylosyl-(1->2)-rhamnoside | 649.29, 634.93, 517.27, 443.32, 441.22, 367.04 |  | 34.9* (***RRI***) | Flavonoids | 50340 |
| 861.2420 | 861.2453 | 3.00 | 25.01 | Pelargonidin 3-rutinoside-7-(6-(p-hydroxybenzoyl)glucoside) | 819.24, 801.15, 777.15, 759.25, 685.11, 643.14 |  | 1.14*(RRI), 3.9**(PRr) | Flavonoids | 46849, LMPK12010082 |

**AME:** Accurate Mass Error= ((Observed mass - expected mass) / expected mass) X 10^6^, **RT:** Retention time, **RRC:** Resistance related constitutive, **PRr:** Pathogen related in resistant NIL, **RRI:** Resistance related induced, ***RRI***: Detected only in resistant NIL

**@ Fold change calculation**: were based on relative intensity of metabolites, RRC= RM/SM, PRr= RP/RM, RRI= (RP/RM)/(SP/SM); ***RRI***= RP/RM, PRr fold change is reported for the metabolites detected only in NIL-R. RP: resistant NIL with pathogen inoculation, RM: resistant NIL with mock inoculation, SP: susceptible NIL with pathogen inoculation, SM: susceptible NIL with mock inoculation.

***** *t* test significance at *P*<0.05, ****** *t* test significance at *P*<.01, *** *t* test significance at *P*<.001

**Database ID examples:** **Number**-METLIN, **LMP**-LIPIDMAPS, **KEGG**-C05610, **KNAPSACK**- C00002775, **MASSBANK**-PR051195, KOX00020, **NIKKAJI:** J355.695F, and **PMN**-Plant Metabolic Network, ***In silico:*** In silico fragmentation

**^$^**Database ID in bold is the fragmentation match

**References:**

Eklund P.C., Backman M.J., Kronberg L.Å., Smeds A.I., Sjöholm R.E. (2008) Identification of lignans by liquid chromatography‐electrospray ionization ion‐trap mass spectrometry. Journal of Mass Spectrometry 43:97-107.

Muroi A., Ishihara A., Tanaka C., Ishizuka A., Takabayashi J., Miyoshi H., Nishioka T. (2009) Accumulation of hydroxycinnamic acid amides induced by pathogen infection and identification of agmatine coumaroyltransferase in Arabidopsis thaliana. Planta 230:517-527.

Torras-Claveria L., Jáuregui O., Codina C., Tiburcio A.F., Bastida J., Viladomat F. (2011) Analysis of phenolics compounds by high-performance liquid chromatography coupled to electrospray ionization tandem mass spectrometry in senescent and water-stressed tobacco. Plant Science 182:71-78.
