# Supplementary material for: Integrated Metabolo-Proteomic Approach to Decipher the Mechanisms by Which Wheat QTL (Fhb1) Contributes to Resistance against Fusarium graminearum
Source: PLoS One. 2012 Jul 12;7(7):e40695. doi: 10.1371/journal.pone.0040695 (PMC3398977; doi:10.1371/journal.pone.0040695)
Supplement: Table S2 — Fusarium head blight resistance related metabolites identified in spikelets of wheat NIL with resistant Fhb1 allele following F. graminearum or mock inoculation. (DOC) [file pone.0040695.s006.doc]

**Table S2.** Fusarium head blight resistance related metabolites identified in ***spikelets*** of wheat NILs harboring *Fhb1* resistant allele, following *F. graminearum* or mock inoculation

| **Observed mass (Da)** | **Exact mass (Da)** | **AME** | **RT (min)** | **Putative name** | **Observed fragmentation** | **Database fragmentation** | **Fold change@** | **Chemical group** | **Database ID^$^** |
| --- | --- | --- | --- | --- | --- | --- | --- | --- | --- |
| 124.0519 | 124.0524 | 4.00 | 39.18 | 4-Methylcatechol | 108.33, 105.41, 80.97 | 108.02, 105.03, 80.02 | 1.2* (RRC) | Phenol | **64945,** C06730 |
| 131.0582 | 131.0582 | 4.00 | 16.15 | *trans*-Hydroxy-D-proline | **130.28, 112.03, 86.38, 62.17** | 130.05, 112.04, 86.02, 84.04, 68.04, 66.03, 57.03 | 1.50 (RRI), 59* (PRr) | Aldehyde | **257,** C01157 |
| 133.0371 | 133.0375 | 3.00 | 1.67 | L-aspartate | **132.23, 115.15, 114.19, 88.21** | 132.0, 115.0, 103.0, 88.0, 71.0 | 3.45 (RRI), 3.8* (PRr) | Amino acid | C00049, **PR051379**, C00001342 |
| 148.0532 | 148.0524 | 5.15 | 14.19 | *trans*-Cinnamic acid | **147.12,** 119.19, **103.05, 77.02** | 147, 103.05, 101.04, 77.04 | 2.0 (RRI), 1.7* (PRr) | Phenylpropanoid | **63104,** C00423, **PR050604, McGill MD** |
| 165.0797 | 165.0790 | 4.53 | 7.72 | L-phenylalanine | **164.01, 147.13, 103.16,** 91.15, **72.14** | 164.0, 147.0, 103.0, 72.0 | 1.56 (RRI), 2.5** (PRr) | Phenylpropanoid | **28,** C00079, **PR050152**, **McGill MD** C00001386 |
| 174.0172 | 174.0164 | 4.58 | 1.60 | *cis*-aconitate | **173.11,** 155.06, **129.0,** **110.99,** 70.97, **59.0** | 173.00, 129.01, 112.98, 111.00, 85.02, 59.01 | 2.32 (RRI), 11.6*** (PRr) | Carboxylic acid | C00417, **KOX00020** |
| 175.0639 | 175.0633 | 3.31 | 23.07 | Indole-3-acetate | **174.01,** 146.04, **130.15,** 129.09, 115.09 | 174.05, 130.06, 128.04 | 1.11 (RRI), 69.5* (PRr) | Auxin | 70, C00954, **KOX00380** |
| 178.0626 | 178.0630 | 1.00 | 22.49 | Coniferyl aldehyde | **177.03, 162.00,** 145.04, 117.99 | 177.0, 162.0, 134.0 | 1.08 (RRI), 72.2* (PRr) | Phenylpropanoid | C02666,  **PR051058**, **McGill MD** C00002728 |
| 182.0786 | 182.079 | 2.43 | 1.93 | D Mannitol | **163.14,** 131.17, **119.07**, **101.11, 89.18** | 181.07, 163.06, 119.03, 101.02, 89.02, 71.01, 59.04 | 15.31 (RRI), 19.9* (PRr) | Sugar | **142, C00392, PR051123** |
| 191.0588 | 191.0582 | 3.07 | 9.03 | 5-Hydroxyindoleacetic acid | 175.15, **146.16** | 146.06, 144.04, 131.03 | 1.31 (RRI), 2.5* (PRr) | Auxin | **44806,** C05635, **KO001068** |
| 208.0788 | 208.0736 | 4.00 | 23.62 | Sinapaldehyde | **207.08, 192.04,** 178.96, 164.92, 147.27, 121.33, 101.08 | 207.0, 192.0, 177.0,149.0, 93.0 | 3.36 (RRI), 20.2* (PRr) | Phenylpropanoid | **44806**, C05610, **McGill MD** |
| 210.1263 | 210.1256 | 3.25 | 16.65 | Jasmonic acid | 194.12, **191.01, 165.03,** 149.94, **143.01,** 127.18, 111.04 | 209.29, 191.00, 165.40, 143.05, 133.67 | 1.52 (RRI), 2.9* (PRr) | Fatty acid and conjugates | **62988,** C08491, **McGill MD** |
| 234.1375 | 234.1386 | 5.00 | 15.93 | *p*-Coumaroylputrescine | **233.11, 218.25,** 191.13,**119.01** | 119.04, 190.08, 218.11, 233.12 | 1.93 (RRI), 6.2* (PRr) | Hydroxycinnamaic acid | PMN, ***In silico,*** |
| 248.1419 | 248.1412 | 2.76 | 15.86 | Abscisic aldehyde | 247.29, 205.17, 119.00 | 219.38, 179.06 | 1.22 (RRI), 32.6* (PRr) | Terpenoid | C13455, PMN, *I****n silico*** |
| 250.1478 | 250.1569 | 5.00 | 16.17 | 4-Coumaroylcholine | 119.12 |  | 1.72 (RRI), 23.6* (PRr) | Phenylpropanoid | PMN |
| 254.0201 | 254.0192 | 3.72 | 1.40 | Shikimic acid 3-phosphate | 253.11, 237.82, 209.12, 193.04 |  | 1.3* (RRC) |  | 3384, C03175 |
| 272.0683 | 272.0685 | 1.00 | 22.97 | Naringenin | **271.10,** 270.05, 226.13, 203.16, 181.09, **118.86** | 271.0, 151.0, 119.0 | 1.2* (RRC) | Flavonoid | 3401, C00509, **PR051071,** C00000982, **McGill MD** |
| 276.1591 | 276.1586 | 1.8 |  | *cis-p*-Coumaroylagmatine | **258.29, 233.30, 119.18** | 119.04, 233.129, 258.14 | 41** (***RRI***) | Hydroxycinnamaic acid | PMN, ***In silico*** , |
| 278.2257 | 278.2246 | 4.08 | 30.66 | α-Linolenate | **277.22, 259.41,** 233.34, 80.16 | 277.21, 259.20, 59.01 | 2.79 (RRI), 3.2** (PRr) | Fatty acid and conjugates | **192**, C06426, C00001226, **McGill MD** |
| 280.2409 | 280.2402 | 2.39 | 31.39 | Linoleate | **279.40, 261.36, 259.27, 243.05,** | 279.4, 261.1, 259.3, 243.5, 219.5, 59.1 | 2.61 (RRI), 2.7* (PRr) | Fatty acid and conjugates | **191,** C01595, **KO001297,** **McGill MD** |
| 298.1207 | 298.1205 | 0.79 | 21.66 | Enterolactone |  |  | 1.47 (RRI), 52.5*** (PRr) | Lignan | 64734, C18165 |
| 316.1163 | 316.1158 | 1.50 | 14.89 | Vanilloloside | 297.31, 233.34, 201.32, 130.22 |  | 1.13 (RRI), 2.5* (PRr) | Phenylpropanoid | C00032471 |
| 318.1106 | 318.1103 | 0.94 | 26.03 | Catechin 7,4'-dimethyl ether | 328.12, 302.15, 286.38, 249.33 |  | 1.41 (RRI), 25.3** (PRr) | Flavonoid | 47359, LMPK12020143 |
| 322.1043 | 322.1053 | 3.00 | 13.35 | 4-O-Cinnamoylquinic acid | 277.14, 253.14, 249.31, 235.17, 176.15, 119.16 |  | 1.7* (RRC) | Phenylpropanoid | C00029541, ***In silico*** |
| 336.0836 | 336.0845 | 3.00 | 9.48 | 5-O-Caffeoylshikimic acid |  |  | 2.3** (RRC) | Phenylpropanoid | C00002720, |
| 338.0989 | 338.1002 | 4.00 | 1.98 | 1-Caffeoyl-4-deoxyquinic acid | 318.21, 294.38, 277.16, 153.33 |  | 2.7* (RRC) | Monolignol | C00002717 |
| 338.1282 | 338.1266 | 4.87 | 18.02 | Caffeoylserotonin | 322.27, 314.86, 307.27, 294.05, 217,13, 173.35 |  | 16.1 * (***RRI***) |  | ***In silico,*** PMN |
| 340.1315 | 340.1311 | 1.27 | 18.33 | 6-Prenylnaringenin | 324.16, 321.30, 271.41, 249.04, 163.22, 130.29 |  | 1.30 (RRI), 1.7* (PRr) | Flavonoid | 52761, LMPK12140277 |
| 342.1319 | 342.1315 | 1.25 | 14.13 | Coniferin | 341.33, 280.67, **179.03, 160.98,** 143.02, 113.12 | 179.03, 161 | 1.41 (RRI), 16.5* (PRr) | Phenylpropanoid | 64182, C00761 |
| 342.1683 | 342.1681 | 0.51 | 12.76 | 2-valeryl-sn-glycero-3-phosphocholine | 295.10, 281.10, 265.16 |  | 1.90 (RRI), 400.5* (PRr) | Glycerophospholipids | 40358, LMGP01050092 |
| 344.0851 | 344.0896 | 5.00 | 21.25 | 6-Hydroxykaempferol 3,7,4\'-trimethyl ether | 325.19, 257.33, 247.20, 195.20 |  | 2.22 (RRI), 9.8** (PRr) | Flavonoid | 51285, LMPK12112873 |
| 344.0898 | 344.0896 | 0.72 | 18.48 | Quercetin 3,5,3'-trimethyl ether | 325.25, 307.32, 289.35, 237.24, 229.18, 209.25, 201.15, 171.21 |  | 1.72 (RRI), 12.8** (PRr) | Flavonoid | 51167, LMPK12112753 |
| 344.1471 | 344.1471 | 0.12 | 15.75 | Iridotrial glucoside |  |  | 12.3* (***RRI***) | Terpenoid | C11653 |
| 344.1475 | 344.1471 | 1.33 | 17.33 | Dihydroconiferyl alcohol glucoside | 325.32, 275.28 |  | 1.16 (RRI), 2.9** (PRr) | Phenylpropanoid | C11652 |
| 344.1761 | 344.1776 | 4.00 | 25.69 | alpha,alpha\'-diethyl-4,4\'-bis(2-propynyloxy)stilbene | 328.18, 310.29, 292.39, 225.15, 171.24 |  | 1.4* (RRC) | Stilbene | 53252, C15066, LMPK13090025 |
| 350.1639 | 350.1630 | 2.45 | 16.40 | Vomilenine | 330.72, 304.09, 255.24, 233.22, 187.18 |  | 8.21 (RRI), 11.5** (PRr) | Alkaloids | 64326, C11680, |
| 352.1417 | 352.1423 | -1.78 | 22.75 | Feruloylserotonin | 336.32, 33.28, 201.16, 161.16, 135.22 |  | 30.7* (***RRI***) |  | PMN, J1.973.598B, ***In silico*** |
| 360.1410 | 360.1420 | 3.00 | 16.29 | 7-Deoxyloganate | 341.26, 291.19, 201.27, 171.25 |  | 1.30 (RRI), 31.6** (PRr) | Flavonoid | 64040 |
| 360.1576 | 360.1573 | 1.05 | 20.41 | Lariciresinol | 341.33, 315.28, **299.23,** 291.21 | 299, 284, 192, 178, 160 | 1.23 (RRI), 8.9* (PRr) | Lignan | C10646, |
| 370.1268 | 370.1264 | 1.05 | 14.07 | Sinapaldehyde glucoside | 360.21, 351.22, 249.29, 147.12 |  | 1.14 (RRI), 57.7* (PRr) | Phenylpropanoid | PMN |
| 372.1425 | 372.1420 | 1.16 | 13.03 | Syringin | **353.27,** 310.6, **249.26, 209.23,** 149.13 | 353, 311, 209 | 2.09 (RRI), 19.9* (PRr) | Phenylpropanoid | 64181, C01533 |
| 388.1161 | 388.1158 | 0.85 | 18.52 | Quercetagetin 5,6,7,3',4'-pentamethyl ether | 369.23, 265.16 |  | 6.4*** (RRC) | Flavonoid | 51431 |
| 388.1524 | 388.1522 | 0.59 | 20.65 | Trachelogenin/Medioresinol | 369.12, 351.09, 343.23, 327.16, 265.25, |  | 1.13 (RRI), 7* (PRr) | Lignan | 52090,  LMPK12120287 |
| 432.1420 | 432.1420 | 0.05 | 21.19 | Heptamethoxyflavone | 413.35, 341.13, 311.13, 269.18 |  | 1.11 (RRI), 1.9** (PRr) | Flavonoid | 49055, LMPK12110625 |
| 434.1212 | 434.1213 | 0.11 | 24.59 | Naringenin-7-O-Glucoside | 401.03, 328.11 |  | 1.83 (RRI), 2.2* (PRr) | Flavonoid | 52730, LMPK12140242 |
| 448.2452 | 448.2468 | 3.00 | 31.41 | Glutathionylaminopropylcadaverine | 279.37 |  | 1.2 (RRI), 4.2** (PRr) | Glutathione metabolism | 63644, C16566 |
| 476.1317 | 476.1319 | 0.35 | 25.22 | Naringenin 7-O-beta-D-glucoside 6\'\'-acetate | 328.04, 307.35, 289.25, 245.34, 209.25, 191.37 |  | 1.47 (RRI), 65.4* (PRr) | Flavonoid | 52745, LMPK12140257 |
| 478.1474 | 478.1475 | 0.17 | 14.35 | 2',4',4-Trihydroxy-3',3-dimethoxychalcone 4'-O-glucoside | 395.86, 297.54, 233.07 |  | 2.2* (RRC) | Flavonoid | 51984, LMPK12120180 |
| 478.1475 | 478.1475 | 0.09 | 26.14 | Persiconin | 459.11, 329.23, 309.20, 291.29, 209.24 |  | 2.04 (RRI), 44.2* (PRr) | Flavonoid | 53063, LMPK12140586 |
| 510.2962 | 510.2957 | 1.02 | 31.84 | 1-(9E-octadecenoyl)-sn-glycero-3-phospho-(1'-sn-glycerol) | 493.27, 423.29, 281.37 |  | 1.22 (RRI), 6** (PRr) | Glycerophospholipids | 40878, LMGP04050006 |
| 530.1396 | 530.1424 | 5.00 | 20.28 | Luteolin 7-glucoside-4\'-(Z-2-methyl-2-butenoate) | 510.67, 461.25 |  | 1.11 (RRI), 2.9* (PRr) | Flavonoid | 49214, LMPK12110784 |
| 550.1491 | 550.1475 | 2.96 | 16.50 | Formononetin 7-O-(2\'\'-p-hydroxybenzoylglucoside) | 531.37, 387.24, 369.25, 265.25 |  | 4.9*** (RRC) | Flavonoid | 47549, LMPK12050026 |
| 566.0553 | 566.0550 | 0.56 | 1.57 | UDP-glucose | 466.68, **384.94, 323.16, 240.98 211.20** | 565.04, 384.98, 323.02, 241.01, 78.95 | 1.61 (RRI), 2.1* (PRr) | carbohydrates and conjugates | **3598,** C00029 |
| 572.2962 | 572.2962 | 0.03 | 31.59 | 1-Hexadecanoyl-sn-glycero-3-phospho-(1'-myo-inositol) | 554.47, 485.18, 391.36, 315.19, 255.35, 241.10 |  | 1.67 (RRI), 10.5** (PRr) | Glycerophospholipids | 46744, LMGP06050002 |
| 598.3120 | 598.3118 | 0.38 | 31.69 | 1-(9Z-octadecenoyl)-sn-glycero-3-phospho-(1'-myo-inositol) | 417.27, 315.16, 281.46, 241.14, 223.05 |  | 1.10 (RRI), 10.9** (PRr) | Glycerophospholipids | 46747, LMGP06050005 |

**AME:** Accurate Mass Error= ((Observed mass - expected mass) / expected mass) X 10^6^, **RT:** Retention time, **RRC:** Resistance related constitutive, **PRr:** Pathogen related in resistant NIL, **RRI:** Resistance related induced, ***RRI***: Detected only in resistant NIL

**@ Fold change calculation**: were based on relative intensity of metabolites, RRC= RM/SM, PRr= RP/RM, RRI= (RP/RM)/(SP/SM); ***RRI***= RP/RM, PRr fold change is reported for the metabolites detected only in NIL-R. RP: resistant NIL with pathogen inoculation, RM: resistant NIL with mock inoculation, SP: susceptible NIL with pathogen inoculation, SM: susceptible NIL with mock inoculation.

***** *t* test significance at *P*<0.05, ****** *t* test significance at *P*<.01, *** *t* test significance at *P*<.001

**Database ID examples:** **Number**-METLIN, **LMP**-LIPIDMAPS, **KEGG**-C05610, **KNAPSACK**- C00002775, **MASSBANK**-PR051195, KOX00020, **NIKKAJI:** J355.695F, and **PMN**-Plant Metabolic Network, ***In silico:*** In silico fragmentation

**^$^**Database ID in bold is the fragmentation match

**References:**

Eklund P.C., Backman M.J., Kronberg L.Å., Smeds A.I., Sjöholm R.E. (2008) Identification of lignans by liquid chromatography‐electrospray ionization ion‐trap mass spectrometry. Journal of Mass Spectrometry 43:97-107.

Muroi A., Ishihara A., Tanaka C., Ishizuka A., Takabayashi J., Miyoshi H., Nishioka T. (2009) Accumulation of hydroxycinnamic acid amides induced by pathogen infection and identification of agmatine coumaroyltransferase in Arabidopsis thaliana. Planta 230:517-527.
