# Supplementary material for: Integrated Metabolo-Proteomic Approach to Decipher the Mechanisms by Which Wheat QTL (Fhb1) Contributes to Resistance against Fusarium graminearum
Source: PLoS One. 2012 Jul 12;7(7):e40695. doi: 10.1371/journal.pone.0040695 (PMC3398977; doi:10.1371/journal.pone.0040695)
Supplement: Table S3 — Resistant related induced (RRI) proteins in wheat NIL with resistant Fhb1 allele following F. graminearum inoculation. (DOC) [file pone.0040695.s007.doc]

**Table S3.** Resistant related induced proteins identified in rachis of wheat NILs with resistant *Fhb1* allele following *F. graminearum* inoculation.

| gi number | Protein name | Length | Mol Wt. (kDA) | RRI FC | Chromosomal location | GOs | KEGG Enzyme Codes | KEGG pathway |
| --- | --- | --- | --- | --- | --- | --- | --- | --- |
| 118040 | Cytochrome c | 112 | 12 | 2.39^**^ | — | generation of precursor metabolites and energy | - | Photosynthesis |
| 122006 | Histone H2A.1 | 150 | 16 | 1.35** | — | DNA binding; cellular component organization | - | DNA binding |
| 129806 | Peroxidase 1 | 315 | 33 | 3.00* | — | response to stress | EC:1.11.1.7 | Phenylpropanoid biosynthesis |
| 131394 | Oxygen-evolving enhancer protein 2 | 258 | 27 | 1.18* | 4AS, 4BL, 4DL, 2AS, 2BS, 2DS | photosynthesis | - |  |
| 170795 | Wali5 [*Triticum aestivum*] | 89 | 10 | 3.38** | 1AL, 1BL,1DL | - |  |  |
| 544242 | Endoplasmin homolog | 809 | 93 | 1.83** | — | anatomical structure morphogenesis; response to stress; cell death; signal transduction | EC:3.6.1.3 | Purine metabolism |
| 585032 | Cysteine synthase | 325 | 34 | 1.90* | — | transferase activity; photosynthesis; cellular amino acid and derivative metabolic process; | EC:2.5.1.52; EC:2.5.1.51; EC:2.5.1.47 | Cysteine and methionine metabolism |
| 974605 | Single-stranded nucleic acid binding protein [*Triticum aestivum*] | 167 | 16 | 1.35* | — | kinase activity; lipid metabolic process; response to stress; cell wall; cellular amino acid and derivative metabolic process; signal transduction | EC:2.7.11.0; EC:1.2.1.44 | Phenylpropanoid biosynthesis |
| 1170509 | Eukaryotic initiation factor 4A | 414 | 47 | 1.77** | 6AS | translation factor activity, RNA binding; anatomical structure morphogenesis; hydrolase activity; C:cell wall; | - |  |
| 1621627 | Manganese superoxide dismutase [*Triticum aestivum*] | 231 | 25 | 1.33* | 2AL, 2BL, 2DL | cellular component organization; cell death; response to stress; | EC:1.15.1.1 | oxidative stress response |
| 1657843 | Cold acclimation protein WCOR410b [*Triticum aestivum*] | 268 | 29 | 1.44* | 6AL, 6BL, 6DL | response to stress | - |  |
| 1706082 | Serine carboxypeptidase II-3 | 516 | 56 | 1.50** | — | hydrolase activity; response to endogenous stimulus; signal transduction | EC:3.4.16.0 |  |
| 2266662 | 14-3-3 protein [*Hordeum vulgare*] | 263 | 30 | 1.28** | 3AS, 3BS, 4AS, 4DS, 6BL, 6DL | cellular amino acid and derivative metabolic process; enzyme regulator activity; hydrolase activity; signal transduction; response to stress; lipid metabolic process | EC:3.6.1.0; EC:3.1.1.4 | alpha-Linolenic acid metabolism (regulatory protein of lipoxygenase |
| 2507469 | Triosephosphate isomerase, cytosolic | 253 | 27 | 1.23** | 3BS, 3DS, 2AS 2BS 2DS, | lipid metabolic process; carbohydrate metabolic process; generation of precursor metabolites and energy | EC:5.3.1.1 | Carbon fixation in photosynthetic organisms |
| 2759999 | Peroxidase [Hordeum vulgare] | 341 | 37 | 1.86* | 2AL, 2DL | response to stress; cell wall; | EC:1.11.1.7 | Phenylpropanoid biosynthesis |
| 3688398 | Ascorbate peroxidase [*Hordeum vulgare* subsp. *vulgare*] | 250 | 27 | 1.34** | 4AS, 4BL, 4DL | response to stress; | EC:1.11.1.11 | Glutathione metabolism, Ascorbate and aldarate metabolism |
| 3757682 | Glucan endo-1,3-beta-D-glucosidase [*Triticum aestivum*] | 335 | 35 | 1.67** | 1BL 1DL 7BS | response to stress; hydrolase activity; carbohydrate metabolic process; | EC:3.2.1.39 | PR protein |
| 4099148 | YLP [*Hordeum vulgare* subsp. *vulgare*] | 227 | 26 | 1.90** | 3AL, 3BL, 3DL | hydrolase activity; transporter activity; response to endogenous stimulus; signal transduction; cellular homeostasis; | EC:3.6.3.6 | Oxidative phosphorylation |
| 4995203 | Ribulose-bisphosphate carboxylase [*Heliocarpus americanus*] | 461 | 51 | 1.13** | — | photosynthesis; | EC:4.1.1.39 |  |
| 12328551 | Putative ribosomal protein L26 [*Oryza sativa* Japonica Group] | 157 | 17 | 1.72** | 7AS, 5BL | reproduction; transcription; RNA binding | - |  |
| 13925728 | Protein disulfide isomerase 3 precursor [*Triticum aestivum*] | 515 | 57 | 1.33* | 4AL, 1BS, 1DS | cell wall; response to stress; cell death; cellular homeostasis; cellular amino acid and derivative metabolic process; | EC:5.3.4.1; EC:1.14.11.2 |  |
| 14334165 | Pathogenesis-related protein 1 *[Triticum aestivum]* | 164 | 18 | 1.50** | 4BL, 4DL,5AL | response to stress; cell wall; | - | PR protein |
| 15224470 | ATPDX1.1 (pyridoxine biosynthesis 1.1); protein heterodimerization *[Arabidopsis thaliana]* | 309 | 33 | 5.25* | — | response to stress; | - |  |
| 18076790 | Phosphoglucomutase [*Triticum aestivum*] | 581 | 63 | 1.71* | — | generation of precursor metabolites and energy; | EC:5.4.2.2 | Glycolysis / Gluconeogenesis, Purine metabolism |
| 18146825 | Chitinase 1 [*Triticum aestivum*] | 256 | 27 | 3.00* | 1AL, 1DL | carbohydrate binding; response to biotic stimulus | - | PR protein |
| 20067423 | Glutathione transferase [*Triticum aestivum*] | 243 | 26 | 1.67* | 4AL, 7AS, 7DS | response to stress; transferase activity; cellular amino acid and derivative metabolic process; secondary metabolic process; | EC:2.5.1.18 | Glutathione metabolism |
| 20530127 | Mitochondrial aldehyde dehydrogenase [*Secale cereale*] | 549 | 59 | 2.33** | 7AS, 7BS, 7DS | response to biotic stimulus; cell-cell signaling; lipid binding; lipid metabolic process; cellular amino acid and derivative metabolic process | EC:1.2.1.5; EC:1.2.1.3; EC:1.2.1.36 | Tyrosine metabolism, |
| 22204116 | Putative cytochrome c oxidase subunit [*Triticum aestivum*] | 157 | 17 | 1.20** | 2AL, 2BL, 3AL, 3BS 3DS, 7BS | cellular component organization; transporter activity; membrane; response to stress; | EC:1.9.3.1 | Oxidative phosphorylation |
| 23504745 | Glutathione transferase F5 [*Triticum aestivum*] | 213 | 23 | 1.18** | 3DL, 4AL, 7AS 7BS | response to stress; transferase activity; cellular amino acid and derivative metabolic process | EC:1.11.1.9; EC:2.5.1.18 | Glutathione metabolism |
| 28192421 | Dehydroascorbate reductase [*Triticum aestivum*] | 212 | 23 | 1.31* | 1BS | response to biotic stimulus; peroxisome; receptor binding; cellular amino acid and derivative metabolic process; signal transduction | EC:1.8.5.1 | Ascorbate and aldarate metabolism, Glutathione metabolism |
| 30385246 | Caffeic acid O-methyltransferase [*Triticum aestivum*] | 360 | 39 | 1.30** | 3AL, 3DL, 7BL, 7DL | response to stress; transferase activity; | EC:2.1.1.68; EC:2.1.1.149; EC:2.1.1.76 | Phenylpropanoid biosynthesis |
| 34334012 | Cytosolic glutathione peroxidase [*Triticum monococcum*] | 168 | 18 | 2.10** | — | response to extracellular stimulus; cell communication; signal transduction; | EC:1.11.1.9; EC:1.11.1.12 | Glutathione metabolism, Arachidonic acid metabolism |
| 47607439 | Mitochondrial ATP synthase precursor [*Triticum aestivum*] | 238 | 27 | 1.71* | — | multicellular organismal development; | - |  |
| 48596901 | Ribosomal protein [*Bromus inermis*] | 265 | 30 | 1.63* | 1AS, 1DS | reproduction; transcription; | - |  |
| 50897038 | Methionine synthase [*Hordeum vulgare subsp. vulgare*] | 765 | 85 | 1.13** | 4AL,4BS, 4DS, 5DS | cellular amino acid and derivative metabolic process; transferase activity; response to stress; | EC:2.1.1.13; EC:2.1.1.14 | Cysteine and methionine metabolism |
| 52548242 | 20S proteasome beta 7 subunit [*Triticum aestivum*] | 215 | 24 | 1.94* | — | metabolic process; cell cycle; response to external stimulus; hydrolase activity; | - |  |
| 57635161 | Peroxidase 8 [*Triticum monococcum*] | 356 | 38 | 1.92* | — | Cell wall; response to biotic stimulus; | EC:1.11.1.7 | Phenylpropanoid biosynthesis |
| 68655500 | Methionine synthase 2 enzyme [*Hordeum vulgare subsp. vulgare*] | 766 | 84 | 1.14* | — | cellular amino acid and derivative metabolic process; transferase activity; response to stress; | EC:2.1.1.13; EC:2.1.1.14 | Cysteine and methionine metabolism |
| 71042078 | Chain C, Localization And Dynamic Behavior Of Ribosomal Protein L30e | 104 | 11 | 2.21* | 3DS | reproduction; transcription; multicellular | - |  |
| 71361902 | Glutamine synthetase isoform GS1b [*Triticum aestivum*] | 356 | 39 | 1.52* | — | cellular amino acid and derivative metabolic process; | EC:6.3.1.2 | Nitrogen metabolism |
| 77818928 | Flavonoid O-methyltransferase [*Triticum aestivum*] | 356 | 39 | 1.34** | 3AL, 3DL, 7BL, 7DL | response to external stimulus; Ptransferase activity; | EC:2.1.1.68; EC:2.1.1.149; EC:2.1.1.76 | Flavonoid metabolism |
| 82780762 | Putative steroid membrane binding protein [*Triticum aestivum*] | 223 | 24 | 1.46** | — | signal transduction; biological_process; enzyme regulator activity; response to endogenous stimulus; lipid metabolic process; | - |  |
| 90959771 | Multidomain cystatin [*Triticum aestivum*] | 243 | 27 | 1.72* | — | enzyme regulator activity; response to stress; | - |  |
| 109729547 | Germin-like protein 6a [*Hordeum vulgare* subsp. *vulgare]* | 219 | 23 | 1.56** | — | cell wall; signal transduction; | EC:1.15.1.1 | encoded by rice blast resistant QTL on chromosome 8 |
| 110288667 | Enolase, putative, expressed [*Oryza sativa* Japonica Group] | 480 | 52 | 1.21** | 5AS | response to biotic stimulus; | EC:4.2.1.11 | Glycolysis / Gluconeogenesis, Methane metabolism |
| 115434012 | Os01g0104400 [*Oryza sativa* Japonica Group] | 268 | 30 | 3.50** | 6BS | - |  |  |
| 115434536 | Os01g0149600 [*Oryza sativa* Japonica Group] | 153 | 17 | 1.40* | 7AS, 1DL | C:intracellular; F:DNA binding; P:cellular component organization; P:cellular process; F:protein binding; C:nucleus | - |  |
| 115435442 | Os01g0228600 [*Oryza sativa* Japonica Group] | 316 | 34 | 1.09* | 7AS, 1DL | F:catalytic activity | EC:1.1.1.0 |  |
| 115435850 | Os01g0267200 [*Oryza sativa* Japonica Group] | 307 | 34 | 4.17** | 7AS, 1DL | hydrolase activity; signal transduction; cell death; response to endogenous stimulus; protein modification process; cellular amino acid and derivative metabolic process; | EC:3.1.2.15 | ubiquitin thiolesterase |
| 115436314 | Os01g0328400 [*Oryza sativa* Japonica Group] | 155 | 18 | 1.67** | 7AS, 1DL | signal transduction; response to stress; cellular component organization, cell death; | - |  |
| 115436320 | Os01g0328700 [*Oryza sativa* Japonica Group] | 503 | 53 | 2.33** | 1AS | cellular amino acid and derivative metabolic process; anatomical structure morphogenesis; cellular homeostasis; | EC:1.8.1.4; EC:1.4.4.2; EC:1.2.4.2 | Citrate cycle (TCA cycle) |
| 115439261 | Os01g0686800 [*Oryza sativa* Japonica Group] | 334 | 36 | 2.00** | 1BL | cell death; cellular component organization; signal transduction; response to endogenous stimulus; | - |  |
| 115441607 | Os01g0896800 [*Oryza sativa* Japonica Group] | 304 | 35 | 1.40** | 2AS | reproduction; multicellular organismal development; anatomical structure morphogenesis; | - |  |
| 115443655 | Os02g0103700 [*Oryza sativa* Japonica Group] | 192 | 22 | 1.87* | 3BS | response to abiotic stimulus; | - |  |
| 115444937 | Os02g0205300 [*Oryza sativa* Japonica Group] | 424 | 47 | 5.00* | 6AS | cellular component organization; hydrolase activity; cell death; signal transduction; response to stress; cellular amino acid and derivative metabolic process; | EC:3.6.1.3 | Purine metabolism |
| 115447367 | Os02g0621700 [*Oryza sativa* Japonica Group] | 422 | 45 | 1.47* | 3BS | generation of precursor metabolites and energy; | EC:6.2.1.5; EC:6.2.1.4 | Citrate cycle (TCA cycle) |
| 115451853 | Os03g0243300 [*Oryza sativa* Japonica Group] | 402 | 42 | 3.00* | — | anatomical structure morphogenesis; signal transduction; response to biotic stimulus; | - |  |
| 115454135 | Os03g0617900 [*Oryza sativa* Japonica Group] | 415 | 45 | 3.00* | — | catalytic activity; biosynthetic process; P:cellular amino acid and derivative metabolic process; F:protein binding | EC:1.2.1.0 | Aminotransferase |
| 115457956 | Os04g0376000 [*Oryza sativa* Japonica Group] | 123 | 14 | 6.15** | — | P:reproduction; | - |  |
| 115460656 | Os04g0623800 [*Oryza sativa* Japonica Group] | 408 | 44 | 1.48* | — | transferase activity; cellular amino acid and derivative metabolic process; | EC:2.1.2.10; EC:1.4.4.2; EC:2.6.1.0 | Nitrogen metabolism |
| 115465581 | Os05g0574500 [*Oryza sativa* Japonica Group] | 221 | 25 | 1.17* | 1DL | regulation of gene expression, epigenetic; response to stress; cell wall; signal transduction; hydrolase activity; | - |  |
| 115467370 | Os06g0247500 [*Oryza sativa* Japonica Group] | 567 | 61 | 6.00* | — | kinase activity; carbohydrate metabolic process; | EC:2.7.1.11; EC:2.7.1.90 | Pentose phosphate pathway |
| 115470493 | Os07g0134800 [*Oryza sativa* Japonica Group] | 630 | 69 | 6.01* | 5AL | cell wall; generation of precursor metabolites and energy; cellular amino acid and derivative metabolic process | EC:4.3.1.17; EC:1.1.1.14; EC:1.3.5.1 | Cysteine and methionine metabolism |
| 115470967 | Os07g0188800 [*Oryza sativa* Japonica Group] | 534 | 57 | 1.33** | — | lipid binding; hydrolase activity; cellular amino acid and derivative metabolic process; | EC:1.2.1.27; | Valine, leucine and isoleucine degradation |
| 115471261 | Os07g0229900 [*Oryza sativa* Japonica Group] | 138 | 15 | 1.95** | 2AS | response to stress; structural molecule activity; | - |  |
| 115474481 | Os08g0113100 [*Oryza sativa* Japonica Group] | 336 | 36 | 1.63* | 5AL | kinase activity; secondary metabolic process | EC:2.7.1.15; EC:2.7.1.4 | Methane metabolism |
| 115477781 | Os08g0557600 [*Oryza sativa* Japonica Group] | 435 | 47 | 2.00* | — | response to stress; response to biotic stimulus; cellular homeostasis; | EC:1.6.5.4 |  |
| 115488436 | Os12g0443500 [*Oryza sativa* Japonica Group] | 480 | 53 | 5.00* | — | anatomical structure morphogenesis; carbohydrate metabolic process; | EC:1.1.1.22 | Ascorbate and aldarate metabolism |
| 115589742 | 5,10-methylene-tetrahydrofolate reductase [*Triticum monococcum*] | 582 | 65 | 1.20** | — | cellular amino acid and derivative metabolic process; | EC:1.5.1.20 | methyl transfer towards polyamine, lignin and ethylene biosynthesis |
| 115589748 | S-adenosylhomocysteine hydrolase [*Triticum monococcum*] | 42 | 5 | 1.18* | — | hydrolase activity; regulation of gene expression, epigenetic; response to stress; cellular amino acid and derivative metabolic process | EC:3.3.1.1 | Cysteine and methionine metabolism |
| 118136330 | Polyphenol oxidase [*Triticum aestivum*] | 577 | 64 | 3.13** | — | plastid | - |  |
| 119388709 | Alcohol dehydrogenase ADH1 [*Triticum monococcum* subsp. *aegilopoides*] | 379 | 41 | 1.80** | 1AS |  | EC:1.1.1.1 | Glycolysis / Gluconeogenesis, Tyrosine metabolism |
| 122220777 | S-adenosylmethionine synthase 3 | 394 | 43 | 1.99** | — | transferase activity; response to stress; response to abiotic stimulus; cellular amino acid and derivative metabolic process | EC:2.5.1.6 | Cysteine and methionine metabolism |
| 126723796 | Caffeoyl-CoA O-methyltransferase [*Bambusa oldhamii*] | 259 | 29 | 7.00** | 7AS, 5BL | transferase activity; response to stress; cellular amino acid and derivative metabolic process; | EC:2.1.1.104 | Phenylpropanoid biosynthesis |
| 158701881 | NADP-dependent malic enzyme [*Triticum aestivum*] | 570 | 63 | 1.33** | — | cellular component organization; transcription regulator activity; secondary metabolic process; response to stress; | EC:1.1.1.40; EC:1.1.1.39 | Carbon fixation in photosynthetic organisms |
| 162458737 | Cysteine synthase [*Zea mays*] | 325 | 34 | 14.67** | 2AL | transferase activity; photosynthesis; cellular amino acid and derivative metabolic process; | EC:2.5.1.52; EC:2.5.1.51; EC:2.5.1.47 | Cysteine and methionine metabolism |
| 162460088 | Ribosomal protein S27 [*Zea mays*] | 86 | 10 | 1.75* | 4AL, 4DL | cellular component organization; cell death; signal transduction; response to stress; DNA binding; enzyme regulator activity; | - |  |
| 162463282 | NADPH producing dehydrogenase of the oxidative pentose phosphate pathway [*Zea mays*] | 484 | 53 | 2.63** | 3AL, 3DL, 4AL, 7AS, 7DS, 2AS, 2DS, 4AS | carbohydrate metabolic process; secondary metabolic process; | EC:1.1.1.44 | Pentose phosphate pathway, Glutathione metabolism |
| 162463315 | Ribosomal proteinS6 [*Zea mays*] | 251 | 29 | 1.38** | 2AS, 2BS, 2DS | cellular component organization; cell death; anatomical structure morphogenesis; response to endogenous stimulus; signal transduction; | - |  |
| 194268461 | Chorismate synthase [*Hordeum vulgare* subsp. *vulgare*] | 442 | 47 | 1.67** | 4AS, 4DL | biosynthetic process; cellular amino acid and derivative metabolic process | EC:4.2.3.5 | Phenylpropanoid biosynthesis |
| 194295632 | Glutaredoxin [*Triticum aestivum*] | 113 | 12 | 6.60** | 2BS | response to stress; generation of precursor metabolites and energy; cellular homeostasis; cellular amino acid and derivative metabolic process; | EC:1.20.4.1 |  |
| 194339233 | Cyclophilin B-B [*Triticum aestivum*] | 213 | 23 | 1.25** | 1AL, 1BL, 1DL, 7AL 7BL, 7DL | multicellular organismal development; response to endogenous stimulus; signal transduction; | EC:5.2.1.8 |  |
| 194690134 | Unknown [*Zea mays*] /calreticulin-3-like | 532 | 60 | 2.25** | 6AS, 6BS, 6DS | cell wall; signal transduction; response to endogenous stimulus; cell death; | - | Ca signalling |
| 194700922 | Unknown [*Zea mays*] | 187 | 21 | 4.67* | 5AL, 5BL, 5DL | response to extracellular stimulus; cell communication; | - |  |
| 195613254 | Chlorophyll a-b binding protein 8 [*Zea mays*] | 267 | 29 | 7.50* | — | photosynthesis; | - | Photosynthesis |
| 195629344 | 60S ribosomal protein L2 [*Zea mays*] | 261 | 28 | 1.13** | — |  | - |  |
| 196051131 | Pathogenesis related protein 10 [*Triticum aestivum*] | 160 | 17 | 1.42** | 1BS 2AL 2BL 4AS | response to biotic stimulus; defense response; |  | PR protein |
| 218196143 | Hypothetical protein OsI_18555 *[Oryza sativa* Indica Group] | 502 | 53 | 4.50** | 1AS, 1BS, 1DS |  | EC:1.8.1.4; EC:1.4.4.2; EC:1.2.4.2 | Citrate cycle (TCA cycle) |
| 218202319 | Hypothetical protein OsI_31743 [*Oryza sativa* Indica Group] | 794 | 90 | 3.00* | — | response to stress; | - |  |
| 223635282 | S-adenosylmethionine synthase 1 | 394 | 43 | 2.60** | 7AS, 7BS, 7DS | transferase activity; response to stress; cellular amino acid and derivative metabolic process | EC:2.5.1.6 | Cysteine and methionine metabolism |
| 224551500 | Blue copper protein [*Triticum aestivum*] | 176 | 17 | 2.40** | 7DS, 2AL, 2BL | C:plasma membrane; C:membrane | - |  |
| 226493235 | LOC100281701 [*Zea mays*] | 584 | 61 | 2.33** | 4AL, 5BL, 5DL | response to stress; | - |  |
| 242052615 | Hypothetical protein SORBIDRAFT_03g011050 [*Sorghum bicolor*] | 365 | 40 | 1.67** | 6AL | response to stress; | EC:1.1.1.85; EC:1.1.1.41 | Citrate cycle (TCA cycle), Valine, leucine and isoleucine biosynthesis |
| 242080811 | Hypothetical protein SORBIDRAFT_07g005390 [*Sorghum bicolor*] | 996 | 10 | 1.33** | 4DL, 5AL | response to stress; | EC:4.2.1.3 | Citrate cycle (TCA cycle) |
| 242088805 | Hypothetical protein SORBIDRAFT_09g028230 [*Sorghum bicolor*] | 319 | 34 | 1.33** | — | response to stress; | - |  |
| 255637862 | Unknown [*Glycine max*] | 242 | 27 | 1.34* | 2BS,7AL, 7BL ,7DL | kinase activity; | EC:2.7.4.3 | Purine metabolism |
| 257209009 | Arabidopsis protein targeted to mitochondria proteins At5g10860 [*Saccharum* hybrid cultivar R570] | 205 | 23 | 1.43** | — | response to stress; response to abiotic stimulus; | - |  |
| 297598102 | Os01g0894300 [*Oryza sativa* Japonica Group] | 323 | 35 | 1.60* | — | kinase activity; carbohydrate metabolic process; | EC:2.7.1.15; EC:2.7.1.4 |  |
| 297809115 | Nucleoside diphosphate kinase 1 [*Arabidopsis lyrata* subsp. *lyrata*] | 148 | 16 | 3.00** | 1AS | kinase activity; esponse to external stimulus; signal transduction; | EC:2.7.4.6 | Pyrimidine metabolism |
| 298162735 | Cinnamyl alcohol dehydrogenase [*Triticum aestivum*] | 360 | 39 | 1.26** | — | Phenylpropanoid pathway | EC:1.1.1.195 | Phenylpropanoid biosynthesis |
| 302829372 | Heat shock protein Hsp70H [*Volvox carteri f. nagariensis*] | 618 | 67 | 9.01** | — | response to stress; response to abiotic stimulus; | - |  |

@RRI= (RP/RM)/(SP/SM). RP: resistant NIL with pathogen inoculation, RM: resistant NIL with mock inoculation, SP: susceptible NIL with pathogen inoculation, SM: susceptible NIL with mock inoculation
